# Rh(iii)-catalyzed C–H olefination of *N*-pentafluoroaryl benzamides using air as the sole oxidant†
†Electronic supplementary information (ESI) available: Data for new compounds and experimental procedures. CCDC 1042327. For ESI and crystallographic data in CIF or other electronic format see DOI: 10.1039/c4sc03350g


**DOI:** 10.1039/c4sc03350g

**Published:** 2015-01-08

**Authors:** Yi Lu, Huai-Wei Wang, Jillian E. Spangler, Kai Chen, Pei-Pei Cui, Yue Zhao, Wei-Yin Sun, Jin-Quan Yu

**Affiliations:** a Coordination Chemistry Institute , State Key Laboratory of Coordination Chemistry , School of Chemistry and Chemical Engineering , Nanjing National Laboratory of Microstructures , Collaborative Innovation Center of Advanced Microstructures , Nanjing University , Nanjing 210093 , China . Email: luyi@nju.edu.cn ; Email: sunwy@nju.edu.cn; b Department of Chemistry , The Scripps Research Institute , 10550 N. Torrey Pines Road , La Jolla , California 92037 , USA . Email: yu200@scripps.edu

## Abstract

The oxidative olefination of a broad array of arenes and heteroarenes with a variety of activated and unactivated olefins has be achieved *via* a rhodium(iii)-catalyzed C–H activation reaction. The use of an *N*-pentafluorophenyl benzamide directing group is crucial for achieving catalytic turnovers in the presence of air as the sole oxidant without using a co-oxidant.

## Abstract 2

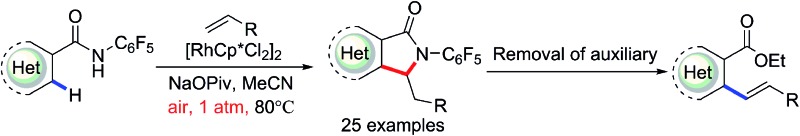
Rhodium(iii)-catalyzed C–H olefination reaction using air as the sole oxidant.

## Introduction

The direct coupling of unactivated aryl C–H bonds with olefins provides a step- and atom-economical method for the functionalization of arenes.^1^ While a number of transition metals have been explored in this capacity, Rh(iii) has emerged as an effective catalyst for the olefination of aryl C–H bonds under mild reaction conditions.^2^,^3^ Many research groups have sought to improve the utility of Rh(iii)-catalyzed aryl C–H olefinations by increasing reactivity of the catalytic systems and improving site selectivity of the transformation. This has been achieved *via* the use of proximal directing groups to promote *ortho*-C–H bond cleavage. However, in the previously reported Rh(iii)-catalyzed aryl C–H olefination reactions, stoichiometric oxidants such as peroxides, hypervalent iodonium salts, fluorinating agents, NXS (X = F, Cl, Br, or I) or inorganic salts are generally required to sustain the catalytic cycle.^4^ The use of any of these oxidants results in the undesired generation of stoichiometric reaction byproducts, reducing the utility and practicality of these transformations. An alternative approach has been the use of oxidizing directing groups CONHOR, which generally feature a cleavable N–O bond that can serve to reoxidize the Rh(i) catalyst.^5^ A greener and more environmentally benign approach is the use of molecular oxygen (O_2_) or air as the terminal oxidant, which generates water as the sole reaction byproduct.^6^ The goal of using O_2_ as the sole oxidant for C–H olefination reactions has been achieved in several cases using palladium.^7^ More recently Huang and coworkers reported the Rh(iii)-catalyzed C–H activation reactions using O_2_ as the sole oxidant in alkynylation reactions for the synthesis of isoquinolinium salts^8^ and indoles^9^ and the direct C-2 olefination of electron-rich indoles.^10^ However, to date, we are unaware of any other instances of a Rh(iii)-catalyzed C–H functionalization reaction of inert arenes using air as the sole oxidant without using a metal co-oxidant.3*u*

**Scheme 1:**
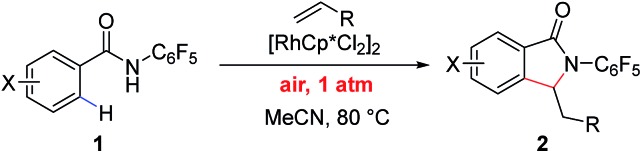
Aerobic Rh(iii)-catalyzed C–H olefination.

While the use of molecular O_2_ as oxidant is fundamentally important, the use of air as the sole oxidant for Rh(iii)/Rh(i) catalysis is practically desirable in terms of operational safety. Herein we report a Rh(iii)-catalyzed C–H activation/olefination reaction of aryl and heteroaryl benzamides under air at atmospheric pressure without the addition of an external oxidant. This reaction utilizes an *N*-pentafluorophenyl benzamide directing group, which has not previously been explored as a directing group for Rh(iii)-catalyzed reactions. The transformation is compatible with a range of olefin coupling partners, including simple styrenes (Scheme 1).

Our group has recently described the effectiveness of *N*-polyfluoroaryl benzamide directing groups in Pd(ii)-catalyzed C(sp^3^)–H activation/olefination reactions.^11^ We hoped to extrapolate the use of this directing group to Rh(iii)-catalyzed C–H olefination reactions. As shown in Table 1, treatment of pentafluorobenzamide **1a** and ethyl acrylate with 5 mol% [RhCp*Cl_2_]_2_ in the presence of AgOAc or Cu(OAc)_2_ as a stoichiometric oxidant provided product **2a** in good yield (Table 1, entries 1 and 2), verifying the efficacy of this directing group in rhodium-catalyzed transformations. Cyclization of the pentafluorophenyl benzamide onto the pendant enoate to form a γ-lactam product was previously described in a ruthenium-catalyzed oxidative C–H olefination.^12^ Acetonitrile was identified as the ideal solvent for achieving high levels of mono-selective olefination (see ESI†). However, when we conducted this reaction under an atmosphere of oxygen in the absence of an additional oxidant, only a trace amount of the desired product was observed (Table 1, entry 3).

**Table 1:** The olefination of aryl benzamide **1a** with ethyl acrylate

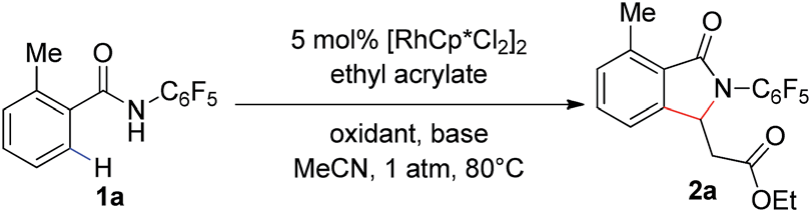			
Entry^*a*^	Oxidant	Base	Yield^*b*^ (%)
1	AgOAc	—	67
2	Cu(OAc)_2_	—	60
3	O_2_	—	Trace
4	O_2_	Na_2_CO_3_	27
5	O_2_	K_2_CO_3_	26
6	O_2_	NaOAc	64
7	O_2_	NaOPiv	99
8	Air	NaOPiv	92
**9** ^*c*^	**Air**	**NaOPiv**	**99(92)** ^*d*^
10^*e*^	Air	Na_2_CO_3_ + Boc–Leu–OH	93

^*a*^Reaction conditions: benzamide (0.2 mmol, 1.0 eq.), ethyl acrylate (0.5 mmol, 2.5 eq.), [RhCp*Cl_2_]_2_ (0.01 mmol, 0.05 eq.), oxidant (0.4 mmol, 2.0 eq.), base (0.2 mmol, 1.0 eq.), MeCN (2 mL), 80 °C, 24 h.

^*b*^Determined by ^1^H NMR analysis of the crude reaction mixture using CH_2_Br_2_ as the internal standard.

^*c*^[RhCp*Cl_2_]_2_ (0.004 mmol, 0.02 eq.), 48 h.

^*d*^Isolated yield.

^*e*^Amino acid (0.02 mmol, 0.1 eq.).

The addition of a base such as Na_2_CO_3_ or K_2_CO_3_ led to improved yields of the **2a** (Table 1, entries 4 and 5), indicating that the acidity of the polyfluorinated benzamide may be critical for the success of transformation in the absence of a stoichiometric oxidant. The use of a carboxylate base, such as NaOAc or NaOPiv, provided a dramatic improvement in reaction efficiency (Table 1, entries 6 and 7). As such, the reaction of benzamide **1a** with 5 mol% [RhCp*Cl_2_]_2_ and 1.0 eq. of NaOPiv under an atmosphere of O_2_ provided the desired product **2a** in 99% yield (Table 1, entry 7). Conducting the reaction under air instead of oxygen led to a slight decrease in product yield (Table 1, entry 8).‡
‡General procedure: to a 350 mL Schlenk-type sealed tube equipped with a magnetic stirring bar, were added the substrate (0.2 mmol, 1.0 eq.), [RhCp*Cl_2_]_2_ (0.01 mmol, 0.05 eq.), NaOPiv (0.2 mmol, 1.0 eq.), MeCN (2.0 mL) and olefin coupling partner (0.5 mmol, 2.5 eq.). The tube was capped and heated to 80 °C for 24 h. After cooling to room temperature, the reaction mixture was filtered through a pad of Celite. The filtrate was concentrated *in vacuo* to afford the crude product, which was purified by flash column chromatography (SiO_2_) gel to provide the desired product. However, an increased reaction time improved the yield of the transformation under air, under these reaction conditions product **2a** was formed in 99% yield with only 2 mol% catalyst (Table 1, entry 9, 92% isolated yield). Interestingly, we found that a similar level of reactivity could be obtained with Na_2_CO_3_ as the base with the addition of 10 mol% of a mono-protected amino acid ligand (MPAA) (Table 1, entry 10).^13^

With these optimized reaction conditions in hand we set out to analyze the scope of this transformation with respect to the benzamide substrate. *N*-Pentafluorophenyl benzamides with electron-donating (Table 2, entries **2a–b** and **2d**) or electron-withdrawing groups (Table 2, entries **2e–h**) react to provide corresponding olefinated products in excellent yields. Halide-substituted substrates, which bear a handle for further chemical manipulation, are also tolerated in this transformation (Table 2, entries **2i–n**). It is worth noting that the *meta*-substituted benzamides (Table 2, entries **2n** and **2o**) provide a regioisomeric mixture of olefination products. For *meta*-fluorinated substrate, the major product in most cases with Pd(OAc)_2_-catalyzed C–H activation occurs *para* to F with one exceptional example.^14^ Interestingly, *meta*-fluorinated substrate affords the *ortho* position olefination product **2n**, while *meta*-Me substrate affords the *para* position olefination product **2o**, illustrating that the acidity of the C–H bond plays a role in this C–H olefination reaction. The reaction can be conducted on gram scale with no decrease in isolated yield (Table 2, entry **2l**). The catalyst loading can also be lowered to 2 mol% [RhCp*Cl_2_]_2_ without significantly affecting the reactivity (Table 3).

**Table 2:** Scope of benzamide substrate^*a*^ ^,^^*b*^

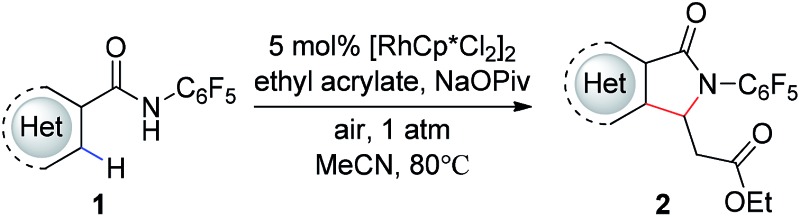
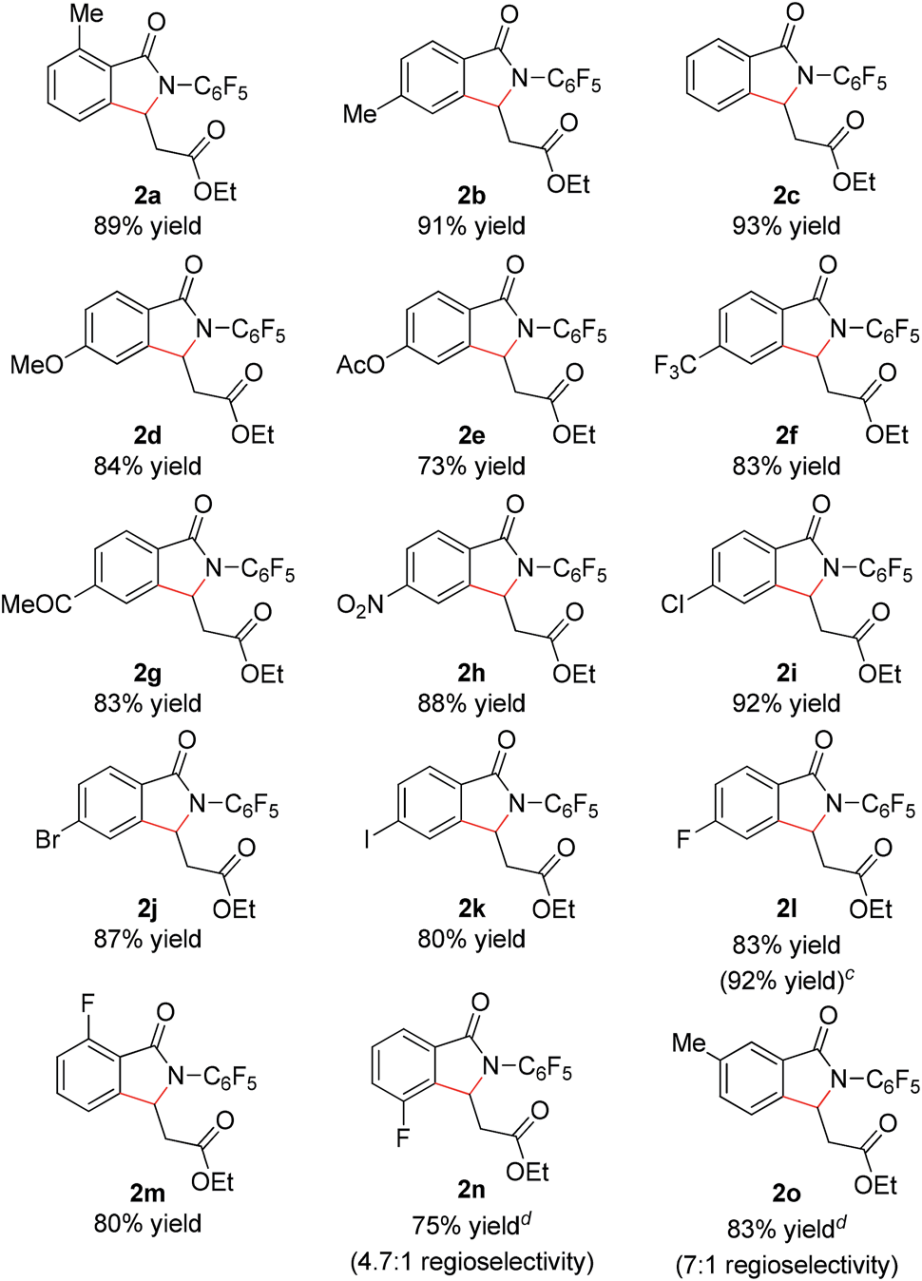
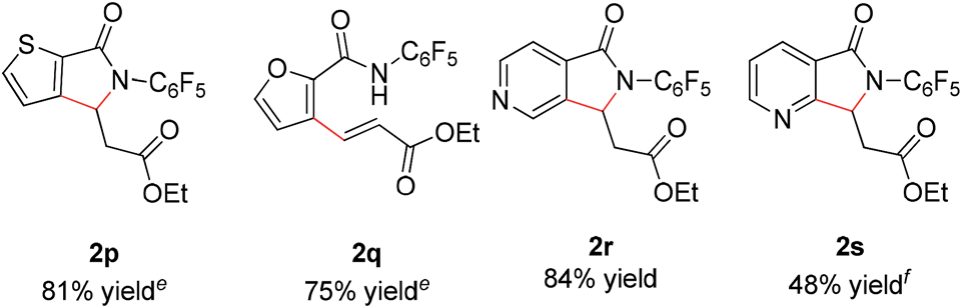

^*a*^Reaction conditions: benzamide (0.2 mmol, 1.0 eq.), ethyl acrylate (0.5 mmol, 2.5 eq.), [RhCp*Cl_2_]_2_ (0.01 mmol, 0.05 eq.), air, 1 atm, NaOPiv (0.2 mmol, 1.0 eq.), MeCN (2 mL), 80 °C, 24 h.

^*b*^Isolated yield.

^*c*^Reaction conducted on 3.5 mmol scale.

^*d*^isolated yields of the major isomers **2n** and **2o**.

^*e*^Reaction temperature is increased to 100 °C.

^*f*^Boc–Leu–OH (0.02 mmol, 0.1 eq.) and Na_2_CO_3_ (0.2 mmol, 1.0 eq.) instead of NaOPiv.

**Table 3:** Scope of benzamide substrate with 2% catalyst^*a*^ ^,^^*b*^

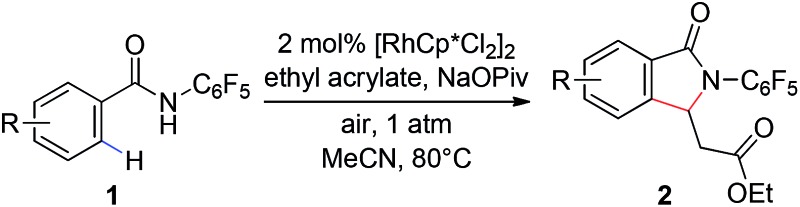
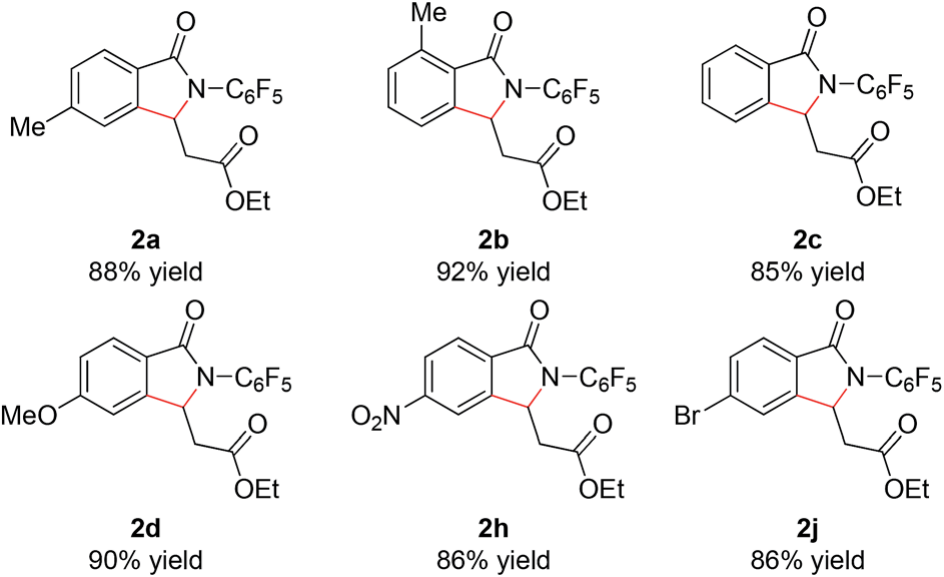

^*a*^Reaction conditions: benzamide (0.2 mmol, 1.0 eq.), ethyl acrylate (0.5 mmol, 2.5 eq.), [RhCp*Cl_2_]_2_ (0.004 mmol, 0.02 eq.), NaOPiv (0.2 mmol, 1.0 eq.), MeCN (2 mL), air, 1 atm, 80 °C, 48 h.

^*b*^Isolated yield.

In addition, we were pleased to find that heterocyclic amides, including thiophene, furan, and pyridine are competent substrates and can be olefinated in good yield (Table 3, entry **2p–2r**) with just a slight increase in reaction temperature. Interestingly, although the *N*-aryl thiophene-2-carboxamide reacts to provide the cyclized product **2p** in 81% yield (reaction time is increased to 48 hours), the *N*-aryl furan-2-carboxamide provides only the uncyclized product **2q** (75% yield) even with higher reaction temperatures and an extended reaction time. While olefination of pyridine-4-carboxamide gave the desired product **2r** in excellent yield, pyridine-3-carboxamide is less reactive, affording lower yield (Table 2, entry **2s**, 48%). 4-(Pyridine-2-yl)benzamide was also subjected to the standard conditions and the olefination occurred *ortho* to the amide rather than the pyridyl, albeit affording low yield (28%, see ESI†). Not surprisingly, pyridine-2-carboxamide is not reactive under these conditions due to the bis-dentate coordination of the substrate with Rh(iii).

We subsequently analyzed the scope of the olefin coupling partner in this transformation. As shown in Table 4, electron-deficient olefins, including ethyl vinyl ketone and acrylonitrile, can be coupled in good yield to provide lactam products **4a–b**. A variety of styrenes substituents are also coupled in good yields to provide the corresponding uncyclized products **5a–5c**. In addition, the unactivated olefin pentene can also be coupled, albeit in modest yield, to provide amide **5d**. However, di-substituted olefins are not reactive under these conditions.

**Table 4:** Scope of olefin coupling partner^*a*^ ^,^^*b*^

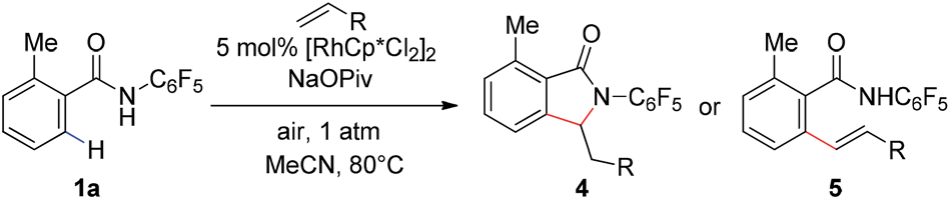
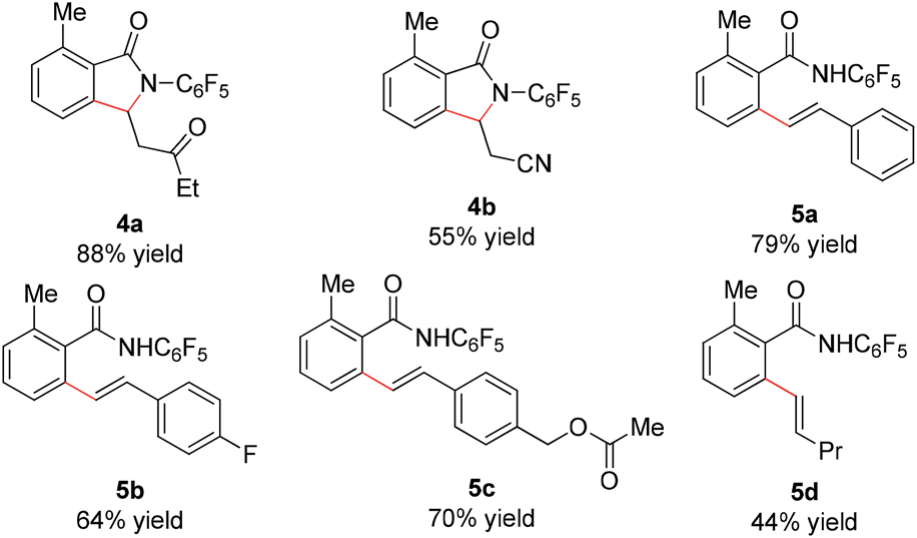

^*a*^Reaction conditions: benzamide (0.2 mmol, 1.0 eq.), olefins (0.5 mmol, 2.5 eq.), [RhCp*Cl_2_]_2_ (0.01 mmol, 0.05 eq.), NaOPiv (0.2 mmol, 1.0 eq.), MeCN (2 mL), air, 1 atm, 80 °C, 24 h.

^*b*^Isolated yield.

**Scheme 2:**
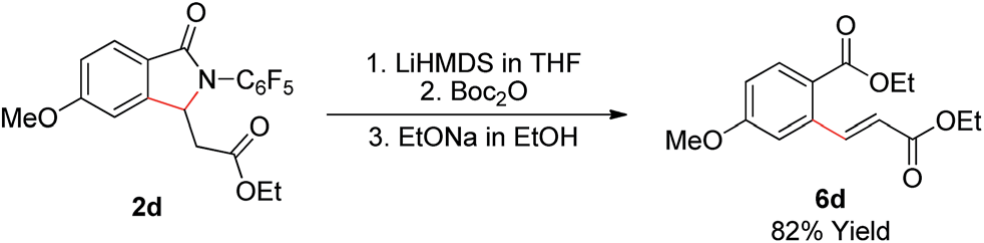
Removal of the auxiliary.

As shown in Scheme 2, the γ-lactam products formed in this transformation are readily converted to the olefinated products. Treatment of lactam **2d** with LiHMDS, Boc_2_O, and EtONa step by step in one-pot provides enoate **6d** in good yield.

**Scheme 3:**
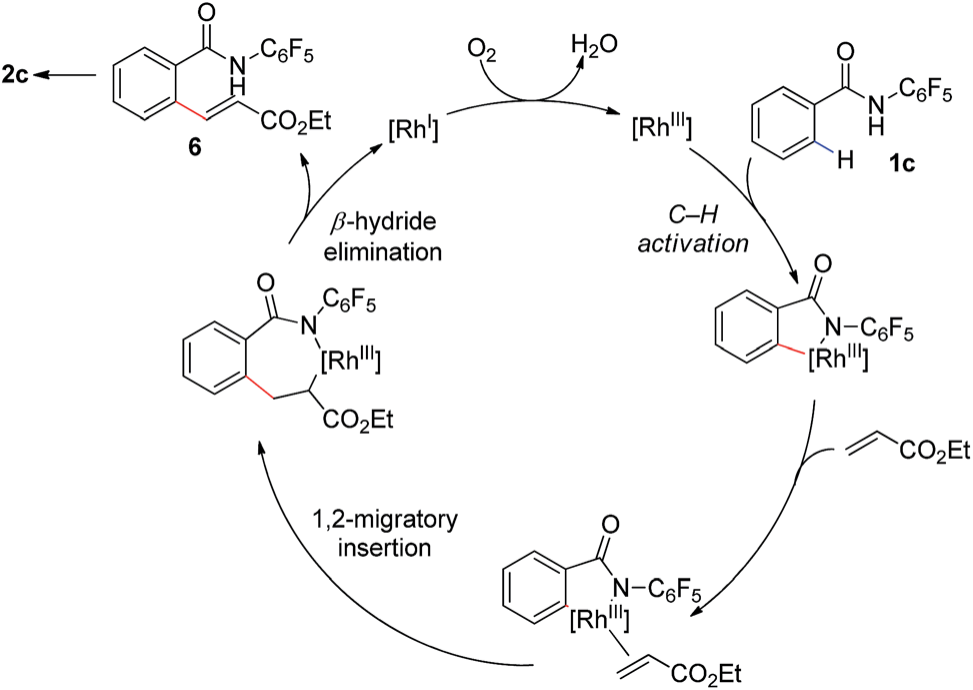
Proposed reaction mechanism.

Our proposed mechanism for this transformation is shown in Scheme 3. Thus coordination of the amide to the [Rh(iii)] catalyst is followed by *ortho*-C–H bond activation to give a corresponding [Rh(iii)–Ar] intermediate. Subsequent coordination to the olefin coupling partner and 1,2-migratory insertion provides an complex which can undergo β-hydride elimination to provide the uncyclized product **6**. Reoxidation of the [Rh(i)] to [Rh(iii)] by molecular oxygen would complete the catalytic cycle. This reoxidation step is likely facilitated by the acetate base, addition of which provides a substantial increase in reaction conversion. Subsequent base mediated 1,4-conjugate addition of the acidic *N*-pentafluorophenyl benzamide onto the pendant enoate provides the γ-lactam product **2**. The isolation of furan **2q** and alkenes **5a–5d**, which have undergone olefination but not cyclization, provide some evidence for the formation of **6** during the catalytic cycle.

In summary, we have developed a Rh(iii)-catalyzed C–H olefination of aryl C–H bonds using the *N*-pentaflurophenyl amide auxiliary and air as the sole oxidant. The reaction conditions can be applied to a variety of both aryl and heteroaryl benzamides.

## Supplementary Material

Supplementary information

Crystal structure data
