# Supplementary material for: Rh(iii)-catalyzed C–H olefination of N-pentafluoroaryl benzamides using air as the sole oxidant
Source: Chem Sci. 2015 Jan 8;6(3):1923–7. doi: 10.1039/c4sc03350g (PMC5810239; doi:10.1039/c4sc03350g)
Supplement: Supplementary file 1 [file SC-006-C4SC03350G-s001.pdf]

Electronic Supplementary Material (ESI) for Chemical Science.
This journal is © The Royal Society of Chemistry 2015

Supporting Information for

Rh(III)-Catalyzed C–H Olefination of N-Pentafluoroaryl Benzamides Using Air as the Sole Oxidant

Yi Lu[a]*, Huai-Wei Wang[a], Jillian E. Spangler[b], Kai Chen[a], Pei-Pei Cui[a], Yue Zhao[a], Wei-Yin Sun[a]*, and Jin-Quan Yu[b]*

[a]Coordination Chemistry Institute, State Key Laboratory of Coordination Chemistry, School of Chemistry and Chemical Engineering, Nanjing National Laboratory of Microstructures, Nanjing University, Nanjing 210093, China

[b]Department of Chemistry, The Scripps Research Institute, 10550 N. Torrey Pines Road, La Jolla, CA 92037, USA

*To whom correspondence should be addressed. Email: luyi@nju.edu.cn; sunwy@nju.edu.cn; yu200@scripps.edu

Table of Contents

General Information S3

Experimental Section

General procedure for the preparation of benzamides S4

Solvent optimization for C–H olefination S5

Ligand screening for C–H olefination S6

General procedure for C–H olefination S7

Procedure for the ring-opening/auxiliary removal S8

The effect of heterocycle in C–H olefination S9

References S11

Characterization of new synthesized compounds S12

NMR Spectra S26

General Information:

All solvents were used as received from commercial sources without further purification. Anhydrous solvents were prepared according to standard methods.[1] Double bond coupling partners and reagents used to prepare the substrates were purchased from Acros, Sigma-Aldrich, TCI, and Alfa and were used as received without further purification. Pentamethylcyclopentadienylrhodium (III) chloride dimer was purchased from Sigma-Aldrich and used without further purification. ^{1}H NMR and ^{13}C NMR spectra were recorded on Bruker-DRX (500 MHz and 125 MHz, respectively) and Bruker-DRX (400 MHz and 100 MHz, respectively) instruments internally referenced to $SiMe_4$ or chloroform signals. High resolution mass spectra were recorded at Center for Mass Spectrometry, Nanjing University.

Experimental Section

General procedure for the preparation of benzamides

Substrates are prepared according to literature procedure:[2] An acid chloride (20 mmol), prepared from the corresponding carboxylic acid and oxalyl chloride, was added to a vigorously stirring solution of 2,3,4,5,6-pentafluoroaniline (22 mmol) in toluene (50 mL). The reaction mixture was stirred for 24 h under reflux. After cooling to room temperature, the white precipitate was filtered off and washed with water, and recrystallized from toluene or ethyl acetate/hexane to give the products.

Solvent optimization for C–H olefination

Table S1. Solvent optimization for C–H olefination reaction[a,b]

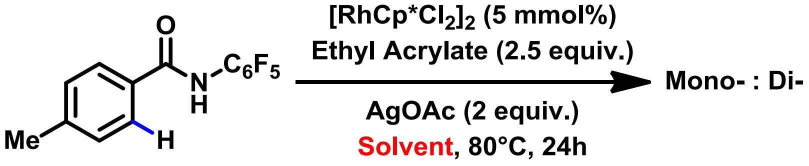

Entry	Solvent	Yield[b]
1	CH_2Cl_2	73% (11.2:1.0)
2	MeCN	87% [c]
3	DMF	86% (9.8:1.0)
4	1,4-dioxane	54% (4.4:1.0)
5	MeOH	75% (1.5:1.0)
6	Toluene	72% (0.5:1.0)

[a]: Benzamide (60.2 mg, 0.2 mmol), Ethyl acrylate (50.1 mg, 0.5 mmol), $[RhCp^*Cl_2]_2$ (6.2 mg, 0.01 mmol), AgOAc (66.8 mg, 0.4 mmol), Solvent (2 mL), 80℃, 24 hours; [b]: The yield was determined by ^{1}H NMR analysis of the crude reaction mixture using CH_2Br_2 as the internal stanard; the products consist of mono- and di-substitute ones (mono-:di-); [c]: The di-substituted product is trace.

To a 50 mL Schlenk-type sealed tube equipped with a magnetic stirring bar, were added the benzamide (60.2 mg, 0.2 mmol), $[RhCp^*Cl_2]_2$ (6.2 mg, 0.01 mmol, 5 mol%), AgOAc (66.8 mg, 0.4 mmol), Solvent (2.0 mL) and olefine coupling partner (0.5 mmol). The tube was heated to 80 °C for 24 hours and then cooled to room temperature. The reaction mixture was filtered through a pad of Celite and concentrated *in vacuo* to afford crude products. The yield was determined by ^{1}H NMR analysis of the crude reaction mixture using CH_2Br_2 as the internal standard.

Ligand screening for C–H olefination

Table S2. Ligand screening for C–H olefination reaction[a,b]

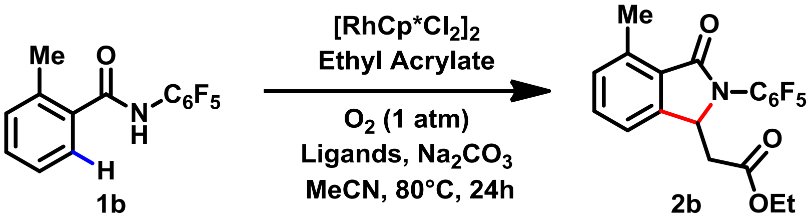

Entry	Ligands	Yield[b]	Entry	Ligands	Yield[b]
1	Cbz-Glu-OH	21%	9	Boc-Leu-OH	>99%
2	Ac-Glu-OH	14%	10	Boc-Phe-OH	68%
3	Fmoc-Leu-OH	87%	11	Boc-Pro-OH	64%
4	Boc-Asn-OH	67%	12	Boc-Tyr-OH	60%
5	Form-Leu-OH	50%	13	Boc-Val-OH	78%
6	Bz-Leu-OH	92%	14	Boc-Ala-OH	52%
7	Piv-Leu-OH	79%	15	Boc-Ile-OH	98%
8	Ac-Leu-OH	45%	16	Boc-Nle-OH	91%

[a]: Benzamide (60.2 mg, 0.2 mmol), $[RhCp^*Cl_2]_2$ (6.2 mg, 0.01 mmol), Ethyl acrylate (50.1 mg, 0.5 mmol), O_2 (1 atm), Ligands (0.02 mmol), Na_2CO_3 (21.2 mg, 0.2 mmol), MeCN (2 mL), 80 °C, 24 hours; [b]: The yield was determined by ^1H NMR analysis of the crude reaction mixture using CH_2Br_2 as the internal standard.

To a 50 mL Schlenk-type sealed tube equipped with a magnetic stirring bar, were added the benzamide (60.2 mg, 0.2 mmol), $[RhCp^*Cl_2]_2$ (6.2 mg, 0.01 mmol, 5 mol%), O_2 (1 atm), Ligand (0.02 mmol), MeCN (2.0 mL) and olefine coupling partner (0.5 mmol). The tube was heated to 80 °C for 24 hours and then cooled to room temperature. The reaction mixture was filtered through a pad of Celite and concentrated *in vacuo* to afford crude products. The yield was determined by ^1H NMR analysis of the crude reaction mixture using CH_2Br_2 as the internal standard.

General procedure for C–H olefination:

To a 350 mL Schlenk-type sealed tube equipped with a magnetic stirring bar, were added the substrate (0.2 mmol), $[RhCp^*Cl_2]_2$ (6.2 mg, 0.01 mmol), NaOPiv (28.4 mg, 0.2 mmol), MeCN (2.0 mL) and olefine coupling partner (0.5 mmol). The tube was capped, and heated to 80 °C for 24 hours. After cooled to room temperature, the reaction mixture was filtered through a pad of Celite. The filtrate was concentrated *in vacuo* to afford crude products, which was purified by flash column chromatography on silica gel to give the pure product.

Procedure for the ring-opening/auxiliary removal:

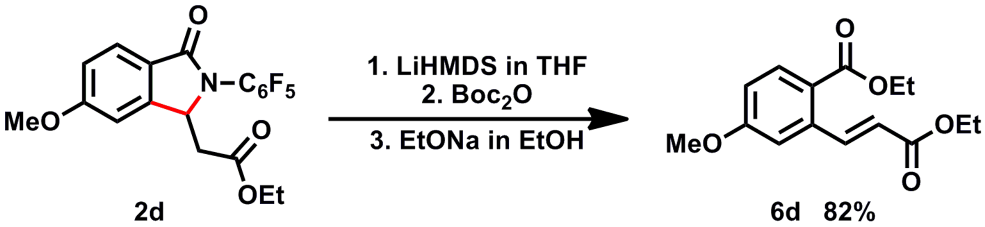

To an oven dried 10 mL round bottle flask equipped with a magnetic stir bar were added lactam (83.1mg, 0.2 mmol) and 1 mL of anhydrous THF. After cooling to -78℃, LiHMDS (1.0 M in THF, 5.0 equiv.) was added dropwise within 5 minutes. The mixture was warmed up to -20℃ naturally in 50 minutes. Then Boc_2O (6.0 equiv.) was added in -78 °C followed by warming up to 0 °C naturally in 2 hours. EtONa (1.0 M in EtOH, 10 equiv.) was added. After stirred at room temperature for 30 min, the reaction was quenched with saturated NH_4Cl/HOAc(10/1, 2mL). Extract with EtOAc (3*3 mL). The combined organic layer was washed with brine and dried over $MgSO_4$, filtrated and concentrated under vacuum, and purified by preparative TLC using hexanes/EtOAc (4/1) as the eluent to afford 45.6 mg of **6d** (82%) as white solid.

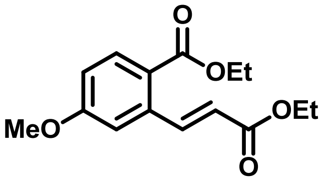

(E)-ethyl 2-(3-ethoxy-3-oxoprop-1-en-1-yl)-4-methoxybenzoate (**6d**):

^{1}H NMR (400 MHz, $CDCl_3$): δ 8.50 (d, J = 15.6 Hz, 1 H), 7.98 (d, J = 8.8 Hz, 1 H), 7.03 (d, J = 2.8 Hz, 1 H), 6.93 (dd, J_1 = 8.8 Hz, J_2 = 2.8 Hz, 1 H), 4.36 (q, J = 7.2 Hz, 2 H), 4.28 (q, J = 7.2 Hz, 2 H), 1.40 (t, J = 7.2 Hz, 3 H), 1.34 (t, J = 7.2 Hz, 3 H); ^{13}C NMR (125MHz, $CDCl_3$): δ 166.50, 166.36, 162.43, 144.35, 138.81, 133.05, 122.28, 121.01, 114.60, 112.97, 61.04, 60.54, 55.49, 14.27. HRMS (EI-TOF): m/z Calc. for $C_{15}H_{18}O_5Na$ $[M+Na]^+$: 301.1052, found 301.1050.

The effect of heterocycle in C–H olefination

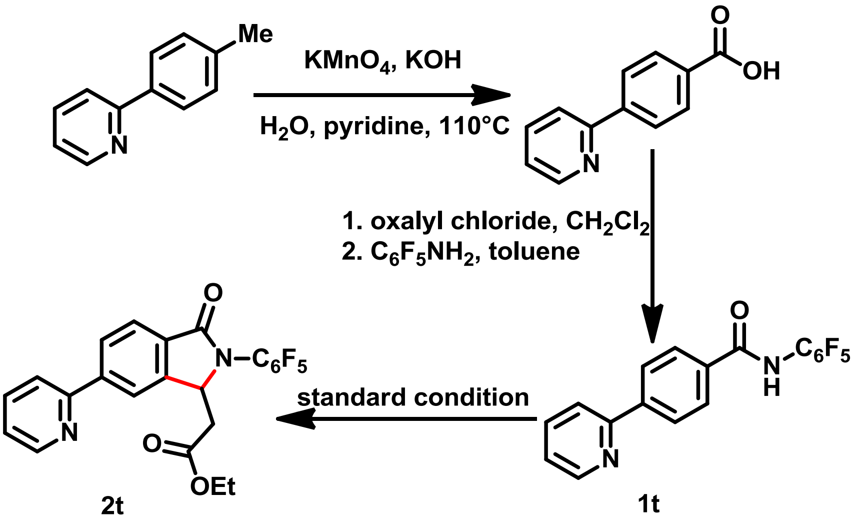

The acid (20 mmol) was prepared according previous report[3] and treated with oxalyl chloride to afford the acid chloride. And then, 2,3,4,5,6-pentafluoroaniline (22 mmol) in toluene (50 mL) was added and stirred for 24 h under reflux. After cooling to room temperature, the precipitate was filtered off and washed with ethyl acetate, water, and ethanol to give the substrate **1t**. Standard condition has been applied for **1t**, and the reaction mixture was concentrated *in vacuo* to afford crude products, which was purified by preparative TLC using EtOAc/Hexane (2/1) as the eluent to afford the major product **2t** (28%).

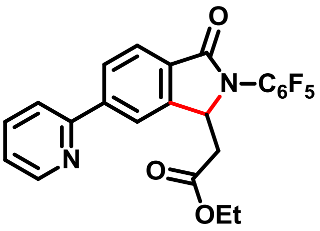

Ethyl 2-(3-oxo-2-(perfluorophenyl)-6-(pyridin-2-yl)isoindolin-1-yl)acetate (**2t**)

^{1}H NMR (500 MHz, $CDCl_3$): δ 8.75 (d, J = 4.8 Hz, 1 H), 8.26 (s, 1 H), 8.16 (dd, J_1 = 8.0 Hz, J_2 = 0.8 Hz, 1 H), 8.04 (d, J = 8.0 Hz, 1 H), 7.84–7.82 (m, 2 H), 7.35–7.32 (m, 1 H), 5.54 (t, J = 6.4 Hz, 1 H), 4.07–4.02 (m, 2 H), 2.95 (dd, J_1 = 16.4 Hz, J_2 = 5.6 Hz, 1 H), 2.76 (dd, J_1 = 16.4 Hz, J_2 =

7.2 Hz, 1 H), 1.18 (t, *J* = 7.2 Hz, 3 H); ^{13}C NMR (125 MHz, $CDCl_3$): δ 169.69, 166.93, 155.87, 150.01, 145.89, 144.28, 137.11, 130.10, 127.81, 125.04, 123.20, 121.38, 121.16, 61.34, 58.51, 38.75, 13.90. HRMS (EI-TOF): *m/z* Calc. for $C_{23}H_{15}F_5N_2O_3Na$ $[M+Na]^+$: 485.0901, found 485.0897.

References:

1. W. L. F. Armarego, D. D. Perrin, *Purification of Laboratory Chemicals* 4th Ed. Butterworth-Heinemann: Oxford, **1997**.

2. H. Ogita, Y. Isobe, H. Takaku, R. Sekine, Y. Goto, S. Misawa, H. Hayashi, *Bioorg. Med. Chem. Lett*. **2001**, *11*, 549.

3. H. Tang, Y. Li, C. Wei, B. Chen, W. Yang, H. Wu, Y. Cao, *Dyes and Pigments* **2011**, *91*, 413.

Characterization of new synthesized compounds

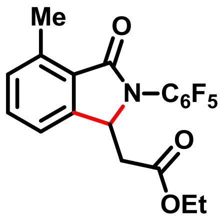

Ethyl 2-(4-methyl-3-oxo-2-(perfluorophenyl)isoindolin-1-yl)acetate (**2a**):

Following the general procedure, the C–H olefination was carried out with **1a** (60.2 mg, 0.2 mmol), $[RhCp^*Cl_2]_2$ (6.2 mg, 0.01 mmol), NaOPiv (28.4 mg, 0.2 mmol), MeCN (2.0 mL) and ethyl acrylate (50.1 mg, 0.5 mmol) at 80 °C for 24 hours. The product **2a** was obtained as white solid (71.0 mg, 89%) by flash column chromatography on silica gel using hexanes/EtOAc as the eluent. ^{1}H NMR (500 MHz, $CDCl_3$): δ 7.53 (t, J = 7.5 Hz, 1 H), 7.35 (d, J = 7.5 Hz, 1 H), 7.32 (d, J = 7.5 Hz, 1 H), 5.43 (t, J = 6.5 Hz, 1 H), 4.05 (q, J = 7.0 Hz, 2 H), 2.82 (dd, J_1 = 16.5 Hz, J_2 = 5.5 Hz, 1 H), 2.75 (s, 3 H), 2.70 (dd, J_1 = 16.5 Hz, J_2 = 7.0 Hz, 1 H), 1.19 (t, J = 7.0 Hz, 3 H); ^{13}C NMR (125 MHz, $CDCl_3$): δ 169.80, 167.81, 145.85, 144.99 (dd, J_1 = 257.8 Hz, J_2 = 35.2 Hz), 141.22 (d, J = 250.9 Hz), 139.03, 137.87 (d, J = 247.6 Hz), 132.56, 130.96, 126.97, 119.99, 112.00, 61.19, 57.54, 38.94, 17.35, 13.86. HRMS (EI-TOF): m/z Calc. for $C_{19}H_{14}F_5NO_3$ [M]: 399.0894, found 399.0888.

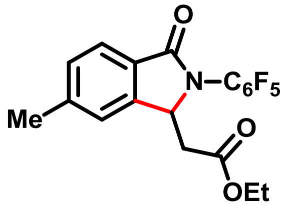

Ethyl 2-(6-methyl-3-oxo-2-(perfluorophenyl)isoindolin-1-yl)acetate (**2b**):

Following the general procedure, the C–H olefination was carried out with **1b** (60.2 mg, 0.2 mmol), $[RhCp^*Cl_2]_2$ (6.2 mg, 0.01 mmol), NaOPiv (28.4 mg, 0.2 mmol), MeCN (2.0 mL) and ethyl acrylate (50.1 mg, 0.5 mmol) at 80 °C for 24 hours. The product **2b** was obtained as white solid (72.6 mg, 91%) by flash column chromatography on silica gel using hexanes/EtOAc as the eluent. ^{1}H NMR (500 MHz, $CDCl_3$): δ 7.82 (d, J = 7.5 Hz, 1 H), 7.37 (d, J = 7.5 Hz, 1 H), 7.35 (s, 1 H), 5.42 (t, J = 6.5 Hz, 1 H), 4.05 (q, J = 7.0 Hz, 2 H), 2.81 (dd, J_1 = 16.5 Hz, J_2 = 6.0 Hz, 1 H),

2.69 (dd, J_1 = 16.5 Hz, J_2 = 7.0 Hz, 1 H), 2.50 (s, 3 H), 1.18 (t, J = 7.0 Hz, 3 H); ^{13}C NMR (125 MHz, $CDCl_3$): δ 169.74, 167.15, 144.98 (dd, J_1 = 250.5 Hz, J_2 = 39.1 Hz), 145.67, 144.11, 141.25 (d, J = 254.1 Hz), 137.86 (d, J = 250.9 Hz), 130.04, 127.22, 124.44, 123.12, 111.91, 61.18, 58.11, 38.65, 21.98, 13.83. HRMS (EI-TOF): *m/z* Calc. for $C_{19}H_{14}F_5NO_3$ [M]: 399.0894, found 399.0887.

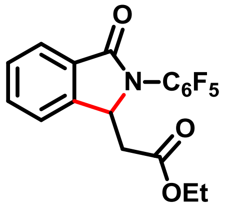

Ethyl 2-(3-oxo-2-(perfluorophenyl)isoindolin-1-yl)acetate (**2c**):

Following the general procedure, the C–H olefination was carried out with **1c** (57.4 mg, 0.2 mmol), $[RhCp^*Cl_2]_2$ (6.2 mg, 0.01 mmol), NaOPiv (28.4 mg, 0.2 mmol), MeCN (2.0 mL) and ethyl acrylate (50.1 mg, 0.5 mmol) at 80 °C for 24 hours. The product **2c** was obtained as white solid (71.6 mg, 93%) by flash column chromatography on silica gel using hexanes/EtOAc as the eluent. ^{1}H NMR (500 MHz, $CDCl_3$): δ 7.96 (d, J = 7.5 Hz, 1 H), 7.67 (t, J = 7.5 Hz, 1 H), 7.57 (t, J = 7.5 Hz, 1 H), 7.55 (d, J = 7.5 Hz, 1 H), 5.47 (t, J = 6.5 Hz, 1 H), 4.05 (q, J = 7.0 Hz, 2 H), 2.81 (dd, J_1 = 16.5 Hz, J_2 = 6.0 Hz, 1 H), 2.72 (dd, J_1 = 16.5 Hz, J_2 = 7.0 Hz, 1 H), 1.18 (t, J = 7.0 Hz, 3 H); ^{13}C NMR (125 MHz, $CDCl_3$): δ 169.67, 167.10, 144.95 (dd, J_1 = 250.0 Hz, J_2 = 38.8 Hz), 145.26, 141.36 (d, J = 255.1 Hz), 137.90 (d, J = 252.4 Hz), 133.09, 129.84, 129.08, 124.72, 122.74, 111.74, 61.26, 58.29, 38.58, 13.87. HRMS (EI-TOF): *m/z* Calc. for $C_{18}H_{12}F_5NO_3$ [M]: 385.0737, found 385.0765.

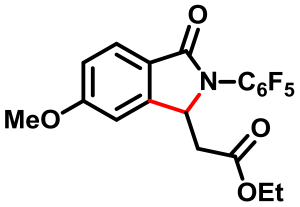

Ethyl 2-(6-methoxy-3-oxo-2-(perfluorophenyl)isoindolin-1-yl)acetate (**2d**):

Following the general procedure, the C–H olefination was carried out with **1d** (63.4 mg, 0.2 mmol), $[RhCp^*Cl_2]_2$ (6.2 mg, 0.01 mmol), NaOPiv (28.4 mg, 0.2 mmol), MeCN (2.0 mL) and

ethyl acrylate (50.1 mg, 0.5 mmol) at 80 °C for 24 hours. The product **2d** was obtained as white solid (69.7 mg, 84%) by flash column chromatography on silica gel using hexanes/EtOAc as the eluent. ^{1}H NMR (500 MHz, $CDCl_3$): δ 7.86 (d, J = 8.5 Hz, 1 H), 7.07 (d, J = 8.5 Hz, 1 H), 7.02 (s, 1 H), 5.39 (t, J = 6.0 Hz, 1 H), 4.05 (q, J = 7.0 Hz, 2 H), 3.90 (s, 3 H), 2.78 (dd, J_1 = 16.5 Hz, J_2 = 5.5 Hz, 1 H), 2.70 (dd, J_1 = 16.5 Hz, J_2 = 6.5 Hz, 1 H), 1.19 (t, J = 7.0 Hz, 3 H); ^{13}C NMR (125 MHz, $CDCl_3$): δ 169.74, 166.87, 163.90, 147.69, 144.95 (dd, J_1 = 257.2 Hz, J_2 = 39.2 Hz), 141.20 (d, J = 254.2 Hz), 137.82 (d, J = 247.0 Hz), 126.12, 122.16, 115.59, 111.91, 107.58, 61.21, 57.92, 55.69, 38.66, 13.83, 130.04, 127.22, 124.44, 123.12, 111.91, 61.18, 58.11, 38.65, 21.98, 13.83. HRMS (EI-TOF): m/z Calc. for $C_{19}H_{14}F_5NO_4$ [M]: 415.0843, found 415.0839.

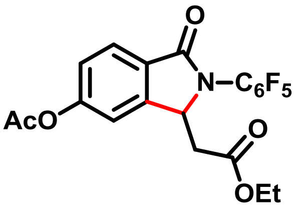

Ethyl 2-(6-acetoxy-3-oxo-2-(perfluorophenyl)isoindolin-1-yl)acetate (**2e**):

Following the general procedure, the C–H olefination was carried out with **1e** (69.0 mg, 0.2 mmol), $[RhCp^*Cl_2]_2$ (6.2 mg, 0.01 mmol), NaOPiv (28.4 mg, 0.2 mmol), MeCN (2.0 mL) and ethyl acrylate (50.1 mg, 0.5 mmol) at 80 °C for 24 hours. The product **2e** was obtained as white solid (64.7 mg, 73%) by flash column chromatography on silica gel using hexanes/EtOAc as the eluent. ^{1}H NMR (500 MHz, $CDCl_3$): δ 7.96 (d, J = 8.0 Hz, 1 H), 7.36 (s, 1 H), 7.29 (d, J = 8.5 Hz, 1 H), 5.45 (t, J = 6.5 Hz, 1 H), 4.06 (q, J = 7.0 Hz, 2 H), 2.80 (dd, J_1 = 16.5 Hz, J_2 = 6.5 Hz, 1 H), 2.73 (dd, J_1 = 16.5 Hz, J_2 = 6.5 Hz, 1 H), 2.35 (s, 3 H), 1.18 (t, J = 7.0 Hz, 3 H); ^{13}C NMR (125 MHz, $CDCl_3$): δ 169.53, 168.76, 166.19, 154.50, 146.78, 144.91 (dd, J_1 = 250.6 Hz, J_2 = 33.4 Hz), 141.42 (d, J = 253.9 Hz), 137.92 (d, J = 249.0 Hz), 127.25, 125.98, 122.93, 116.60, 111.53, 61.34, 57.97, 38.31, 21.04, 13.84. HRMS (EI-TOF): m/z Calc. for $C_{20}H_{14}F_5NO_5$ [M]: 443.0792, found 443.0785.

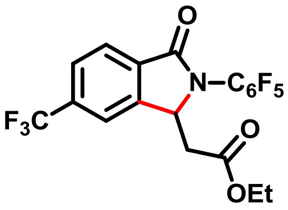

Ethyl 2-(3-oxo-2-(perfluorophenyl)-6-(trifluoromethyl)isoindolin-1-yl)acetate (**2f**):

Following the general procedure, the C–H olefination was carried out with **1f** (71.0 mg, 0.2 mmol), $[RhCp^*Cl_2]_2$ (6.2 mg, 0.01 mmol), NaOPiv (28.4 mg, 0.2 mmol), MeCN (2.0 mL) and ethyl acrylate (50.1 mg, 0.5 mmol) at 80 °C for 24 hours. The product **2f** was obtained as white solid (75.2 mg, 83%) by flash column chromatography on silica gel using hexanes/EtOAc as the eluent. ^{1}H NMR (500 MHz, $CDCl_3$): δ 8.09 (d, J = 8.0 Hz, 1 H), 7.87 (s, 1 H), 7.86 (d, J = 8.0 Hz, 1 H), 5.52 (t, J = 6.5 Hz, 1 H), 4.09 (q, J = 7.0 Hz, 2 H), 2.85-2.76 (m, 2 H), 1.20 (t, J = 7.0 Hz, 3 H); ^{13}C NMR (125 MHz, $CDCl_3$): δ 169.29, 165.68, 144.88 (dd, J_1 = 259.0 Hz, J_2 = 30.0 Hz), 145.65, 141.66 (d, J = 256.1 Hz), 138.00 (d, J = 252.4 Hz), 134.99 (q, J = 32.5 Hz), 133.09, 126.40, 125.41, 123.45 (d, J = 271.5 Hz), 120.36, 111.13, 61.52, 58.31, 38.12, 13.85. HRMS (EI-TOF): *m/z* Calc. for $C_{19}H_{11}F_8NO_3$ [M]: 453.0611, found 453.0614.

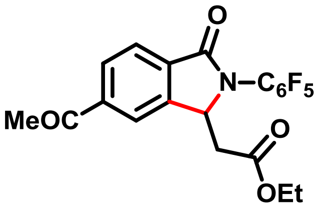

Ethyl 2-(6-acetyl-3-oxo-2-(perfluorophenyl)isoindolin-1-yl)acetate (**2g**):

Following the general procedure, the C–H olefination was carried out with **1g** (65.8 mg, 0.2 mmol), $[RhCp^*Cl_2]_2$ (6.2 mg, 0.01 mmol), NaOPiv (28.4 mg, 0.2 mmol), MeCN (2.0 mL) and ethyl acrylate (50.1 mg, 0.5 mmol) at 80 °C for 24 hours. The product **2g** was obtained as white solid (70.9 mg, 83%) by flash column chromatography on silica gel using hexanes/EtOAc as the eluent. ^{1}H NMR (500 MHz, $CDCl_3$): δ 8.17 (s, 1 H), 8.15 (d, J = 8.0 Hz, 1 H), 8.05 (d, J = 8.0 Hz, 1 H), 5.52 (t, J = 6.5 Hz, 1 H), 4.07 (q, J = 7.0 Hz, 2 H), 2.87 (dd, J_1 = 16.5 Hz, J_2 = 6.0 Hz, 1 H), 2.77 (dd, J_1 = 16.5 Hz, J_2 = 6.5 Hz, 1 H), 2.70 (s, 3 H), 1.19 (t, J = 7.0 Hz, 3 H); ^{13}C NMR (125 MHz, $CDCl_3$): δ 196.90, 169.32, 166.05, 145.44, 144.78 (dd, J_1 = 253.6 Hz, J_2 = 38.5 Hz), 141.51 (d, J = 255.1 Hz), 140.81, 137.90 (d, J = 252.9 Hz), 133.48, 129.27, 124.99, 122.59, 111.31, 61.41, 58.41, 38.22, 26.95, 13.86. HRMS (EI-TOF): *m/z* Calc. for $C_{20}H_{14}F_5NO_4$ [M]: 427.0843, found 427.0844.

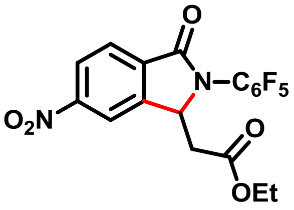

Ethyl 2-(6-nitro-3-oxo-2-(perfluorophenyl)isoindolin-1-yl)acetate (**2h**):

Following the general procedure, the C–H olefination was carried out with **1h** (66.4 mg, 0.2 mmol), $[RhCp^*Cl_2]_2$ (6.2 mg, 0.01 mmol), NaOPiv (28.4 mg, 0.2 mmol), MeCN (2.0 mL) and ethyl acrylate (50.1 mg, 0.5 mmol) at 80 °C for 24 hours. The product **2h** was obtained as white solid (75.7 mg, 88%) by flash column chromatography on silica gel using hexanes/EtOAc as the eluent. ^{1}H NMR (500 MHz, $CDCl_3$): δ 8.47 (s, 1 H), 8.46 (d, J = 8.0 Hz, 1 H), 8.13 (d, J = 8.0 Hz, 1 H), 5.55 (t, J = 6.5 Hz, 1 H), 4.11 (q, J = 7.0 Hz, 2 H), 2.84 (d, J = 6.5 Hz, 2 H), 1.21 (t, J = 7.0 Hz, 3 H); ^{13}C NMR (125 MHz, $CDCl_3$): δ 169.00, 164.88, 151.02, 146.21, 144.78 (dd, J_1 = 263.1 Hz, J_2 = 36.9 Hz), 141.80 (d, J = 254.1 Hz), 138.04 (d, J = 257.9 Hz), 135.05, 125.96, 124.71, 118.83, 110.82, 61.71, 58.27, 37.78, 13.91. HRMS (EI-TOF): *m/z* Calc. for $C_{18}H_{11}F_5N_2O_5$ [M]: 430.0588, found 430.0599.

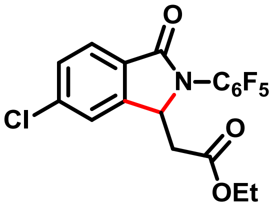

Ethyl 2-(6-chloro-3-oxo-2-(perfluorophenyl)isoindolin-1-yl)acetate (**2i**):

Following the general procedure, the C–H olefination was carried out with **1i** (64.2 mg, 0.2 mmol), $[RhCp^*Cl_2]_2$ (6.2 mg, 0.01 mmol), NaOPiv (28.4 mg, 0.2 mmol), MeCN (2.0 mL) and ethyl acrylate (50.1 mg, 0.5 mmol) at 80 °C for 24 hours. The product **2i** was obtained as white solid (77.1 mg, 92%) by flash column chromatography on silica gel using hexanes/EtOAc as the eluent. ^{1}H NMR (500 MHz, $CDCl_3$): δ 7.88 (d, J = 8.0 Hz, 1 H), 7.58 (s, 1 H), 7.55 (d, J = 8.0 Hz, 1 H), 5.43 (t, J = 6.5 Hz, 1 H), 4.08 (q, J = 7.0 Hz, 2 H), 2.81-2.71 (m, 2 H), 1.20 (t, J = 7.0 Hz, 3 H); ^{13}C NMR (125 MHz, $CDCl_3$): δ 169.38, 166.01, 146.81, 144.87 (dd, J_1 = 248.2 Hz, J_2 = 36.8 Hz), 141.48 (d, J = 255.1 Hz), 139.61, 137.94 (d, J = 252.2 Hz), 129.74, 128.33, 125.89, 123.44, 111.36,

61.41, 57.86, 38.20, 13.86. HRMS (EI-TOF): *m/z* Calc. for $C_{18}H_{11}ClF_5NO_3$ [M]: 419.0348, found 419.0343.

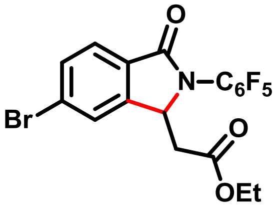

Ethyl 2-(6-bromo-3-oxo-2-(perfluorophenyl)isoindolin-1-yl)acetate (**2j**):

Following the general procedure, the C–H olefination was carried out with **1j** (73.0 mg, 0.2 mmol), $[RhCp^*Cl_2]_2$ (6.2 mg, 0.01 mmol), NaOPiv (28.4 mg, 0.2 mmol), MeCN (2.0 mL) and ethyl acrylate (50.1 mg, 0.5 mmol) at 80 °C for 24 hours. The product **2j** was obtained as white solid (80.6 mg, 87%) by flash column chromatography on silica gel using hexanes/EtOAc as the eluent. ^{1}H NMR (500 MHz, $CDCl_3$): δ 7.82 (d, J = 8.0 Hz, 1 H), 7.75 (s, 1 H), 7.72 (d, J = 8.0 Hz, 1 H), 5.43 (t, J = 6.5 Hz, 1 H), 4.08 (q, J = 7.0 Hz, 2 H), 2.81-2.71 (m, 2 H), 1.20 (t, J = 7.0 Hz, 3 H); ^{13}C NMR (125 MHz, $CDCl_3$): δ 169.38, 166.16, 146.94, 144.84 (dd, J_1 = 250.8 Hz, J_2 = 34.0 Hz), 141.49 (d, J = 255.5 Hz), 137.90 (d, J = 250.2 Hz), 132.63, 128.77, 128.01, 126.41, 126.05, 111.28, 61.44, 57.81, 38.20, 13.88. HRMS (EI-TOF): *m/z* Calc. for $C_{18}H_{11}BrF_5NO_3$ [M]: 462.9842, found 462.9846.

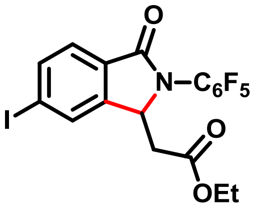

Ethyl 2-(6-iodo-3-oxo-2-(perfluorophenyl)isoindolin-1-yl)acetate (**2k**):

Following the general procedure, the C–H olefination was carried out with **1k** (82.6 mg, 0.2 mmol), $[RhCp^*Cl_2]_2$ (6.2 mg, 0.01 mmol), NaOPiv (28.4 mg, 0.2 mmol), MeCN (2.0 mL) and ethyl acrylate (50.1 mg, 0.5 mmol) at 80 °C for 24 hours. The product **2k** was obtained as white solid (81.8 mg, 80%) by flash column chromatography on silica gel using hexanes/EtOAc as the eluent. ^{1}H NMR (500 MHz, $CDCl_3$): δ 7.96 (s, 1 H), 7.93 (d, J = 8.0 Hz, 1 H), 7.68 (d, J = 8.0 Hz, 1 H), 5.41 (t, J = 6.5 Hz, 1 H), 4.07 (q, J = 7.0 Hz, 2 H), 2.78 (dd, J_1 = 16.5 Hz, J_2 = 6.5 Hz, 1 H),

2.72 (dd, J_1 = 16.5 Hz, J_2 = 6.5 Hz, 1 H), 1.20 (t, J = 7.0 Hz, 3 H); ^{13}C NMR (125 MHz, $CDCl_3$): δ 169.40, 166.40, 146.89, 144.89 (dd, J_1 = 250.6 Hz, J_2 = 42.0 Hz), 141.50 (d, J = 255.5 Hz), 138.49, 137.95 (d, J = 251.1 Hz), 132.29, 129.38, 126.02, 111.25, 61.45, 57.66, 38.26, 13.91. HRMS (EI-TOF): *m/z* Calc. for $C_{18}H_{11}F_5INO_3$ [M]: 510.9704, found 510.9709.

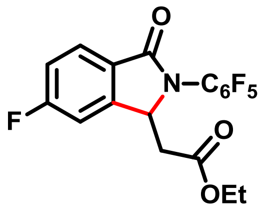

Ethyl 2-(6-fluoro-3-oxo-2-(perfluorophenyl)isoindolin-1-yl)acetate (**2l**):

Following the general procedure, the C–H olefination was carried out with **1l** (61.0 mg, 0.2 mmol), $[RhCp*Cl_2]_2$ (6.2 mg, 0.01 mmol), NaOPiv (28.4 mg, 0.2 mmol), MeCN (2.0 mL) and ethyl acrylate (50.1 mg, 0.5 mmol) at 80 °C for 24 hours. The product **2l** was obtained as white solid (66.9 mg, 83%) by flash column chromatography on silica gel using hexanes/EtOAc as the eluent. ^{1}H NMR (500 MHz, $CDCl_3$): δ 7.96-7.93 (m, 1 H), 7.28 (d, J = 8.5 Hz, 1 H), 7.27 (s, 1 H), 5.43 (t, J = 6.5 Hz, 1 H), 4.08 (q, J = 7.0 Hz, 2 H), 2.80-2.71 (m, 2 H), 1.20 (t, J = 7.0 Hz, 3 H); ^{13}C NMR (125 MHz, $CDCl_3$): δ 169.44, 166.00, 165.90 (d, J = 252.8 Hz), 147.81 (d, J = 10.1 Hz), 144.88 (dd, J_1 = 252.4 Hz, J_2 = 36.4 Hz), 141.44 (d, J = 255.6 Hz), 137.90 (d, J = 253.9 Hz), 126.96 (d, J = 9.8 Hz), 125.94, 117.04 (d, J = 23.4 Hz), 111.44, 110.53 (d, J = 24.5 Hz), 61.43, 57.88, 38.26, 13.89. HRMS (EI-TOF): *m/z* Calc. for $C_{18}H_{11}F_6NO_3$ [M]: 403.0643, found 403.0643.

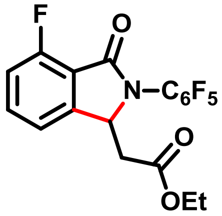

Ethyl 2-(4-fluoro-3-oxo-2-(perfluorophenyl)isoindolin-1-yl)acetate (**2m**):

Following the general procedure, the C–H olefination was carried out with **1m** (61.0 mg, 0.2 mmol), $[RhCp*Cl_2]_2$ (6.2 mg, 0.01 mmol), NaOPiv (28.4 mg, 0.2 mmol), MeCN (2.0 mL) and ethyl acrylate (50.1 mg, 0.5 mmol) at 80 °C for 24 hours. The product **2m** was obtained as white solid (64.5 mg, 80%) by flash column chromatography on silica gel using hexanes/EtOAc as the

eluent. ^{1}H NMR (500 MHz, $CDCl_3$): δ 7.67-7.63 (m, 1 H), 7.35 (d, J = 8.0 Hz, 1 H), 7.20 (t, J = 8.5 Hz, 1 H), 5.45 (t, J = 6.5 Hz, 1 H), 4.05 (q, J = 7.0 Hz, 2 H), 2.81 (dd, J_1 = 17.0 Hz, J_2 = 6.0 Hz, 1 H), 2.73 (dd, J_1 = 16.5 Hz, J_2 = 7.0 Hz, 1 H), 1.18 (t, J = 7.0 Hz, 3 H); ^{13}C NMR (125 MHz, $CDCl_3$): δ 169.42, 163.76, 159.28 (J = 261.6 Hz), 147.70, 144.96 (dd, J_1 = 253.8 Hz, J_2 = 40.4 Hz), 141.48 (d, J = 255.4 Hz), 137.88 (d, J = 256.5 Hz), 135.19 (d, J = 7.5 Hz), 118.74, 117.41 (d, J = 13.1 Hz), 116.37 (d, J = 18.9 Hz), 111.26, 61.36, 57.92, 38.49, 13.84. HRMS (EI-TOF): m/z Calc. for $C_{18}H_{11}F_6NO_3$ [M]: 403.0643, found 403.0642.

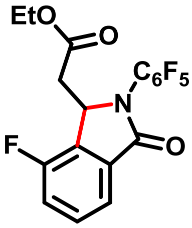

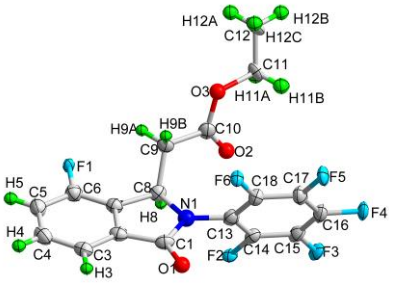

Ethyl 2-(5-fluoro-3-oxo-2-(perfluorophenyl)isoindolin-1-yl)acetate (**2n**):

Following the general procedure, the C–H olefination was carried out with **1n** (61.0 mg, 0.2 mmol), $[RhCp^*Cl_2]_2$ (6.2 mg, 0.01 mmol), NaOPiv (28.4 mg, 0.2 mmol), MeCN (2.0 mL) and ethyl acrylate (50.1 mg, 0.5 mmol) at 80 °C for 24 hours. The product **2n** was obtained as white solid (60.5 mg, 75%) by flash column chromatography on silica gel using hexanes/EtOAc as the eluent. ^{1}H NMR (500 MHz, $CDCl_3$): δ 7.77 (d, J = 7.5 Hz, 1 H), 7.58 (dd, J_1 = 12.5 Hz, J_2 = 8.0 Hz, 1 H), 7.36 (t, J = 8.5 Hz, 1 H), 5.60 (dd, J_1 = 8.0 Hz, J_2 = 3.0 Hz, 1 H), 4.01-3.91 (m, 2 H), 3.11 (dd, J_1 = 16.5 Hz, J_2 = 3.0 Hz, 1 H), 2.73 (dd, J_1 = 16.5 Hz, J_2 = 8.0 Hz, 1 H), 1.12 (t, J = 7.0 Hz, 3 H); ^{13}C NMR (125 MHz, $CDCl_3$): δ 169.09, 166.01, 158.44, 156.45, 144.86 (dd, J_1 = 248.8 Hz, J_2 = 45.8 Hz), 141.45 (d, J = 258.0 Hz), 137.87 (d, J = 241.5 Hz), 131.83 (d, J = 254.2 Hz), 131.3 (d, J = 5.9 Hz), 120.73, 119.88 (d, J = 19.6 Hz), 111.47, 61.27, 58.34, 37.06, 13.74. HRMS (EI-TOF): m/z Calc. for $C_{18}H_{11}F_6NO_3$ [M]: 403.0643, found 403.0642.

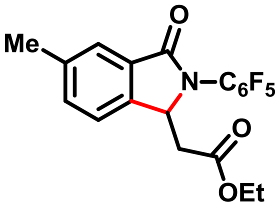

Ethyl 2-(4-methyl-3-oxo-2-(perfluorophenyl)isoindolin-1-yl)acetate (**2o**):

Following the general procedure, the C–H olefination was carried out with **1o** (60.2 mg, 0.2 mmol), $[RhCp^*Cl_2]_2$ (6.2 mg, 0.01 mmol), NaOPiv (28.4 mg, 0.2 mmol), MeCN (2.0 mL) and ethyl acrylate (50.1 mg, 0.5 mmol) at 80 °C for 24 hours. The product **2o** was obtained as white solid (66.2 mg, 83%) by flash column chromatography on silica gel using hexanes/EtOAc as the eluent. ^{1}H NMR (500 MHz, $CDCl_3$): δ 7.76 (s, 1 H), 7.47 (d, J = 7.5 Hz, 1 H), 7.42 (d, J = 7.5 Hz, 1 H), 5.42 (t, J = 6.5 Hz, 1 H), 4.05 (q, J = 7.0 Hz, 2 H), 2.77 (dd, J_1 = 16.5 Hz, J_2 = 6.0 Hz, 1 H), 2.69 (dd, J_1 = 16.5 Hz, J_2 = 6.5 Hz, 1 H), 2.48 (s, 3 H), 1.18 (t, J = 7.0 Hz, 3 H); ^{13}C NMR (125 MHz, $CDCl_3$): δ 169.76, 167.27, 144.95 (dd, J_1 = 245.7 Hz, J_2 = 36.2 Hz), 142.58, 141.34 (d, J = 251.1 Hz), 139.37, 137.93 (d, J = 249.9 Hz), 134.08, 129.94, 124.86, 122.47, 111.89, 61.23, 58.18, 38.73, 21.28, 13.91. HRMS (EI-TOF): *m/z* Calc. for $C_{19}H_{14}F_5NO_3$ [M]: 399.0894, found 399.0893.

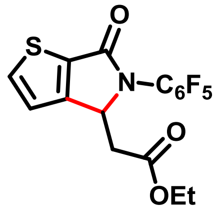

Ethyl 2-(6-oxo-5-(perfluorophenyl)-5, 6-dihydro-4*H*-thieno[2,3-*c*]pyrrol-4-yl)acetate (**2p**):

Following the general procedure, the C–H olefination was carried out with **1p** (58.6 mg, 0.2 mmol), $[RhCp^*Cl_2]_2$ (6.2 mg, 0.01 mmol), NaOPiv (28.4 mg, 0.2 mmol), MeCN (2.0 mL) and ethyl acrylate (50.1 mg, 0.5 mmol) at 100 °C for 24 hours. The product **2p** was obtained as white solid (63.3 mg, 81%) by flash column chromatography on silica gel using hexanes/EtOAc as the eluent. ^{1}H NMR (500 MHz, $CDCl_3$): δ 7.79 (d, J = 5.0 Hz, 1 H), 7.15 (d, J = 5.0 Hz, 1 H), 5.34 (t, J = 7.0 Hz, 1 H), 4.10 (q, J = 7.0 Hz, 2 H), 2.75 (dd, J_1 = 16.5 Hz, J_2 = 6.0 Hz, 1 H), 2.69 (dd, J_1 = 16.5 Hz, J_2 = 7.5 Hz, 1 H), 1.22 (t, J = 7.0 Hz, 3 H); ^{13}C NMR (125 MHz, $CDCl_3$): δ 169.47, 162.60, 156.44, 145.07 (dd, J_1 = 251.6 Hz, J_2 = 20.4 Hz), 141.43 (d, J = 254.1 Hz), 137.92 (d, J = 250.0 Hz), 136.92, 133.40, 121.64, 111.75, 61.31, 57.67, 37.66, 13.95. HRMS (EI-TOF): *m/z* Calc. for $C_{16}H_{10}F_5NO_3S$ [M]: 391.0302, found 391.0307.

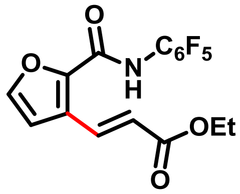

(E)-Ethyl 3-(2-((perfluorophenyl)carbamoyl)furan-3-yl)acrylate (**2q**):

Following the general procedure, the C–H olefination was carried out with **1q** (55.4 mg, 0.2 mmol), [RhCp*Cl_2]$_2$ (6.2 mg, 0.01 mmol), NaOPiv (28.4 mg, 0.2 mmol), MeCN (2.0 mL) and ethyl acrylate (50.1 mg, 0.5 mmol) at 100 °C for 24 hours. The product **2q** was obtained as white solid (56.3 mg, 75%) by flash column chromatography on silica gel using hexanes/EtOAc as the eluent. ^{1}H NMR (500 MHz, $CDCl_3$): δ 8.29 (d, J = 16.0 Hz, 1 H), 7.72 (s, 1 H), 7.52 (d, J = 1.5 Hz, 1 H), 6.80 (d, J = 1.5 Hz, 1 H), 6.36 (d, J = 16.0 Hz, 1 H), 4.25 (q, J = 7.0 Hz, 2 H), 1.31 (t, J = 7.0 Hz, 3 H); ^{13}C NMR (125 MHz, $CDCl_3$): δ 156.01, 144.63, 156.44, 143.39 (dd, J_1 = 251.0 Hz, J_2 = 53.1 Hz), 140.38 (d, J = 253.9 Hz), 137.88 (d, J = 250.4 Hz), 133.29, 128.70, 123.58, 110.60, 60.73, 14.23. HRMS (EI-TOF): m/z Calc. for $C_{16}H_{10}F_5NO_4$ [M]: 375.0530, found 375.0505.

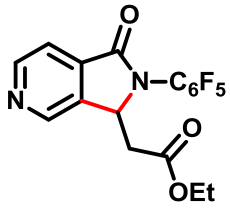

Ethyl 2-(1-oxo-2-(perfluorophenyl)-2,3-dihydro-1H-pyrrolo[3,4-c]pyridin-3-yl)acetate (**2r**):

Following the general procedure, the C–H olefination was carried out with **1r** (57.6 mg, 0.2 mmol), [RhCp*Cl_2]$_2$ (6.2 mg, 0.01 mmol), NaOPiv (28.4 mg, 0.2 mmol), MeCN (2.0 mL) and ethyl acrylate (50.1 mg, 0.5 mmol) at 80 °C for 24 hours. The product **2r** was obtained as white solid (64.6 mg, 84%) by flash column chromatography on silica gel using hexanes/EtOAc as the eluent. ^{1}H NMR (500 MHz, $CDCl_3$): δ 9.01 (s, 1 H), 8.91 (d, J = 4.5 Hz, 1 H), 7.86 (d, J = 5.0 Hz, 1 H), 5.58 (t, J = 6.5 Hz, 1 H), 4.10 (q, J = 7.0 Hz, 2 H), 2.82 (d, J = 7.0 Hz, 2 H), 1.21 (t, J = 7.0 Hz, 3 H); ^{13}C NMR (125 MHz, $CDCl_3$): δ 169.13, 165.42, 150.27, 145.48, 144.82 (dd, J_1 = 238.7 Hz, J_2 = 37.7 Hz), 141.74 (d, J = 255.5 Hz), 139.52, 138.03 (d, J = 254.2 Hz), 137.49, 118.13, 110.90, 61.58, 57.59, 37.89, 13.91. HRMS (EI-TOF): m/z Calc. for $C_{17}H_{12}F_5N_2O_3$ $[M+H]^+$: 387.0768, found 387.0761.

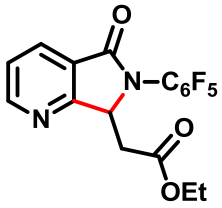

Ethyl 2-(5-oxo-6-(perfluorophenyl)-6,7-dihydro-5H-pyrrolo[3,4-b]pyridin-7-yl)acetate (**2s**)

Following the general procedure, the C–H olefination was carried out with **1s** (57.6 mg, 0.2 mmol), $[RhCp^*Cl_2]_2$ (6.2 mg, 0.01 mmol), NaOPiv (28.4 mg, 0.2 mmol), MeCN (2.0 mL) and ethyl acrylate (50.1 mg, 0.5 mmol) at 80 °C for 24 hours. The product **2s** was obtained as white solid (37.1 mg, 48%) by flash column chromatography on silica gel using hexanes/EtOAc as the eluent. ^{1}H NMR (400 MHz, $CDCl_3$): δ 8.85 (dd, J_1 = 5.0 Hz, J_2 = 1.4 Hz, 1 H), 8.38 (dd, J_1 = 7.6 Hz, J_2 = 1.6 Hz, 1 H), 8.38 (dd, J_1 = 7.8 Hz, J_2 = 5.0 Hz, 1 H), 5.47 (dd, J_1 = 7.6 Hz, J_2 = 3.6 Hz, 1 H), 4.03-3.95 (m, 2 H), 3.19 (dd, J_1 = 16.8 Hz, J_2 = 3.6 Hz, 1 H), 2.79 (dd, J_1 = 16.8 Hz, J_2 = 7.6 Hz, 1 H), 1.13 (t, J = 6.8 Hz, 3 H); ^{13}C NMR (125 MHz, $CDCl_3$): δ 169.31, 165.61, 164.56, 153.90, 144.81 (dd, J_1 = 252.8 Hz, J_2 = 46.3 Hz), 141.50 (d, J = 255.4 Hz), 137.89 (d, J = 251.5 Hz), 132.75, 123.93, 111.38, 61.21, 59.90, 36.83, 13.79. HRMS (EI-TOF): *m/z* Calc. for $C_{17}H_{12}F_5N_2O_3$ $[M+H]^+$: 387.0768, found 387.0761.

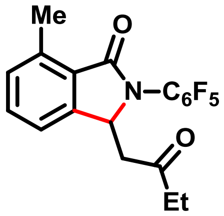

7-Methyl-3-(2-oxobutyl)-2-(perfluorophenyl)isoindolin-1-one (**4a**)

Following the general procedure, the C–H olefination was carried out with **1a** (60.2 mg, 0.2 mmol), $[RhCp^*Cl_2]_2$ (6.2 mg, 0.01 mmol), NaOPiv (28.4 mg, 0.2 mmol), MeCN (2.0 mL) and ethyl vinyl ketone (42.0 mg, 0.5 mmol) at 80 °C for 24 hours. The product **4a** was obtained as white solid (67.4 mg, 88%) by flash column chromatography on silica gel using hexanes/EtOAc as the eluent. ^{1}H NMR (400 MHz, $CDCl_3$): δ 7.49 (t, J = 7.6 Hz, 1 H), 7.29-7.25 (m, 2 H), 5.51 (t, J = 6.4 Hz, 1 H), 2.91 (dd, J_1 = 18.0 Hz, J_2 = 6.0 Hz, 1 H), 2.83 (dd, J_1 = 18.0 Hz, J_2 = 6.0 Hz, 1 H), 2.73 (s, 3 H), 2.38 (q, J = 7.2 Hz, 2 H), 1.00 (t, J = 7.6 Hz, 3 H); ^{13}C NMR (100 MHz, $CDCl_3$): δ 207.78, 168.09, 146.60, 144.88 (d, J = 254.3 Hz), 141.21 (d, J = 254.2 Hz), 138.95, 137.89 (d, J =

246.0 Hz), 132.55, 130.79, 126.88, 120.04, 112.32 (t, J = 14.8 Hz), 56.63, 46.58, 36.41, 17.36, 7.49. HRMS (EI-TOF): *m/z* Calc. for $C_{19}H_{14}F_5NO_2$ [M]: 383.0945, found 383.0951.

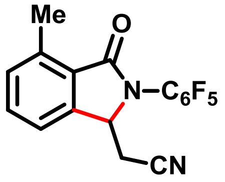

2-(4-Methyl-3-oxo-2-(perfluorophenyl)isoindolin-1-yl)acetonitrile (**4b**)

Following the general procedure, the C–H olefination was carried out with **1a** (60.2 mg, 0.2 mmol), $[RhCp^*Cl_2]_2$ (6.2 mg, 0.01 mmol), NaOPiv (28.4 mg, 0.2 mmol), MeCN (2.0 mL) and acrylonitrile (26.5 mg, 0.5 mmol) at 80 °C for 24 hours. The product **4b** was obtained as white solid (38.7 mg, 55%) by flash column chromatography on silica gel using hexanes/EtOAc as the eluent. ^1H NMR (400 MHz, $CDCl_3$): δ 7.60 (t, J = 7.6 Hz, 1 H), 7.49 (d, J = 7.6 Hz, 1 H), 7.38 (d, J = 7.6 Hz, 1 H), 5.18 (t, J = 5.8 Hz, 1 H), 2.82 (d, J = 6.0 Hz, 2 H), 2.75 (s, 3 H); ^{13}C NMR (100 MHz, $CDCl_3$): δ 167.24, 145.29 (d, J = 246.6 Hz), 143.45, 141.68 (d, J = 255.5 Hz), 139.65, 137.95 (d, J = 259.1 Hz), 133.10, 131.96, 126.75, 119.94, 115.06, 110.84 (t, J = 15.6 Hz), 56.48, 22.76, 17.41. HRMS (EI-TOF): *m/z* Calc. for $C_{17}H_9F_5N_2O$ [M]: 352.0635, found 352.0640.

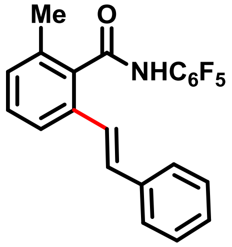

(E)-2-Methyl-N-(perfluorophenyl)-6-styrylbenzamide (**5a**)

Following the general procedure, the C–H olefination was carried out with **1a** (60.2 mg, 0.2 mmol), $[RhCp^*Cl_2]_2$ (6.2 mg, 0.01 mmol), NaOPiv (28.4 mg, 0.2 mmol), MeCN (2.0 mL) and styrene (52.0 mg, 0.5 mmol) at 80 °C for 24 hours. The product **5a** was obtained as white solid (63.7 mg, 79%) by flash column chromatography on silica gel using hexanes/EtOAc as the eluent. ^1H NMR (400 MHz, $CDCl_3$): δ 7.49 (d, J = 8.0 Hz, 1 H), 7.44 (t, J = 8.0 Hz, 3 H), 7.34 (t, J = 7.4 Hz, 2 H), 7.29 (d, J = 8.0 Hz, 1 H), 7.17 (d, J = 16.0 Hz, 1 H), 7.08 (d, J = 7.6 Hz, 1 H), 7.01 (d, J = 16.4 Hz, 1 H), 2.31 (s, 3 H); ^{13}C NMR (100 MHz, $CDCl_3$): δ 168.54, 143.31 (d, J = 248.4 Hz),

140.43 (d, J = 252.5 Hz), 137.70 (d, J = 255.5 Hz), 136.66, 135.02, 134.46, 131.53, 129.68, 129.29, 128.67, 128.05, 126.60, 124.42, 122.54, 111.01 (t, J = 15.3), 18.95. HRMS (EI-TOF): m/z Calc. for $C_{22}H_{14}F_5NO$ [M]: 403.0996, found 403.0998.

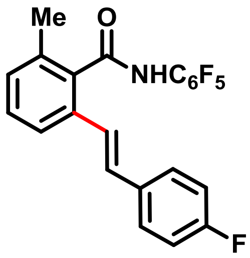

(E)-2-(4-Fluorostyryl)-6-methyl-N-(perfluorophenyl)benzamide (**5b**)

Following the general procedure, the C–H olefination was carried out with **1a** (60.2 mg, 0.2 mmol), $[RhCp^*Cl_2]_2$ (6.2 mg, 0.01 mmol), NaOPiv (28.4 mg, 0.2 mmol), MeCN (2.0 mL) and 4-fluorostyrene (61.0 mg, 0.5 mmol) at 80 °C for 24 hours. The product **5b** was obtained as white solid (53.9 mg, 64%) by flash column chromatography on silica gel using hexanes/EtOAc as the eluent. ^1H NMR (400 MHz, $CDCl_3$): δ 7.46 (d, J = 8.8 Hz, 1 H), 7.41 (dd, J_1 = 8.6 Hz, J_2 = 5.4 Hz, 2 H), 7.36 (s, 1 H), 7.30 (t, J = 8.0 Hz, 1 H), 7.12-7.08 (m, 2 H), 7.06-6.97 (m, 3 H), 2.34 (s, 3 H); ^{13}C NMR (100 MHz, $CDCl_3$): δ 168.52, 162.58 (d, J = 246.3 Hz), 143.36 (d, J = 247.2 Hz), 140.50 (d, J = 256.9 Hz), 137.80 (d, J = 253.5 Hz), 134.76 (d, J = 62.3 Hz), 134.36, 132.87, 130.38, 129.74, 129.36, 128.19, 128.11, 124.22, 122.51, 115.66 (d, J = 21.7 Hz), 111.01 (t, J = 15.2 Hz), 18.99. HRMS (EI-TOF): m/z Calc. for $C_{22}H_{13}F_6NO$ [M]: 421.0901, found 421.0904.

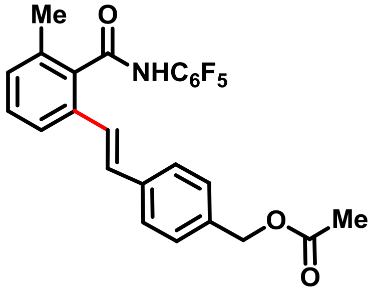

(E)-4-(3-Methyl-2-((perfluorophenyl)carbamoyl)styryl)benzyl acetate (**5c**)

Following the general procedure, the C–H olefination was carried out with **1a** (60.2 mg, 0.2 mmol), $[RhCp^*Cl_2]_2$ (6.2 mg, 0.01 mmol), NaOPiv (28.4 mg, 0.2 mmol), MeCN (2.0 mL) and 4-vinylbenzyl acetate (88.1 mg, 0.5 mmol) at 80 °C for 24 hours. The product **5c** was obtained as white solid (66.5 mg, 70%) by flash column chromatography on silica gel using hexanes/EtOAc as the eluent. ^1H NMR (400 MHz, $CDCl_3$): δ 7.60, 7.50 (d, J = 8.0 Hz, 1 H), 7.42 (d, J = 8.4 Hz, 1 H),

7.32-7.27 (m, 3 H), 7.19 (d, J = 16.0 Hz, 1 H), 7.10 (d, J = 7.6 Hz, 1 H), 7.01 (d, J = 16.0 Hz, 1 H), 5.06 (s, 2 H), 2.33 (s, 3 H), 2.08 (s, 3 H); ^{13}C NMR (100 MHz, $CDCl_3$): δ 170.99, 168.51, 143.33 (d, J = 246.3 Hz), 140.45 (d, J = 254.4 Hz), 137.62 (d, J = 246.7 Hz), 136.71, 135.65, 135.06, 134.62, 134.31, 130.87, 129.68, 129.42, 128.56, 126.73, 124.97, 122.54, 111.09 (t, J = 15.1), 65.94, 20.90, 18.97. HRMS (EI-TOF): *m/z* Calc. for $C_{25}H_{18}F_5NO_3$ [M]: 475.1207, found 475.1209.

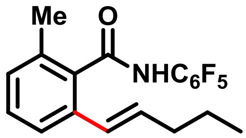

(E)-2-Methyl-6-(pent-1-en-1-yl)-N-(perfluorophenyl)benzamide (**5d**)

Following the general procedure, the C–H olefination was carried out with **1a** (60.2 mg, 0.2 mmol), $[RhCp^*Cl_2]_2$ (6.2 mg, 0.01 mmol), NaOPiv (28.4 mg, 0.2 mmol), MeCN (2.0 mL) and 1-pentene (36 mg, 0.5 mmol) at 80 °C for 24 hours. The product **5d** was obtained as white solid (32.5 mg, 44%) by flash column chromatography on silica gel using hexanes/EtOAc as the eluent. ^{1}H NMR (400 MHz, $CDCl_3$): δ 7.30 (t, J = 7.8 Hz, 1 H), 7.19 (d, J = 7.6 Hz, 1 H), 7.10 (d, J = 7.6 Hz, 1 H), 7.00 (s, 1 H), 5.22 (d, J = 1.2 Hz, 1 H), 5.10 (s, 1 H), 2.44 (s, 3 H), 2.38 (t, J = 7.8 Hz, 2 H), 1.47-1.38 (m, 2 H), 0.91 (t, J = 7.4 Hz, 3 H); ^{13}C NMR (100 MHz, $CDCl_3$): δ 167.90, 149.10, 143.19 (d, J = 242.1 Hz), 140.93, 140.31 (d, J = 240.4 Hz), 137.78 (d, J = 241.7 Hz), 135.82, 133.57, 129.57, 129.40, 126.24, 115.48, 111.32 (t, J = 14.8 Hz), 39.74, 21.19, 19.58, 13.74. HRMS (EI-TOF): *m/z* Calc. for $C_{19}H_{16}F_5NO$ [M]: 369.1152, found 369.1143.

NMR Spectra

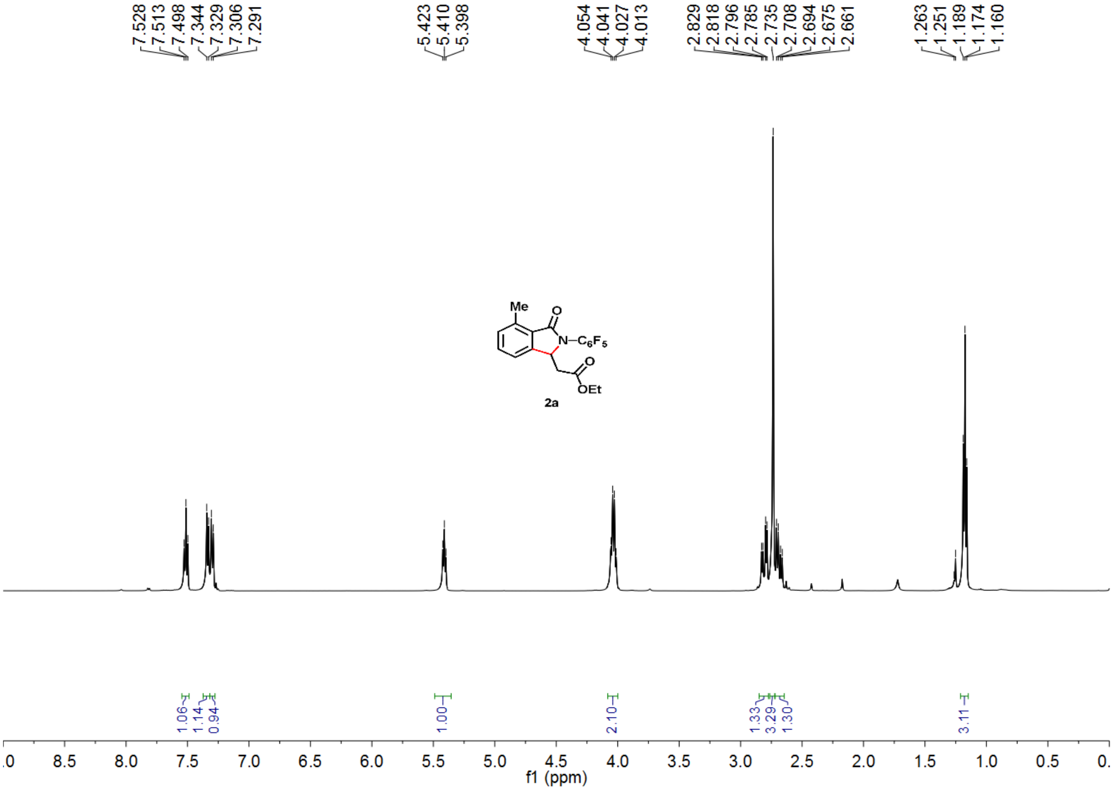
7.528
7.513
7.498
7.344
7.329
7.306
7.291
5.423
5.410
5.398
4.054
4.041
4.027
4.013
2.829
2.818
2.796
2.785
2.735
2.708
2.694
2.675
2.661
1.263
1.251
1.189
1.174
1.160
Me
O
N—C6F5
O
OEt
2a
1.06
1.14
0.94
1.00
2.10
1.33
3.29
1.30
3.11
f1 (ppm)

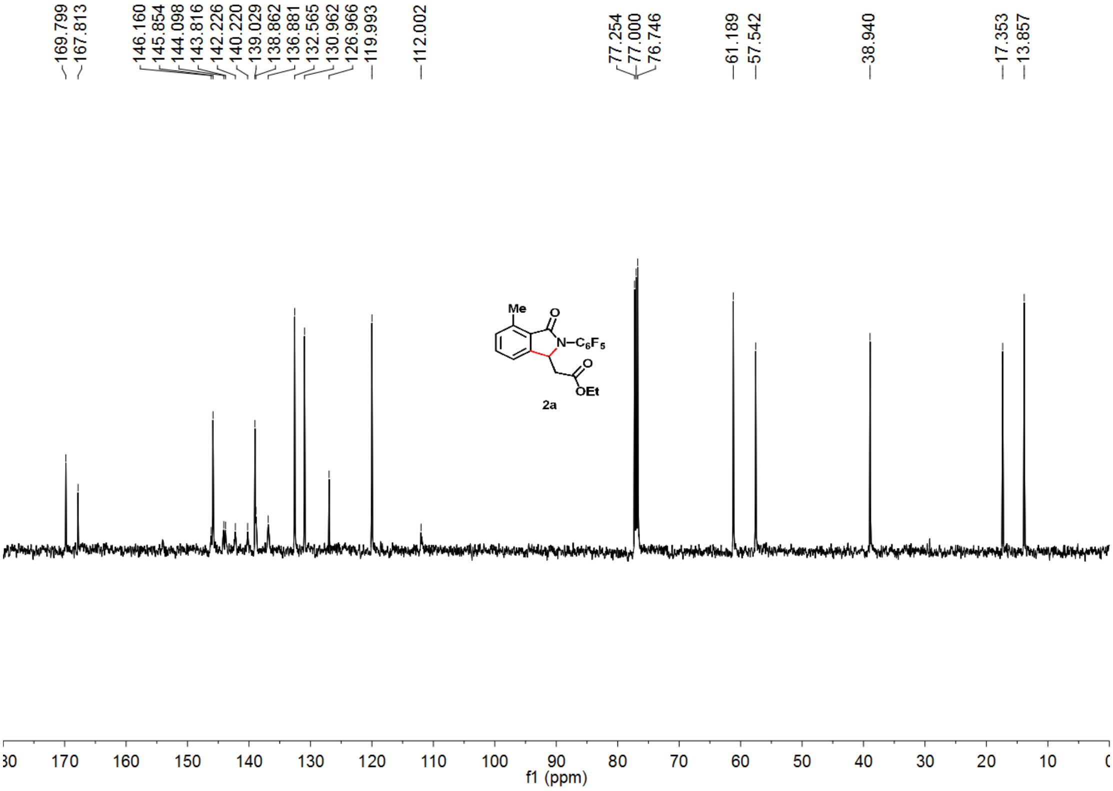
169.799
167.813
146.160
145.854
144.098
143.816
142.226
140.220
139.029
138.862
136.881
132.565
130.962
126.966
119.993
112.002
77.254
77.000
76.746
61.189
57.542
38.940
17.353
13.857
Me
O
N—C6F5
O
OEt
2a
f1 (ppm)

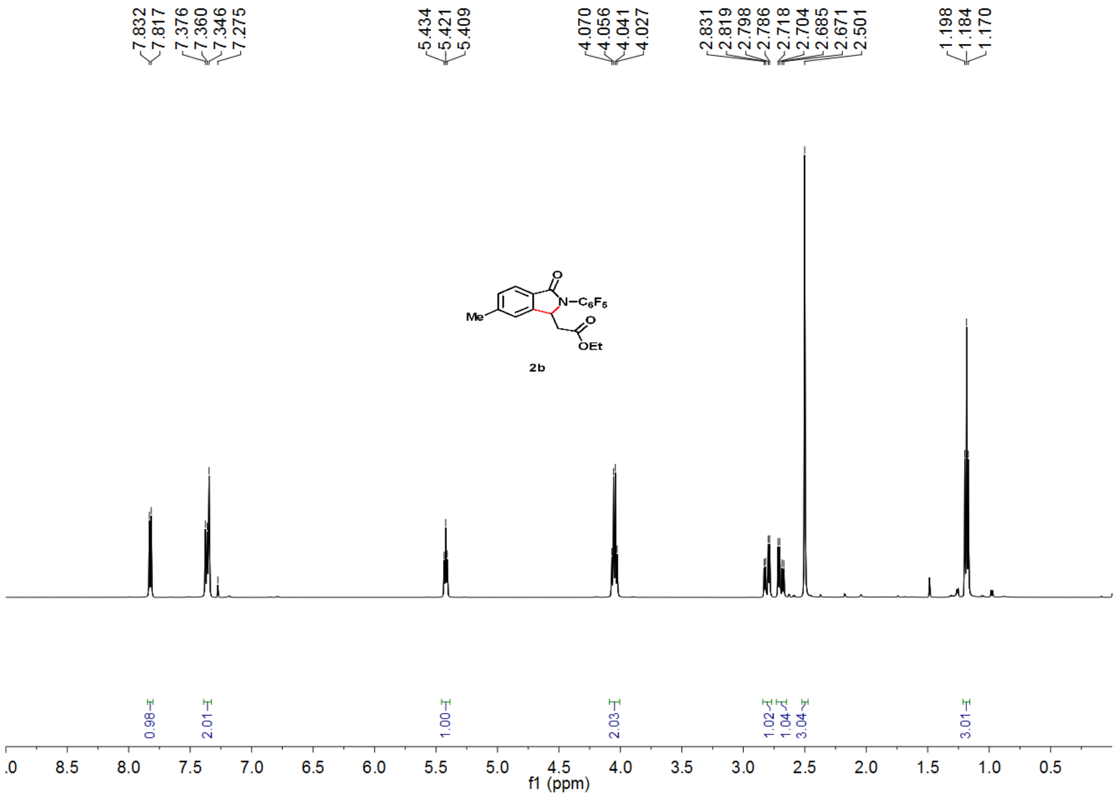
7.832
7.817
7.376
7.360
7.346
7.275
5.434
5.421
5.409
4.070
4.056
4.041
4.027
2.831
2.819
2.798
2.786
2.718
2.704
2.685
2.671
2.501
1.198
1.184
1.170
Me
O
N
C6F5
O
OEt
2b
0.98
2.01
1.00
2.03
1.02
1.04
3.04
3.01
f1 (ppm)

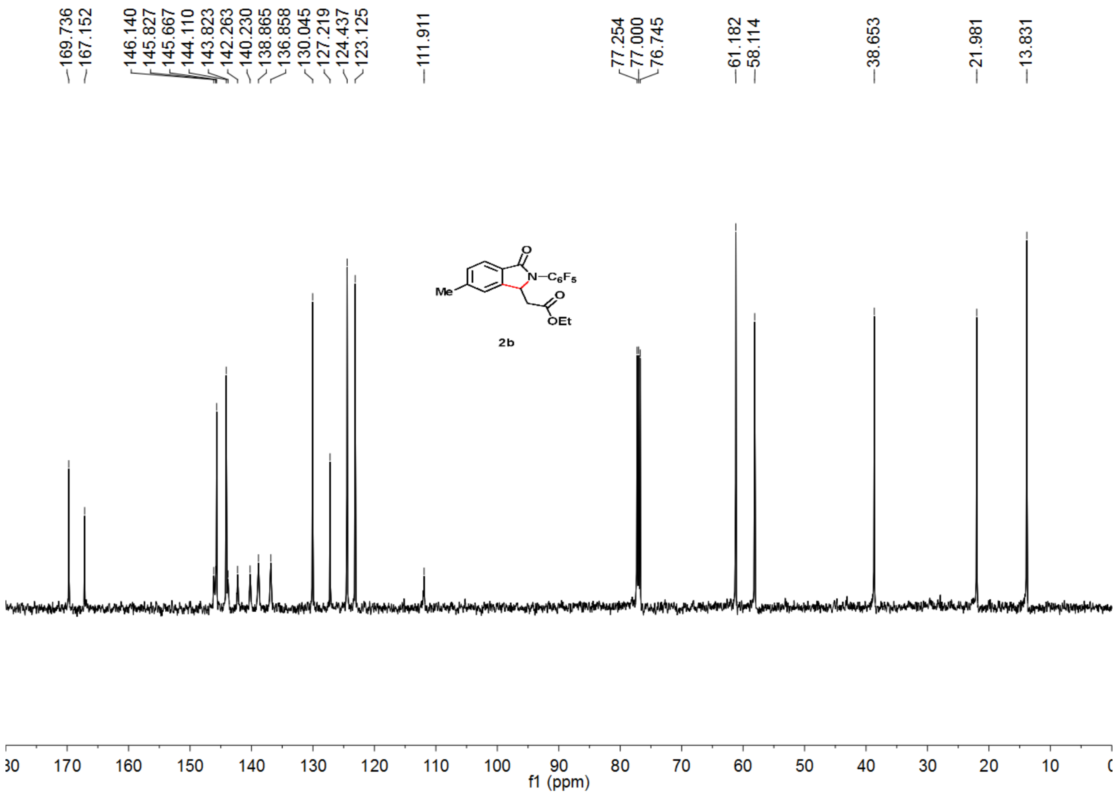
169.736
167.152
146.140
145.827
145.667
144.110
143.823
142.263
140.230
138.865
136.858
130.045
127.219
124.437
123.125
111.911
77.254
77.000
76.745
61.182
58.114
38.653
21.981
13.831
Me
O
N
C6F5
O
OEt
2b
f1 (ppm)

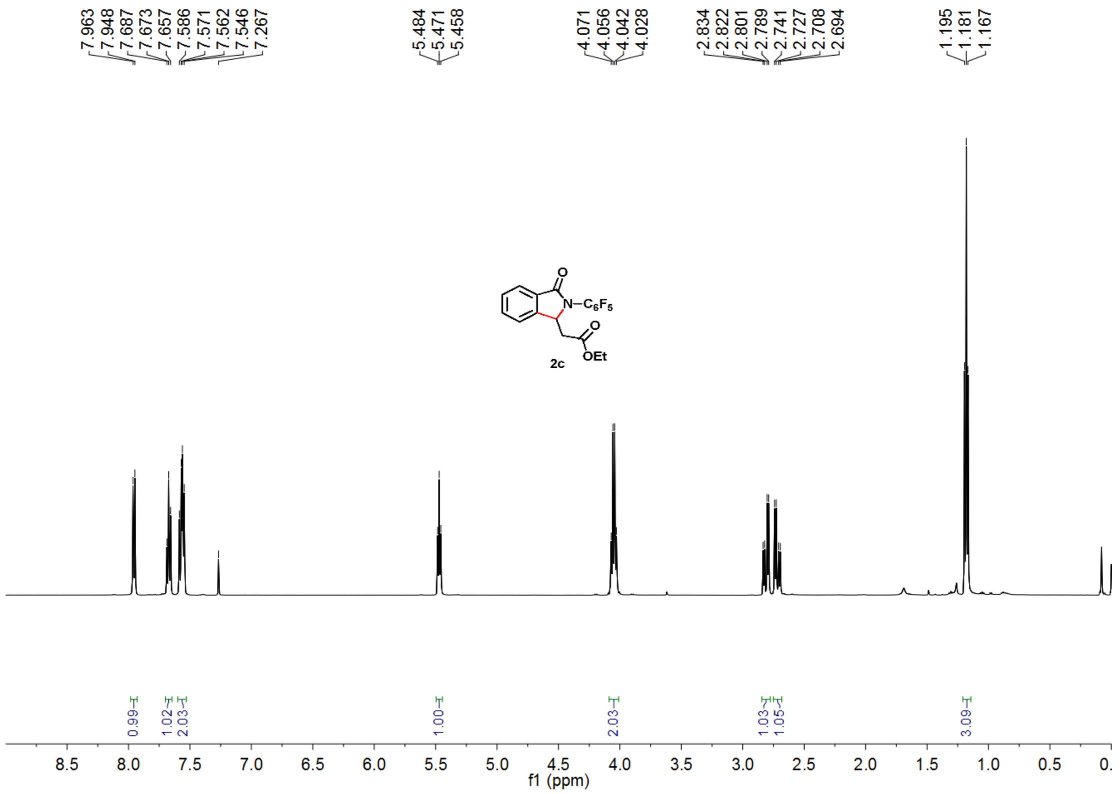
7.963
7.948
7.687
7.673
7.657
7.586
7.571
7.562
7.546
7.267
5.484
5.471
5.458
4.071
4.056
4.042
4.028
2.834
2.822
2.801
2.789
2.741
2.727
2.708
2.694
1.195
1.181
1.167
O
N−C6F5
O
OEt
2c
0.99
1.02
2.03
1.00
2.03
1.03
1.05
3.09
8.5 8.0 7.5 7.0 6.5 6.0 5.5 5.0 4.5 4.0 3.5 3.0 2.5 2.0 1.5 1.0 0.5 0.
f1 (ppm)

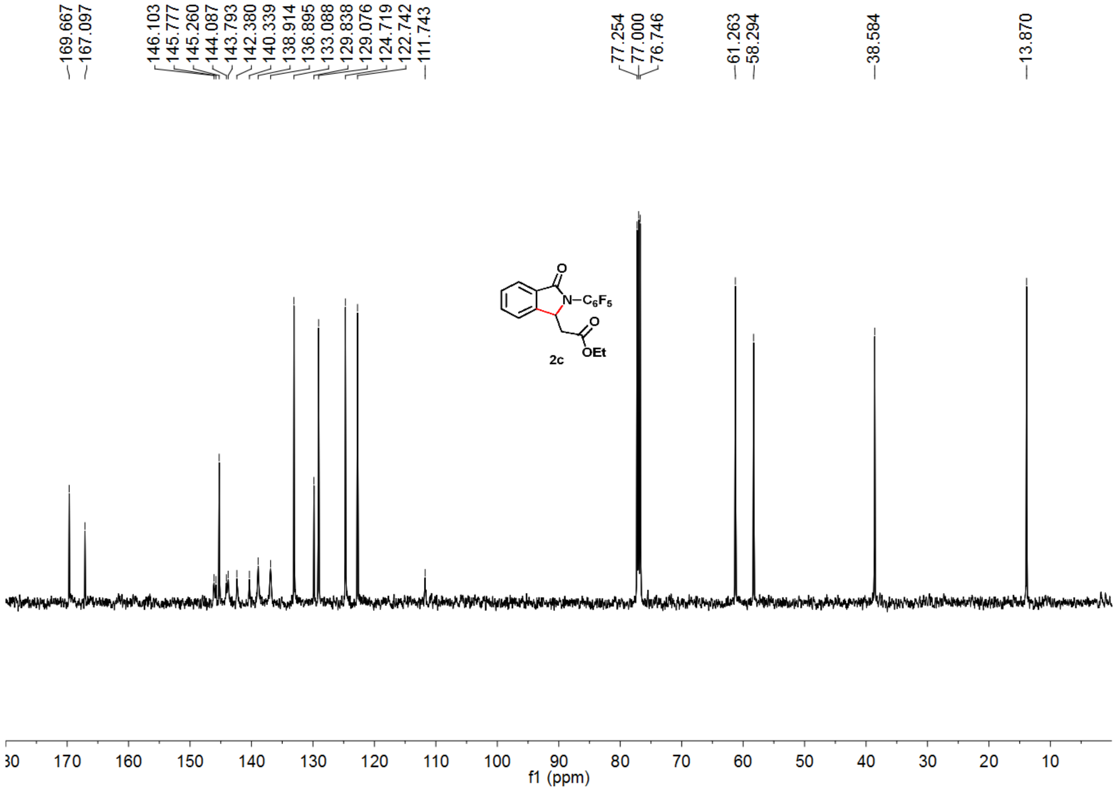
169.667
167.097
146.103
145.777
145.260
144.087
143.793
142.380
140.339
138.914
136.895
133.088
129.838
129.076
124.719
122.742
111.743
77.254
77.000
76.746
61.263
58.294
38.584
13.870
O
N−C6F5
O
OEt
2c
30 170 160 150 140 130 120 110 100 90 80 70 60 50 40 30 20 10
f1 (ppm)

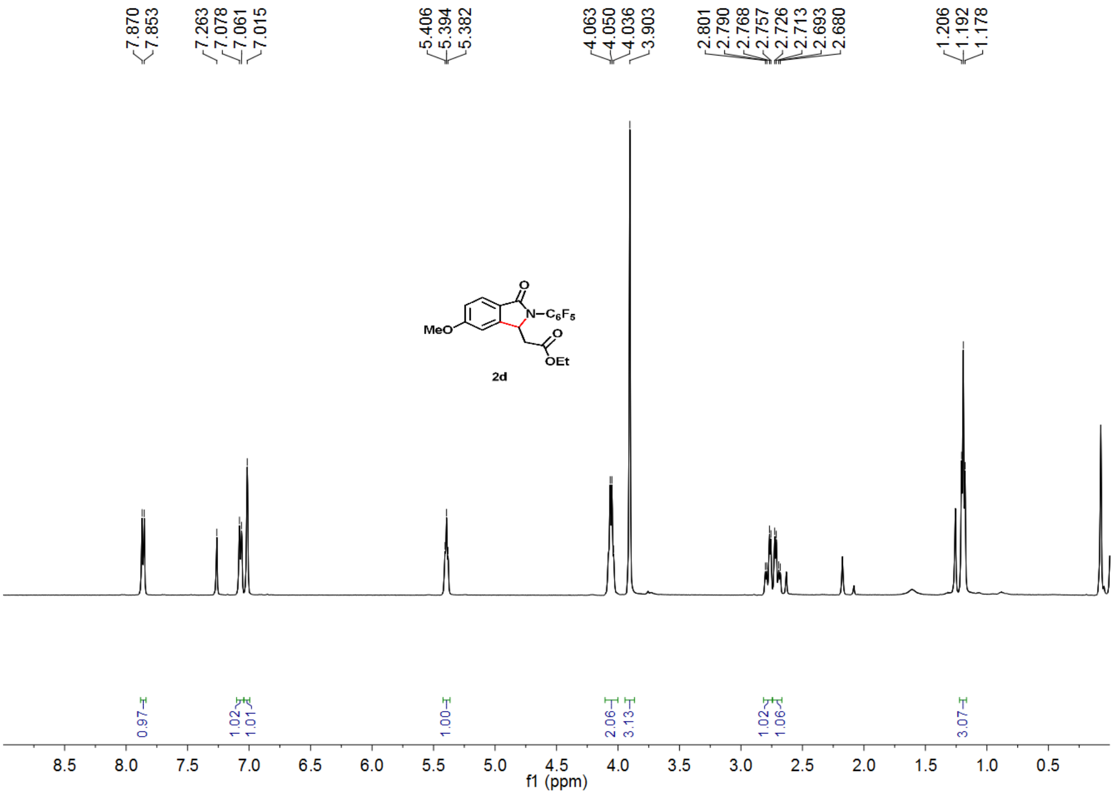
7.870
7.853
7.263
7.078
7.061
7.015
5.406
5.394
5.382
4.063
4.050
4.036
3.903
2.801
2.790
2.768
2.757
2.726
2.713
2.693
2.680
1.206
1.192
1.178
MeO
N−C6F5
OEt
2d
0.97
1.02
1.01
1.00
2.06
3.13
1.02
1.06
3.07
f1 (ppm)

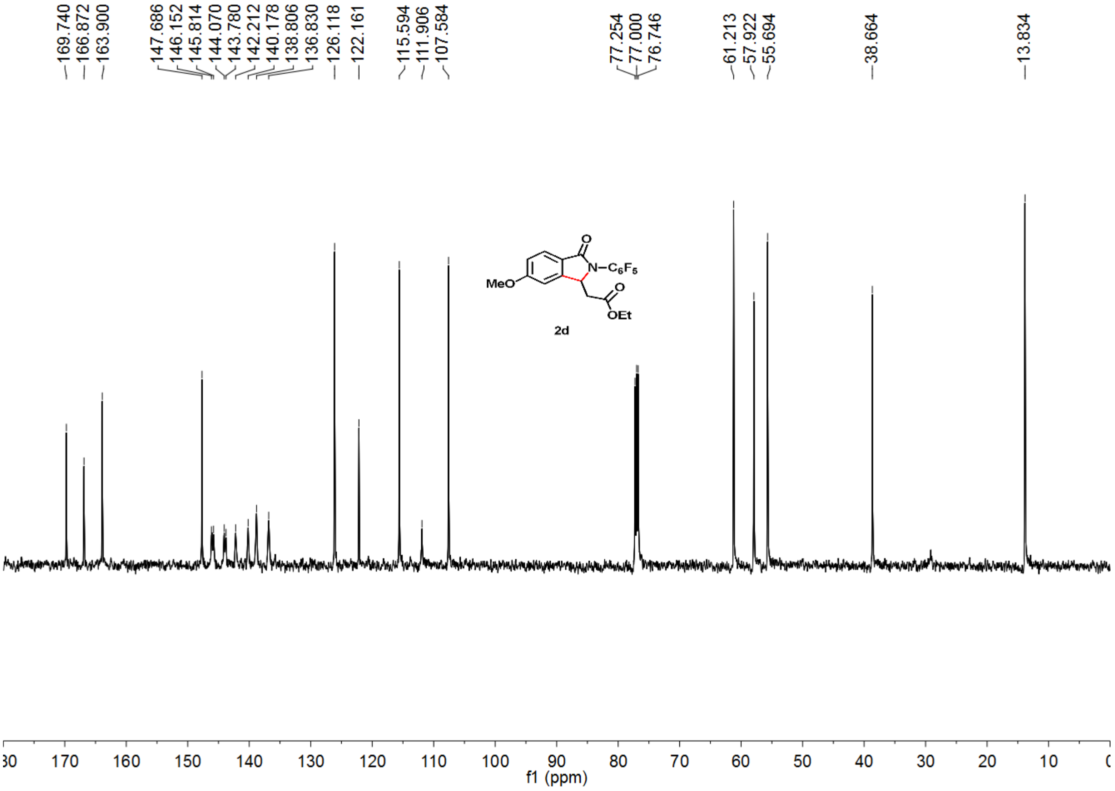
169.740
166.872
163.900
147.686
146.152
145.814
144.070
143.780
142.212
140.178
138.806
136.830
126.118
122.161
115.594
111.906
107.584
77.254
77.000
76.746
61.213
57.922
55.694
38.664
13.834
MeO
N−C6F5
OEt
2d
f1 (ppm)

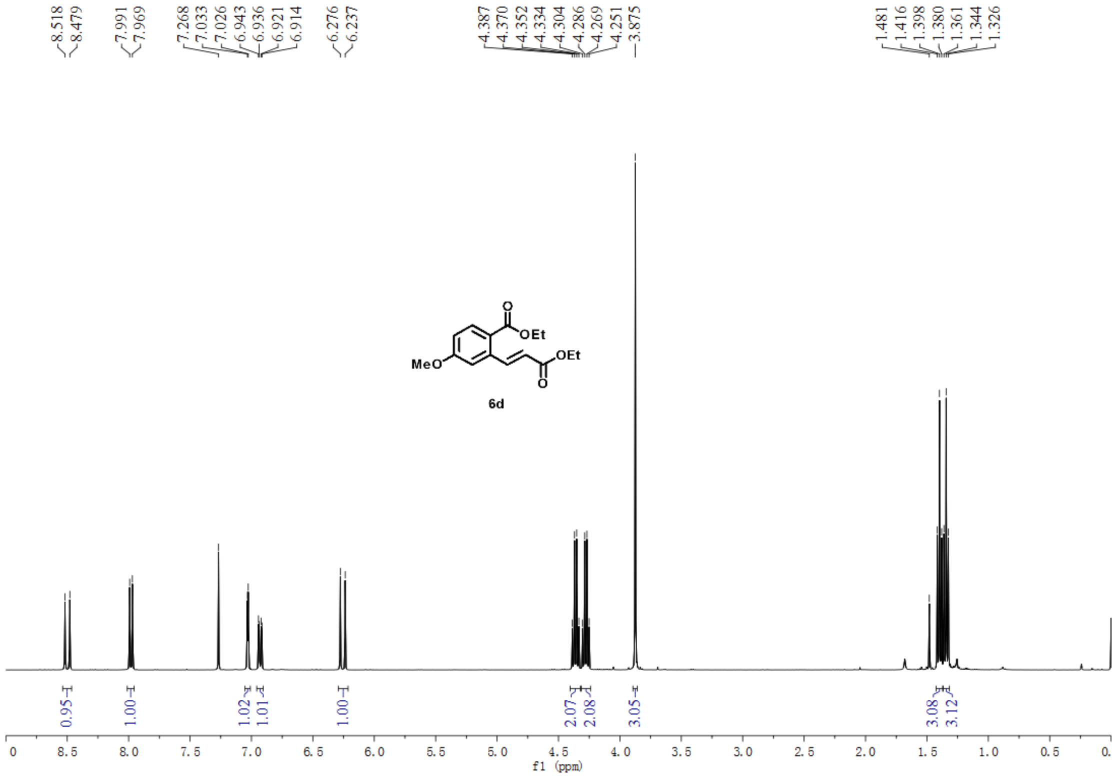
8.518
8.479
7.991
7.969
7.268
7.033
7.026
6.943
6.936
6.921
6.914
6.276
6.237
4.387
4.370
4.352
4.334
4.304
4.286
4.269
4.251
3.875
1.481
1.416
1.398
1.380
1.361
1.344
1.326
OEt
MeO
OEt
6d
0.95
1.00
1.02
1.01
1.00
2.07
2.08
3.05
3.08
3.12
f1 (ppm)

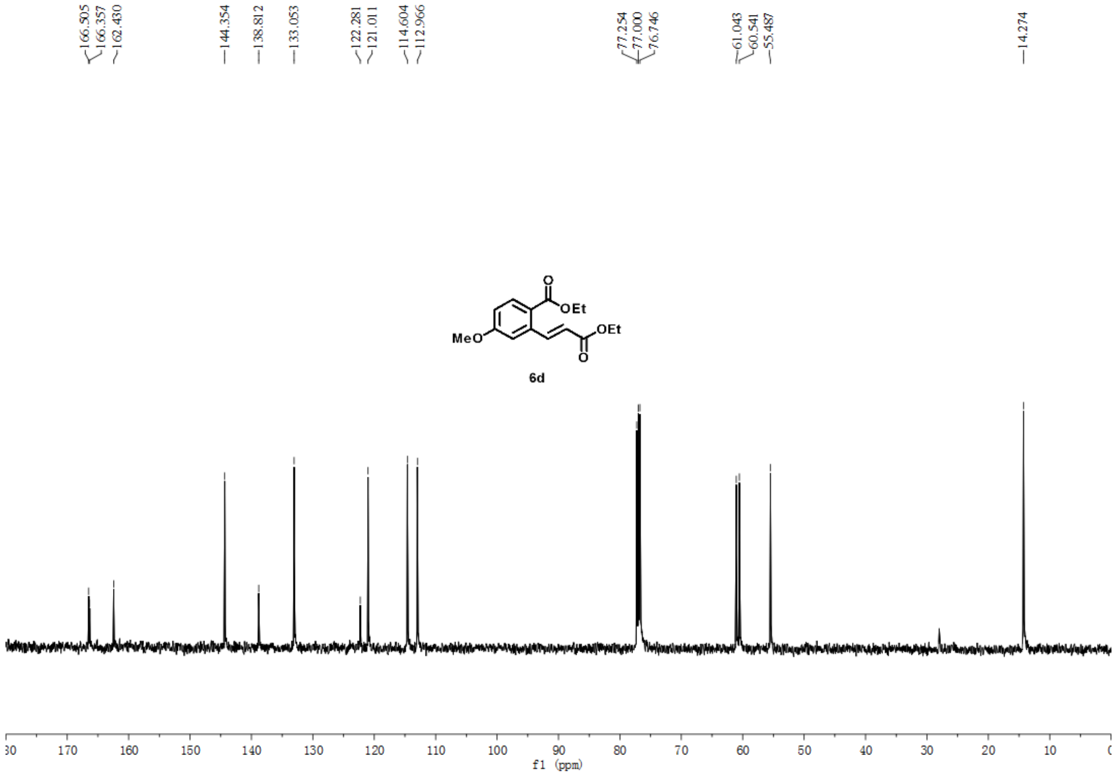
166.505
166.357
162.430
144.354
138.812
133.053
122.281
121.011
114.604
112.966
77.254
77.000
76.746
61.043
60.541
55.487
14.274
OEt
MeO
OEt
6d
f1 (ppm)

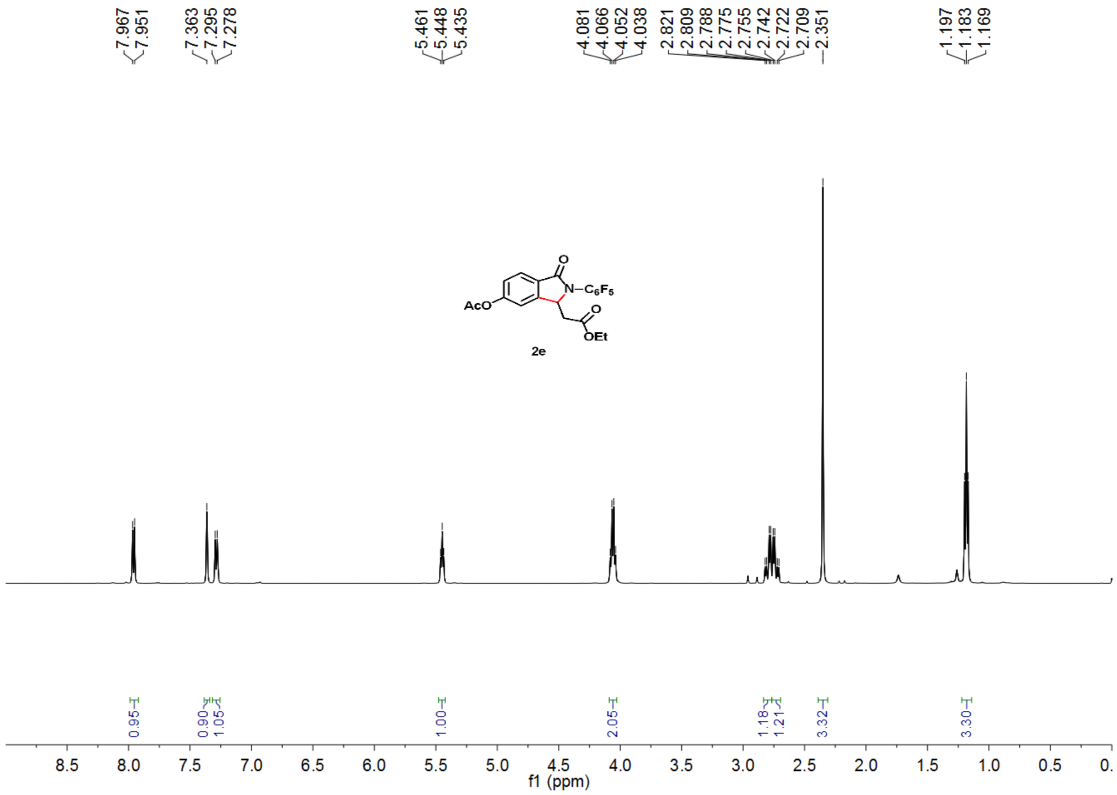
2e
AcO
N–C6F5
OEt
f1 (ppm)

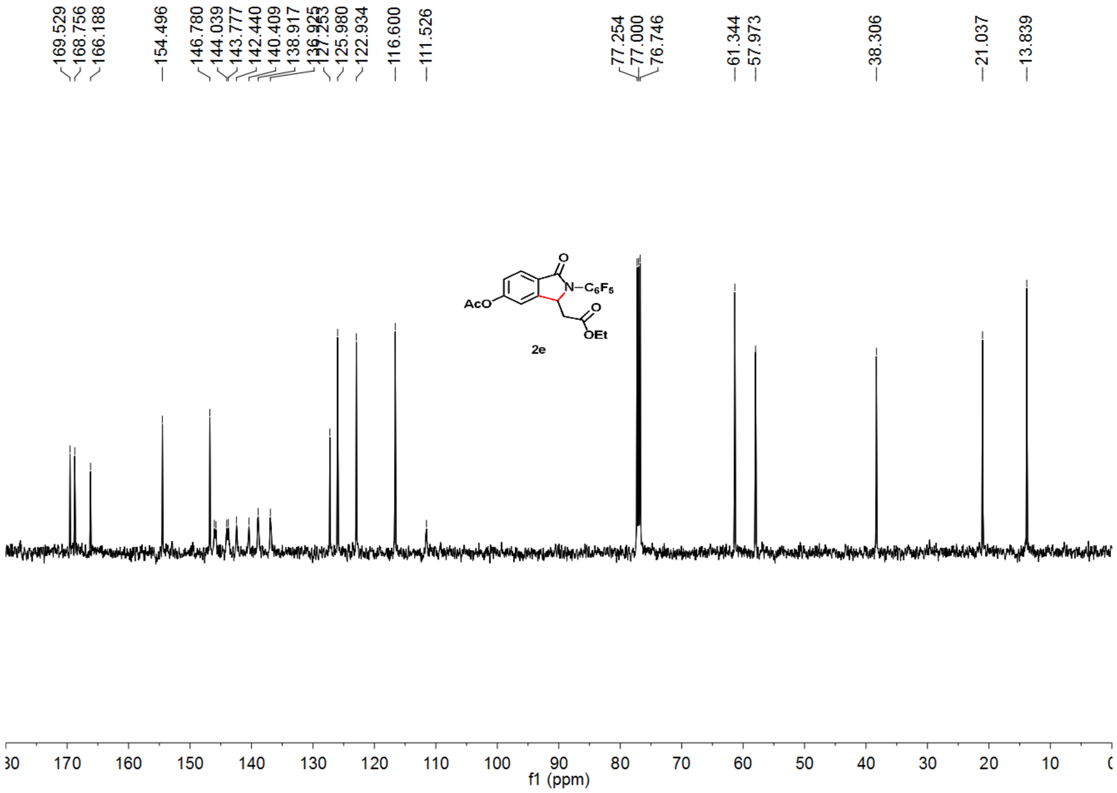
2e
AcO
N–C6F5
OEt
f1 (ppm)

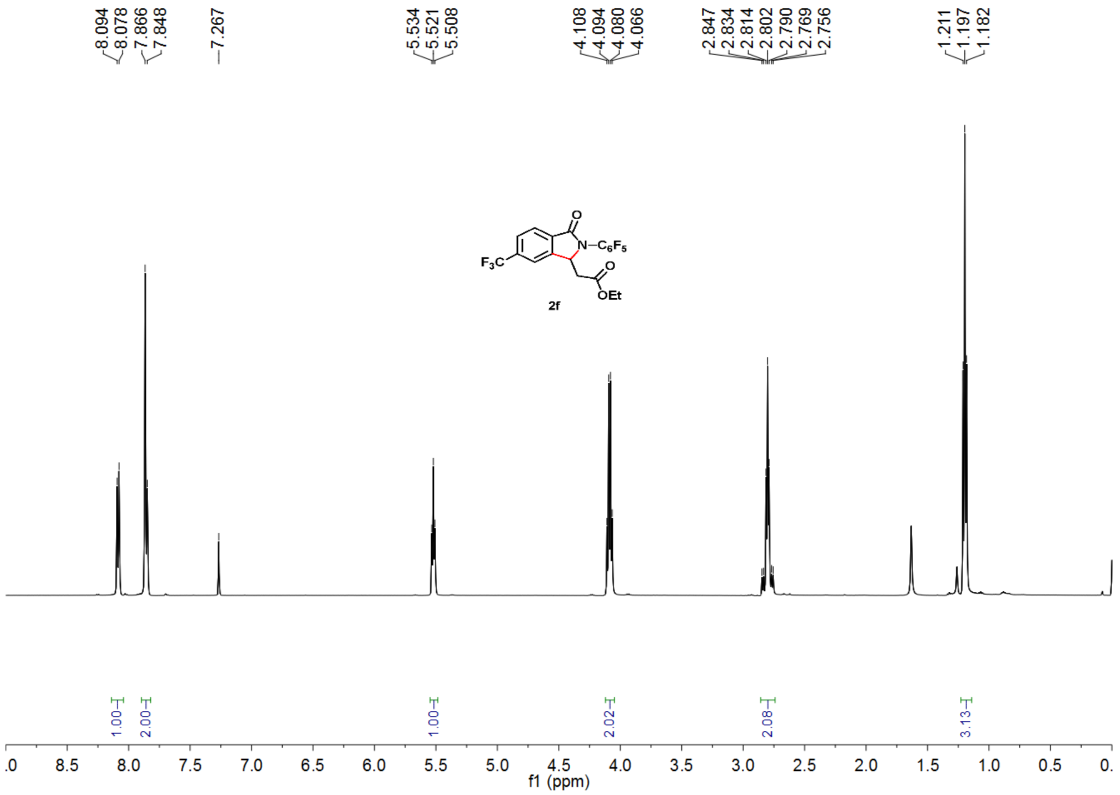

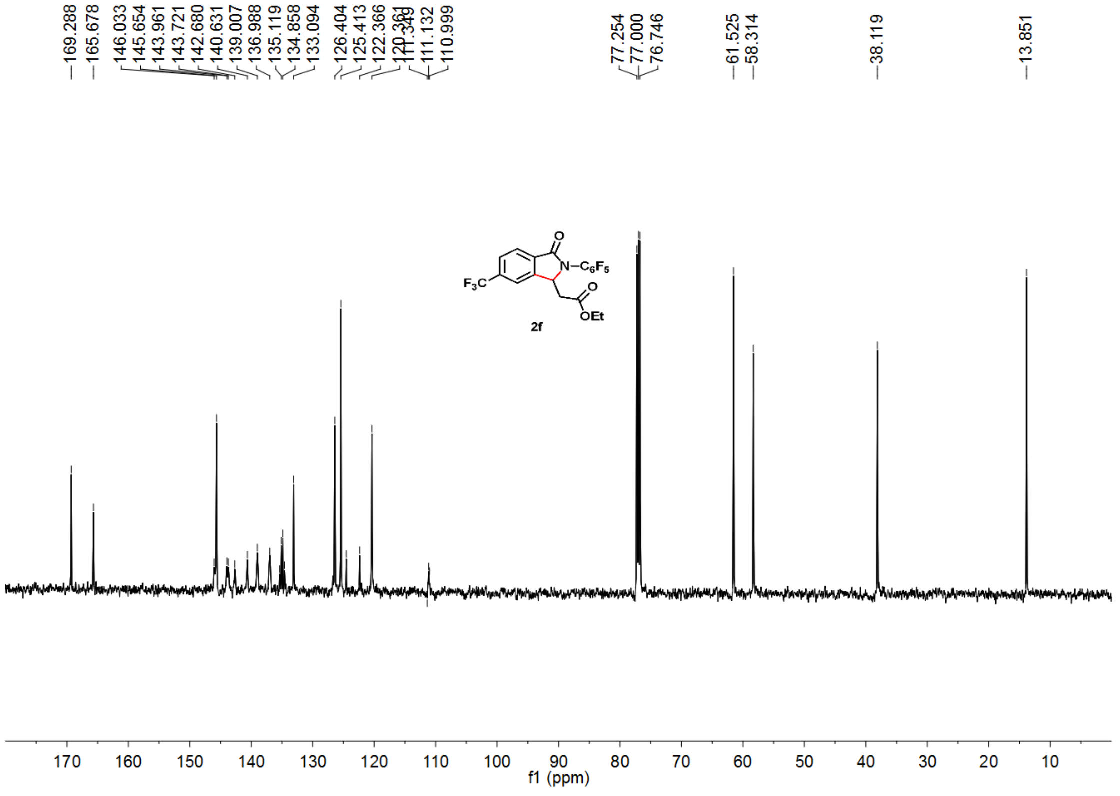

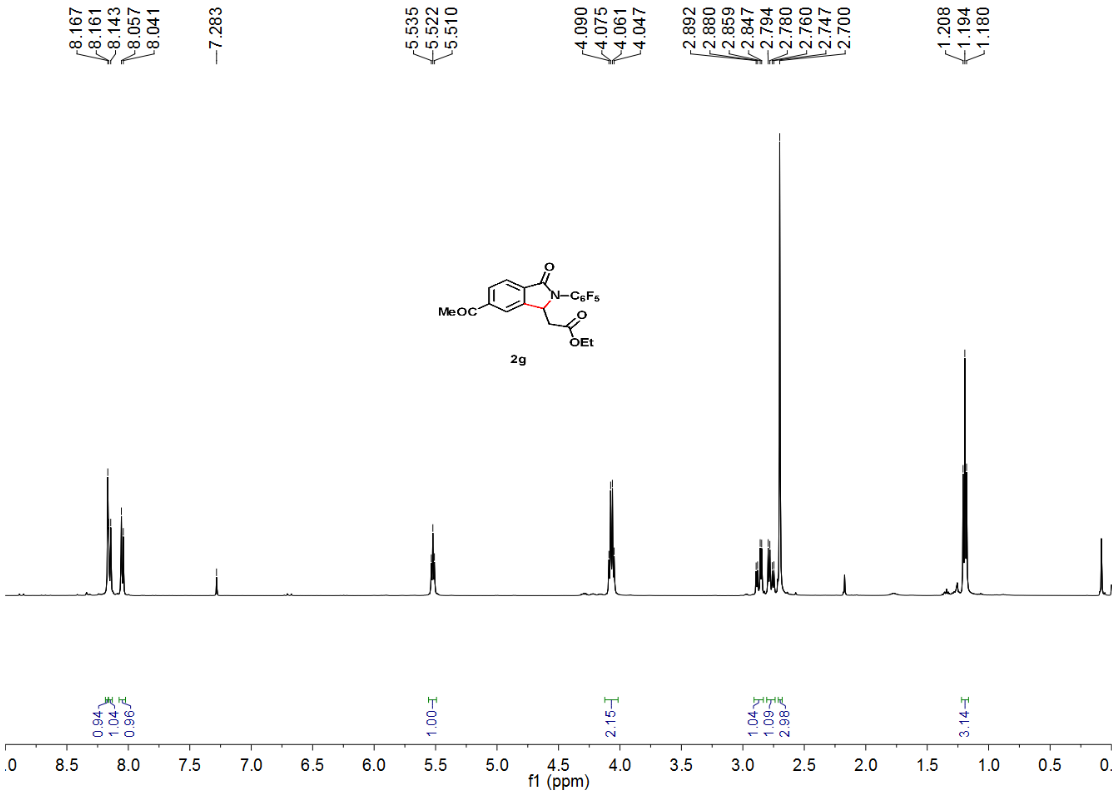

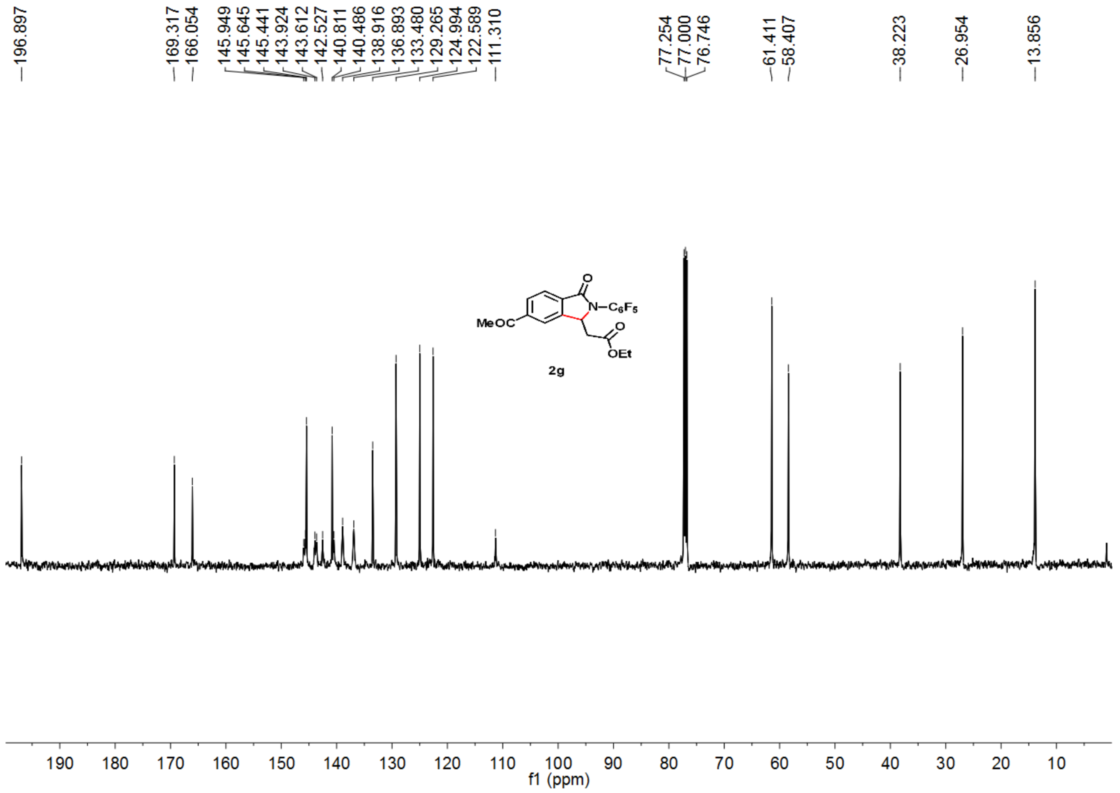

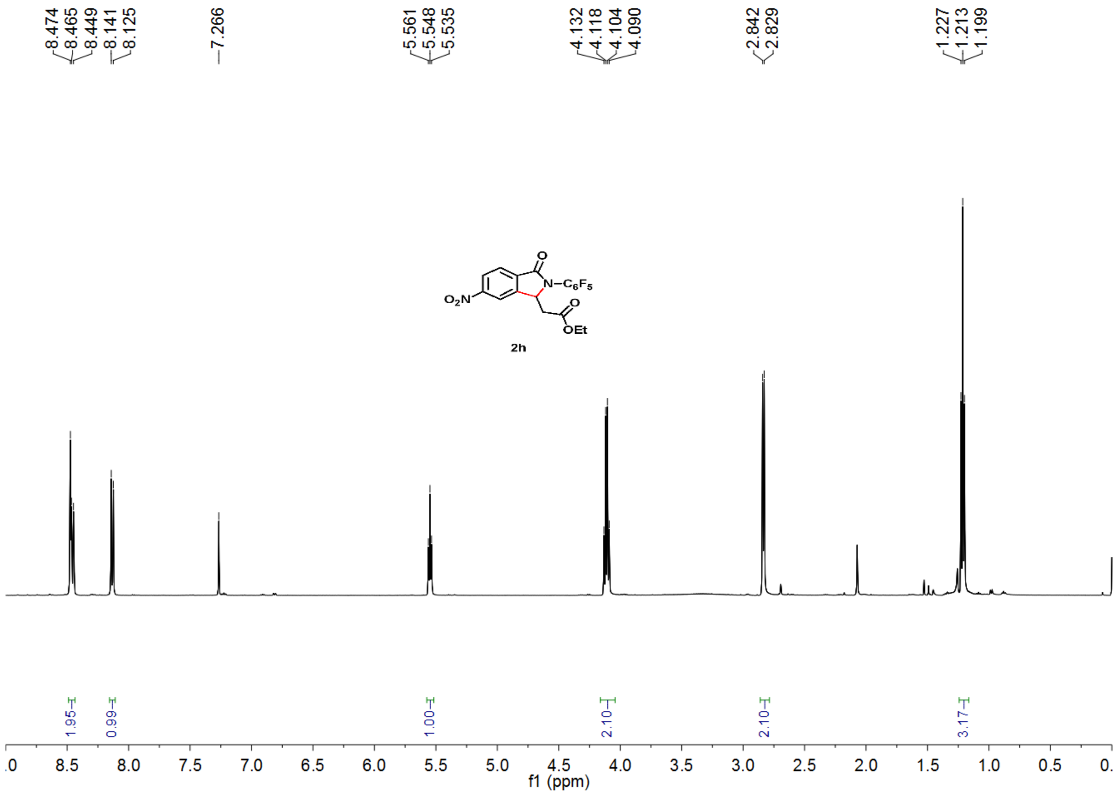
8.474
8.465
8.449
8.141
8.125
7.266
5.561
5.548
5.535
4.132
4.118
4.104
4.090
2.842
2.829
1.227
1.213
1.199
O
N–C6F5
O2N
O
OEt
2h
1.95
0.99
1.00
2.10
2.10
3.17
f1 (ppm)

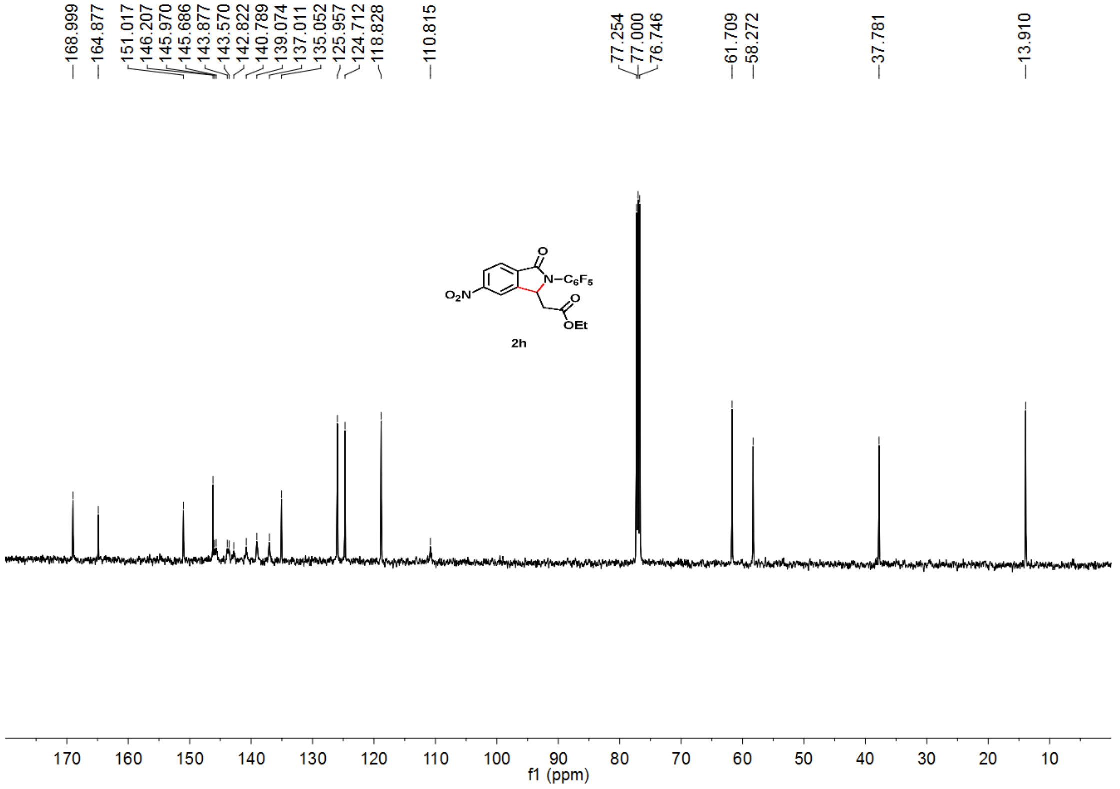
168.999
164.877
151.017
146.207
145.970
145.686
143.877
143.570
142.822
140.789
139.074
137.011
135.052
125.957
124.712
118.828
110.815
77.254
77.000
76.746
61.709
58.272
37.781
13.910
O
N–C6F5
O2N
O
OEt
2h
f1 (ppm)

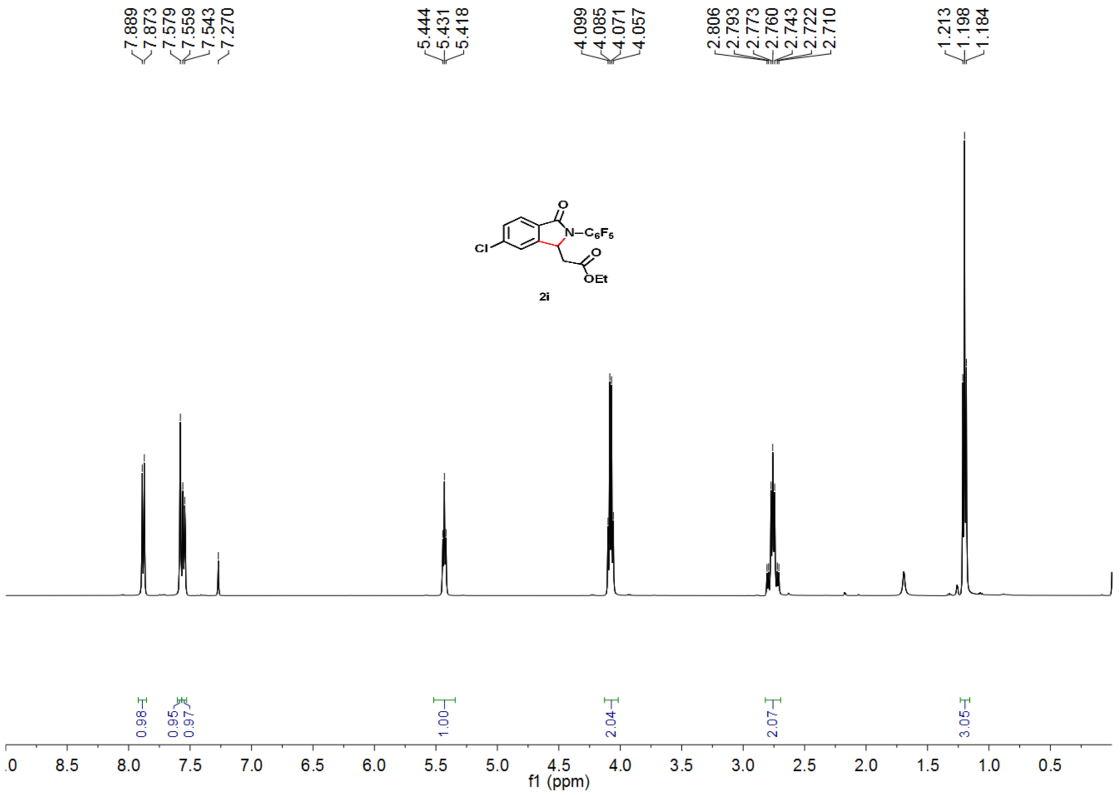

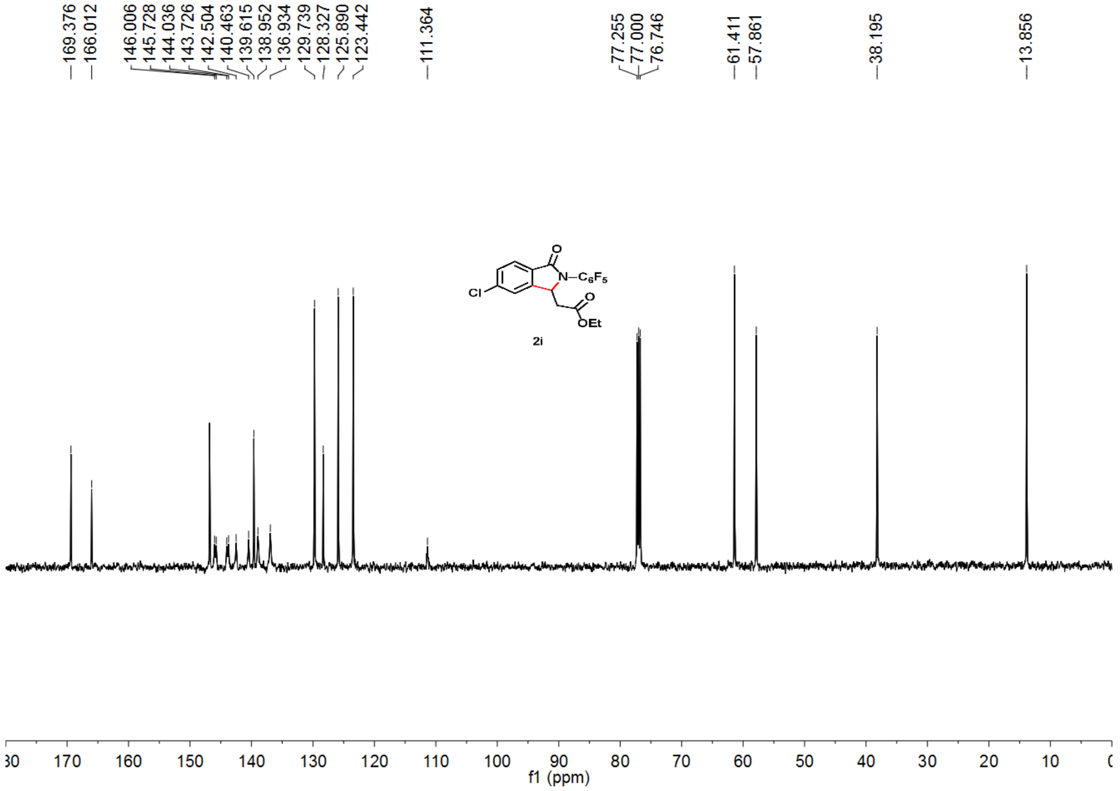

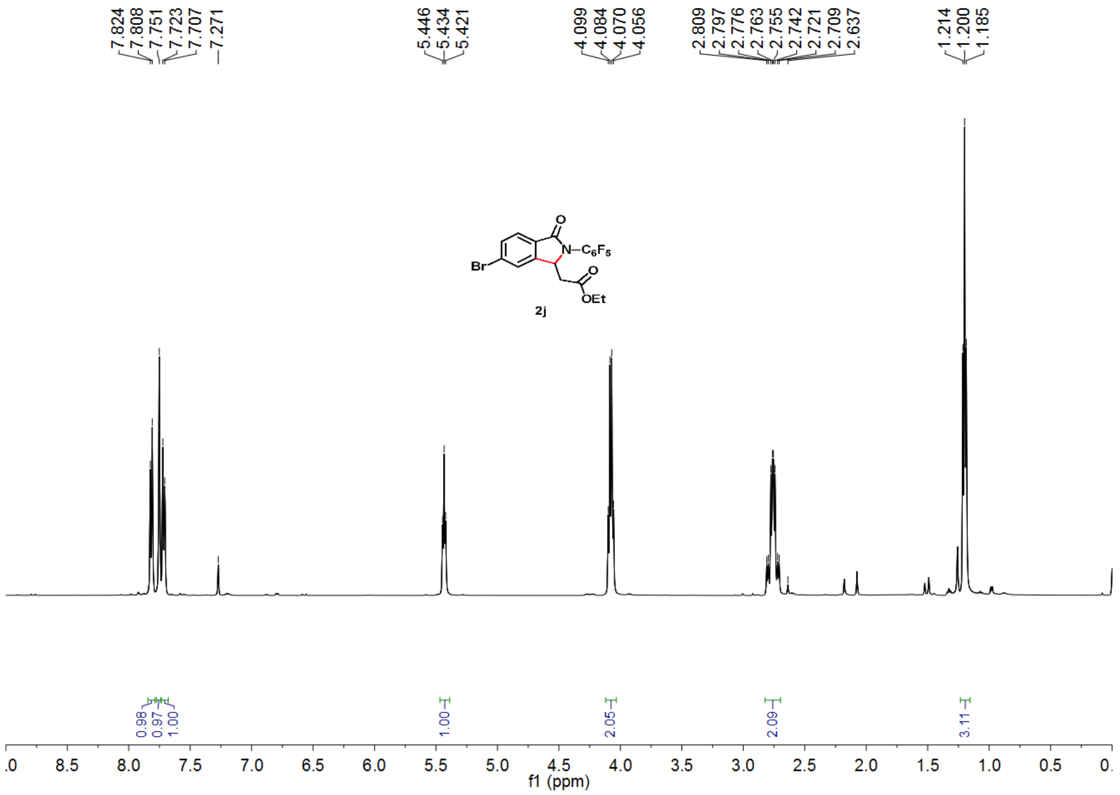
7.824
7.808
7.751
7.723
7.707
7.271
5.446
5.434
5.421
4.099
4.084
4.070
4.056
2.809
2.797
2.776
2.763
2.755
2.742
2.721
2.709
2.637
1.214
1.200
1.185
O
N–C6F5
Br
O
OEt
2j
0.98
0.97
1.00
1.00
2.05
2.09
3.11
8.5
8.0
7.5
7.0
6.5
6.0
5.5
5.0
4.5
4.0
3.5
3.0
2.5
2.0
1.5
1.0
0.5
f1 (ppm)

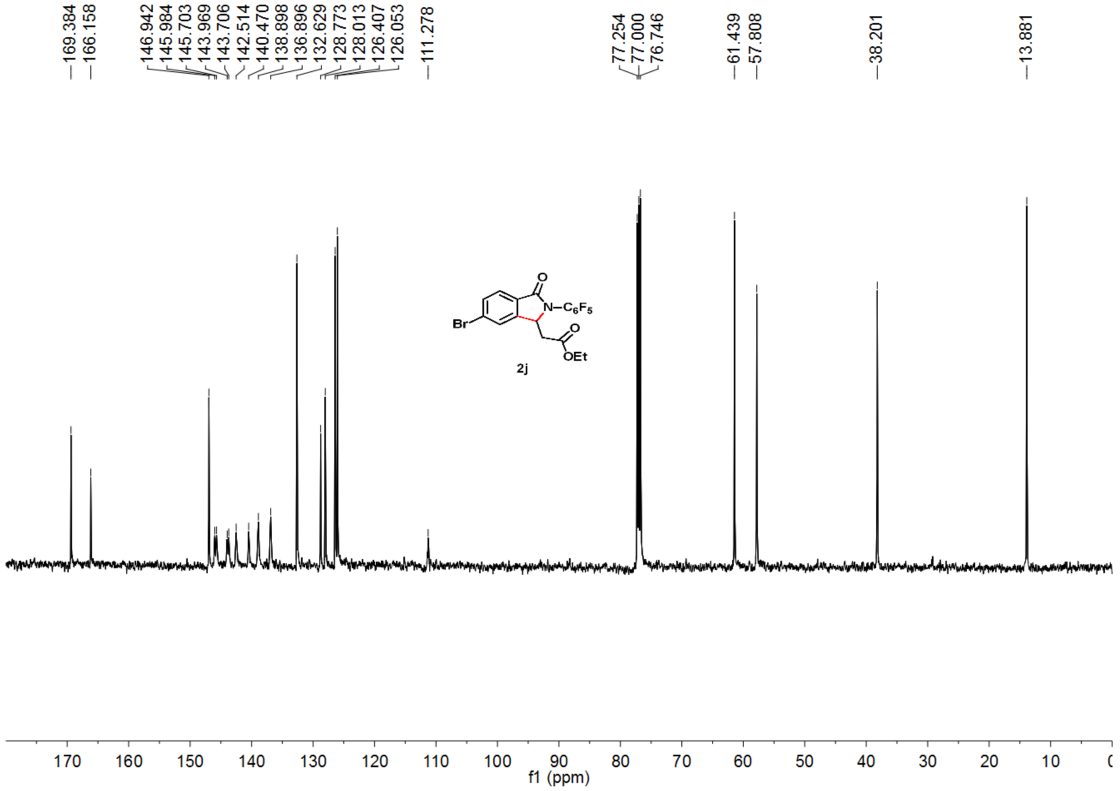
169.384
166.158
146.942
145.984
145.703
143.969
143.706
142.514
140.470
138.898
136.896
132.629
128.773
128.013
126.407
126.053
111.278
77.254
77.000
76.746
61.439
57.808
38.201
13.881
O
N–C6F5
Br
O
OEt
2j
170
160
150
140
130
120
110
100
90
80
70
60
50
40
30
20
10
f1 (ppm)

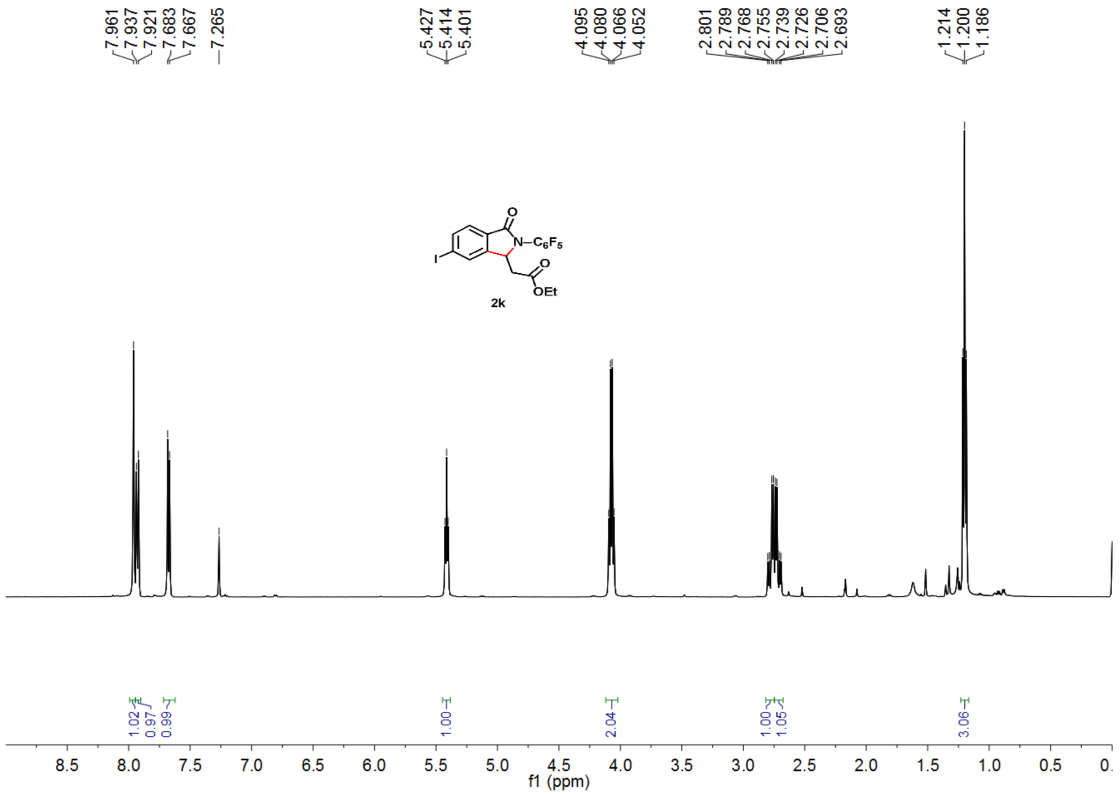
7.961
7.937
7.921
7.683
7.667
7.265
5.427
5.414
5.401
4.095
4.080
4.066
4.052
2.801
2.789
2.768
2.755
2.739
2.726
2.706
2.693
1.214
1.200
1.186
N–C6F5
I
O
OEt
2k
1.02
0.97
0.99
1.00
2.04
1.00
1.05
3.06
f1 (ppm)

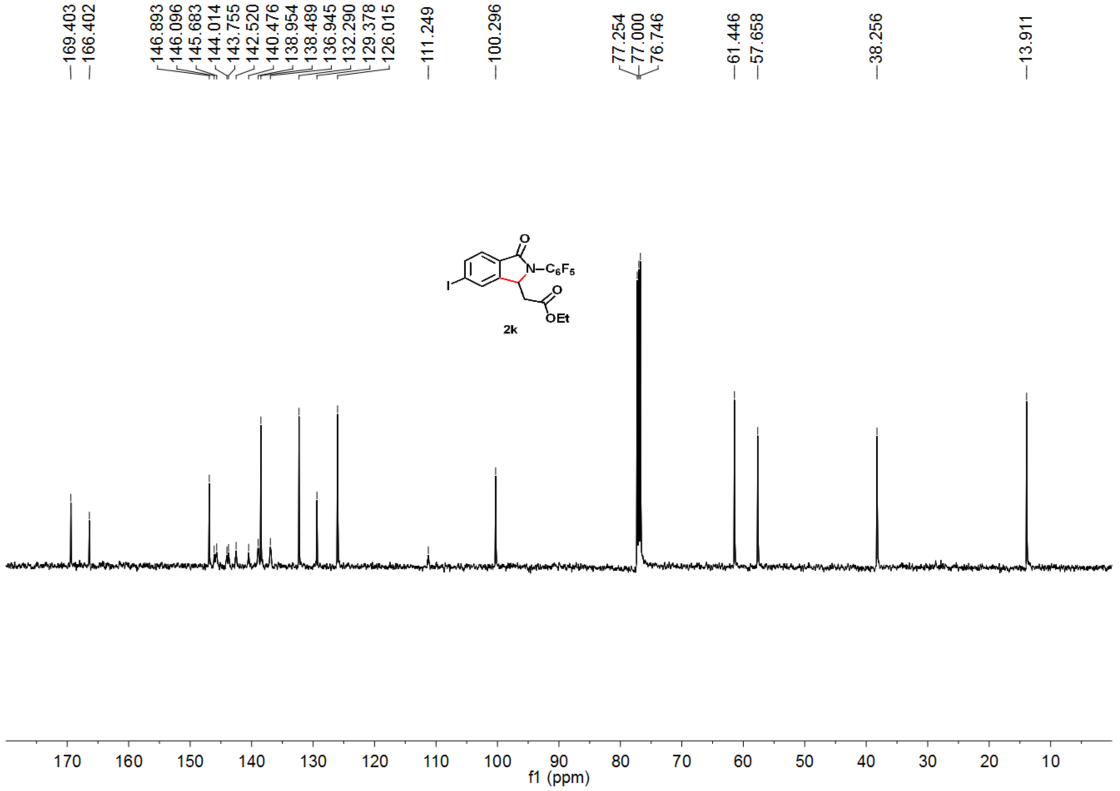
169.403
166.402
146.893
146.096
145.683
144.014
143.755
142.520
140.476
138.954
138.489
136.945
132.290
129.378
126.015
111.249
100.296
77.254
77.000
76.746
61.446
57.658
38.256
13.911
N–C6F5
I
O
OEt
2k
f1 (ppm)

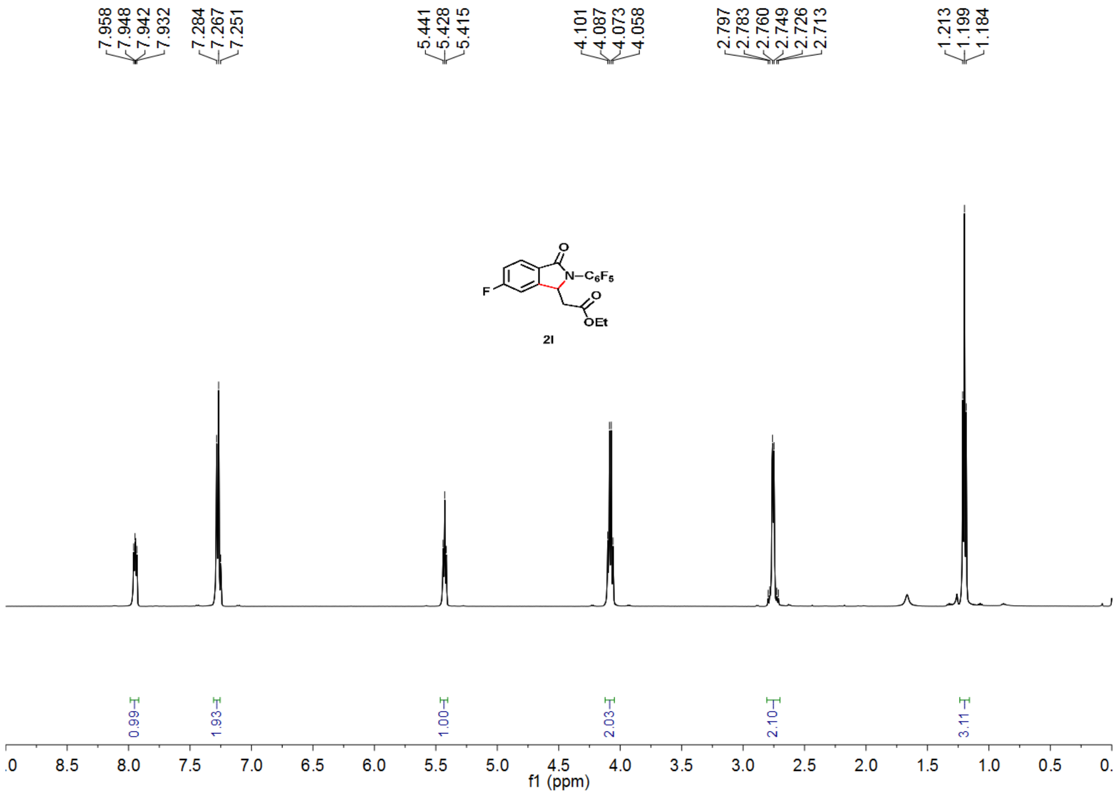
7.958
7.948
7.942
7.932
7.284
7.267
7.251
5.441
5.428
5.415
4.101
4.087
4.073
4.058
2.797
2.783
2.760
2.749
2.726
2.713
1.213
1.199
1.184
N–C6F5
F
O
OEt
2l
0.99
1.93
1.00
2.03
2.10
3.11
f1 (ppm)

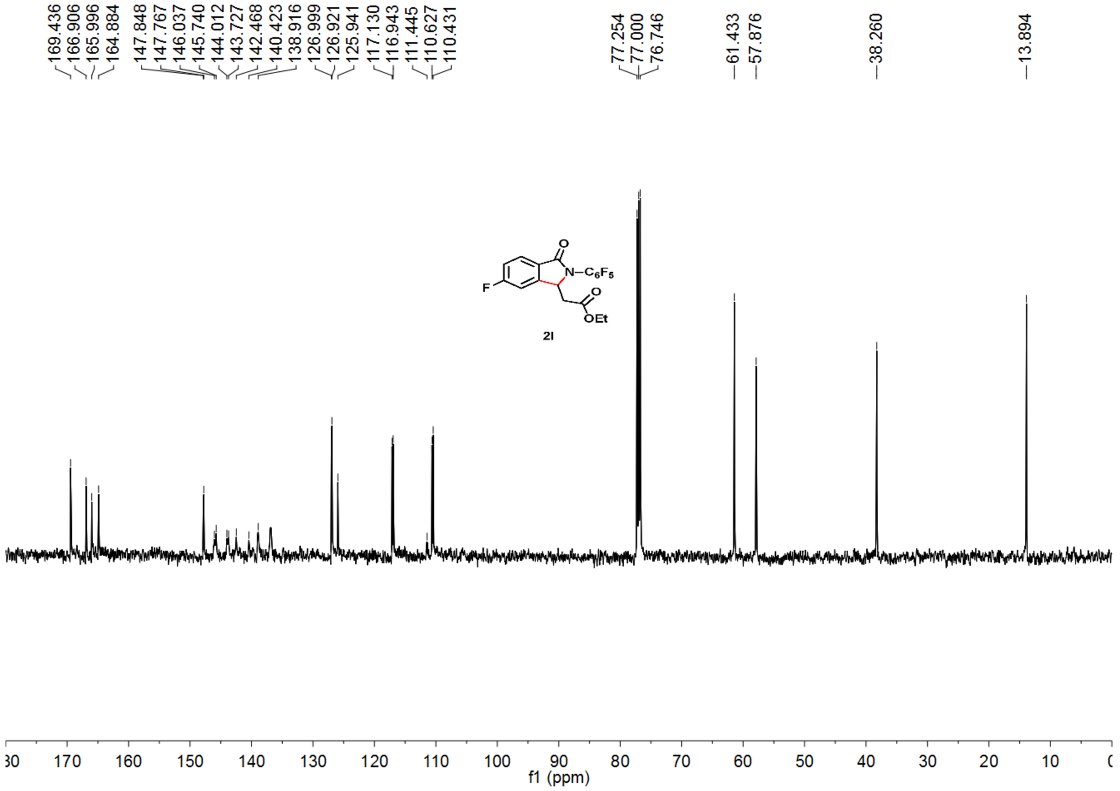
169.436
166.906
165.996
164.884
147.848
147.767
146.037
145.740
144.012
143.727
142.468
140.423
138.916
126.999
126.921
125.941
117.130
116.943
111.445
110.627
110.431
77.254
77.000
76.746
61.433
57.876
38.260
13.894
N–C6F5
F
O
OEt
2l
f1 (ppm)

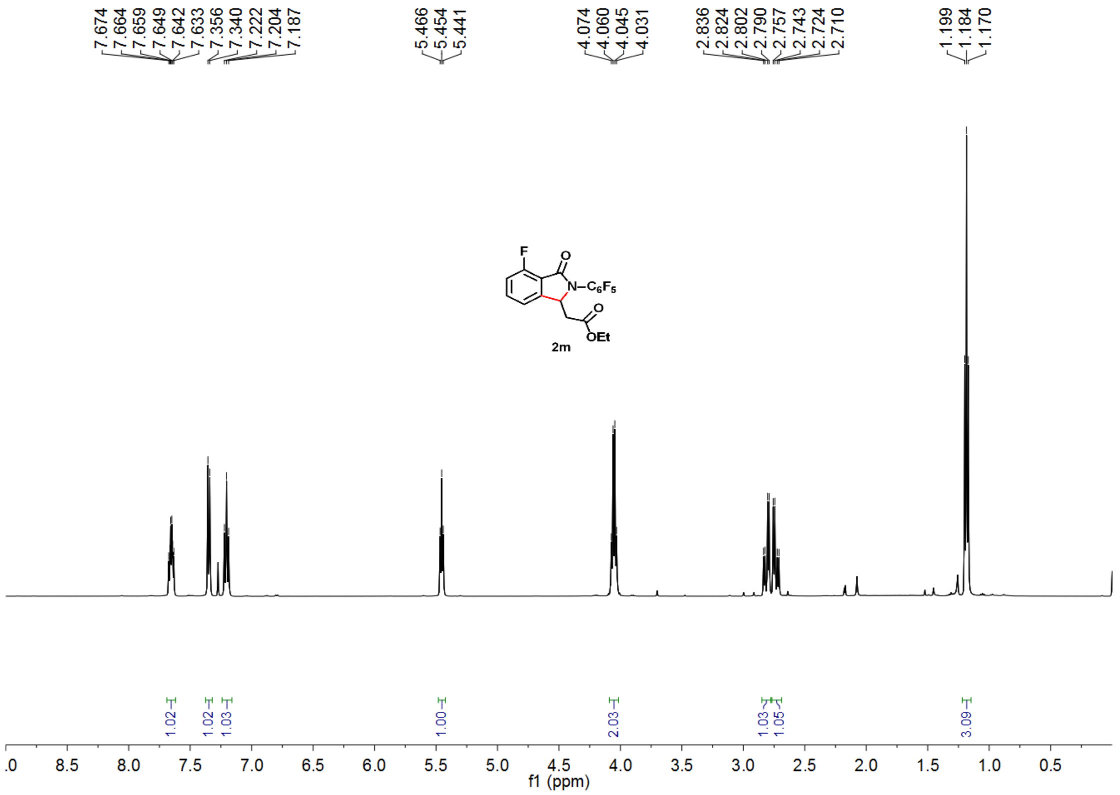

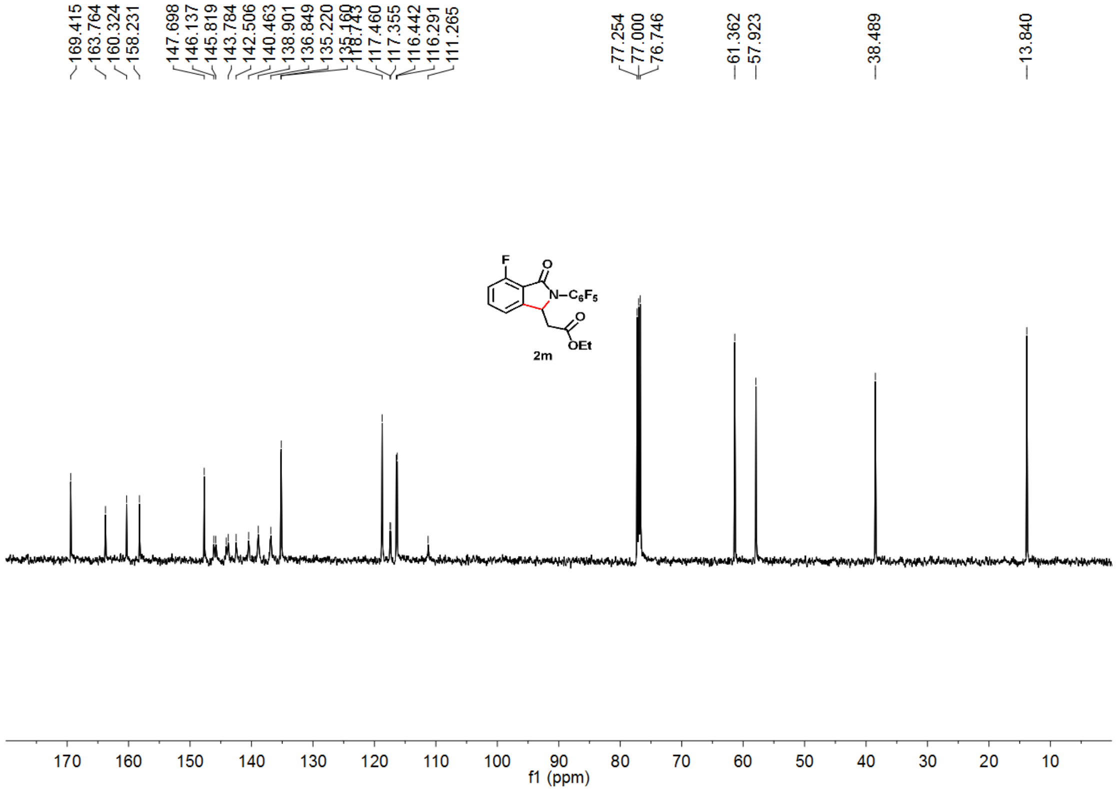

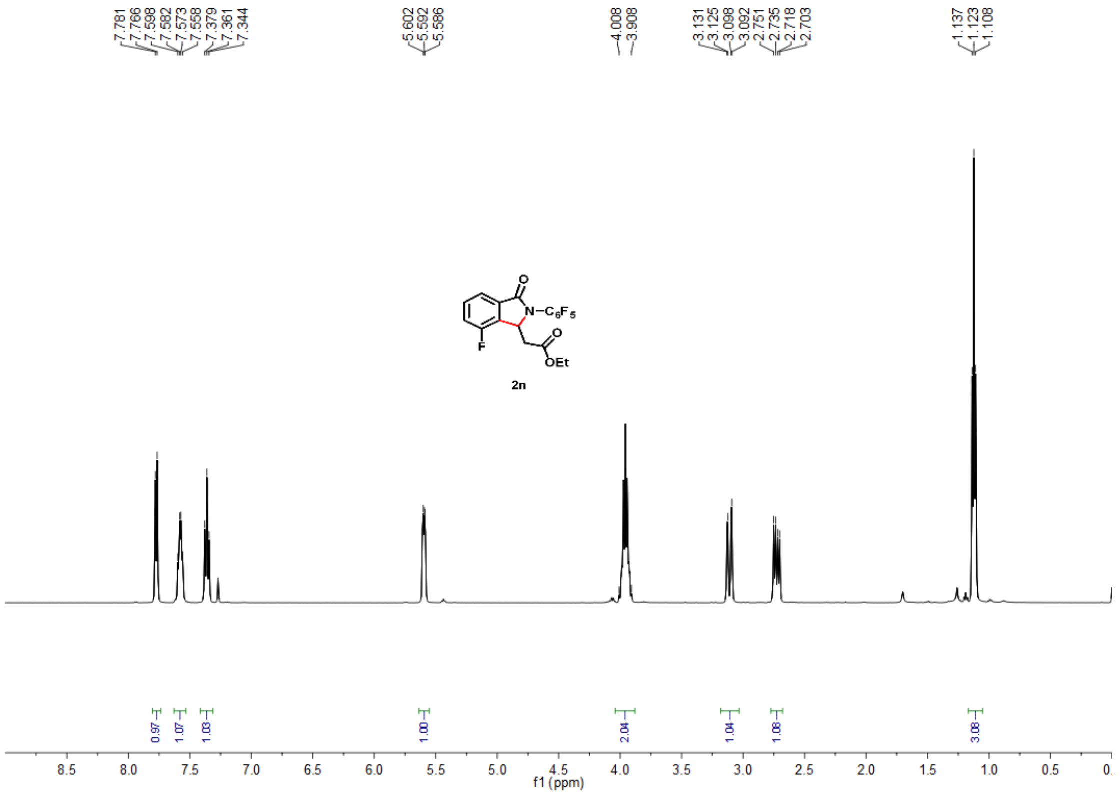

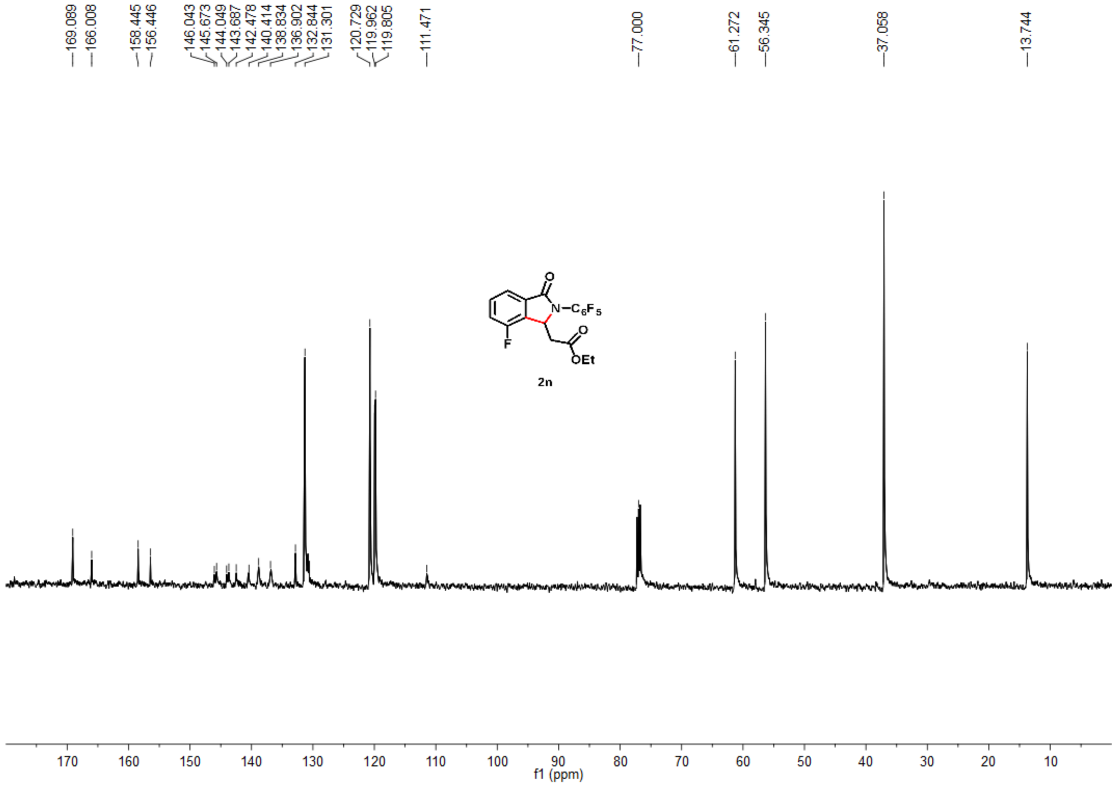

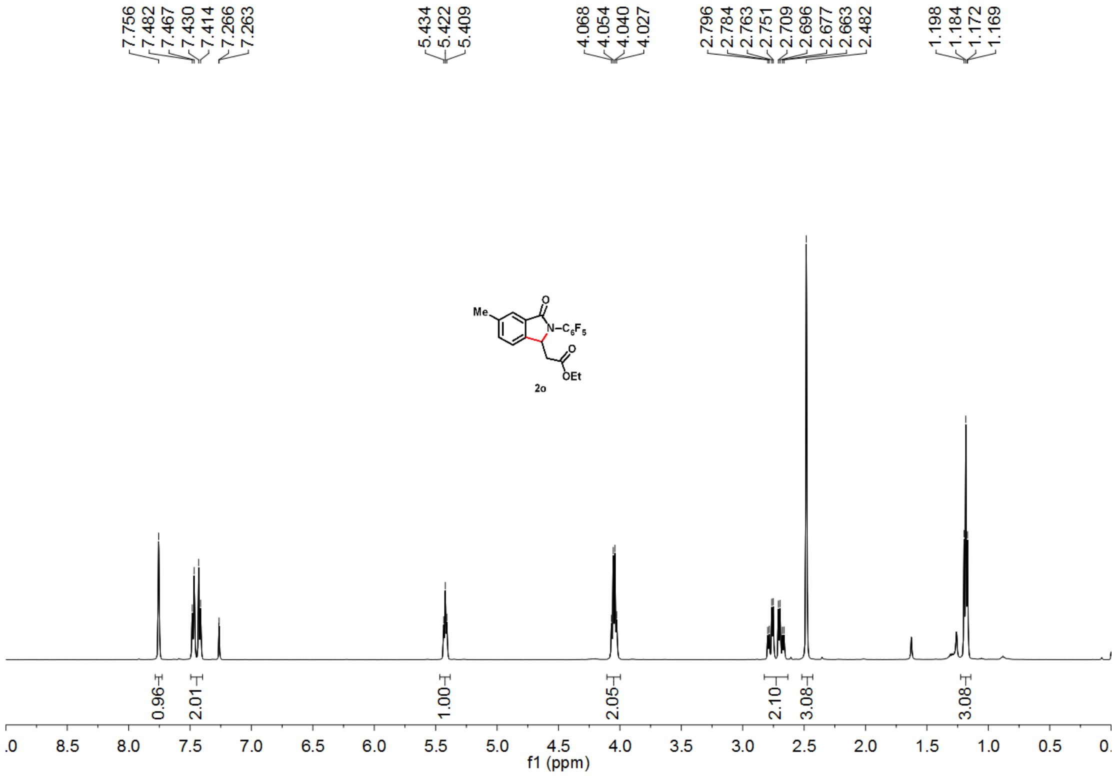
7.756
7.482
7.467
7.430
7.414
7.266
7.263
5.434
5.422
5.409
4.068
4.054
4.040
4.027
2.796
2.784
2.763
2.751
2.709
2.696
2.677
2.663
2.482
1.198
1.184
1.172
1.169
Me
O
N–C6F5
O
OEt
2o
0.96
2.01
1.00
2.05
2.10
3.08
3.08
f1 (ppm)

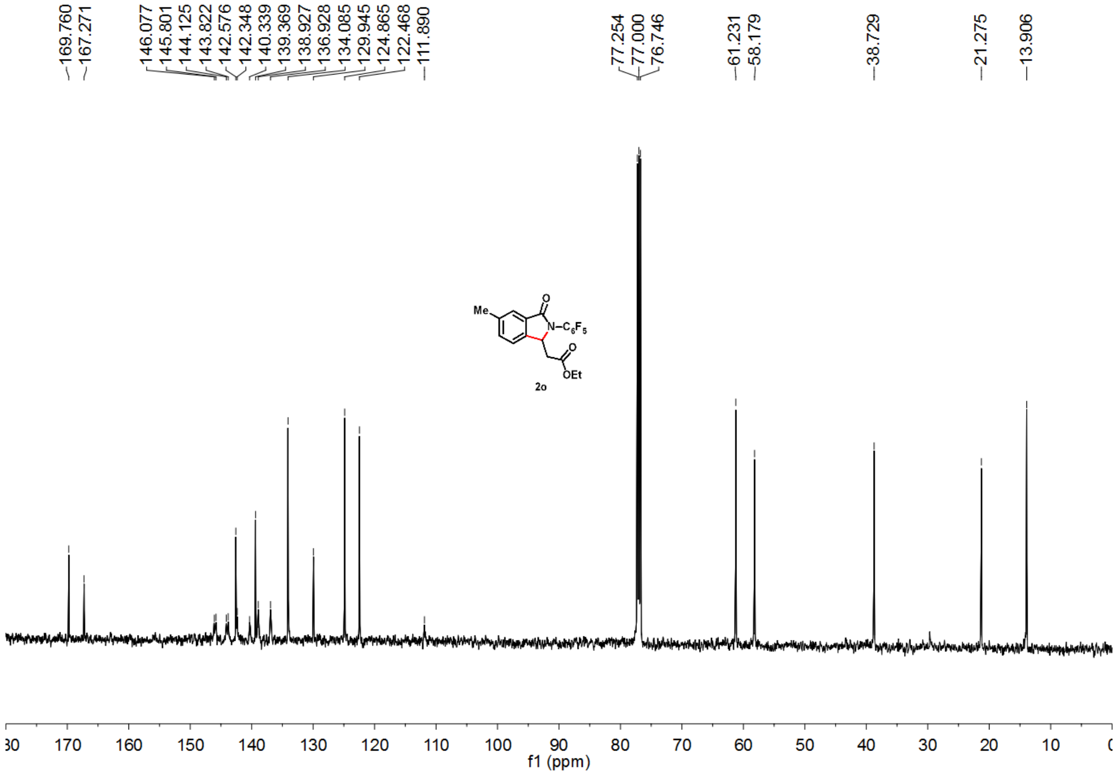
169.760
167.271
146.077
145.801
144.125
143.822
142.576
142.348
140.339
139.369
138.927
136.928
134.085
129.945
124.865
122.468
111.890
77.254
77.000
76.746
61.231
58.179
38.729
21.275
13.906
Me
O
N–C6F5
O
OEt
2o
f1 (ppm)

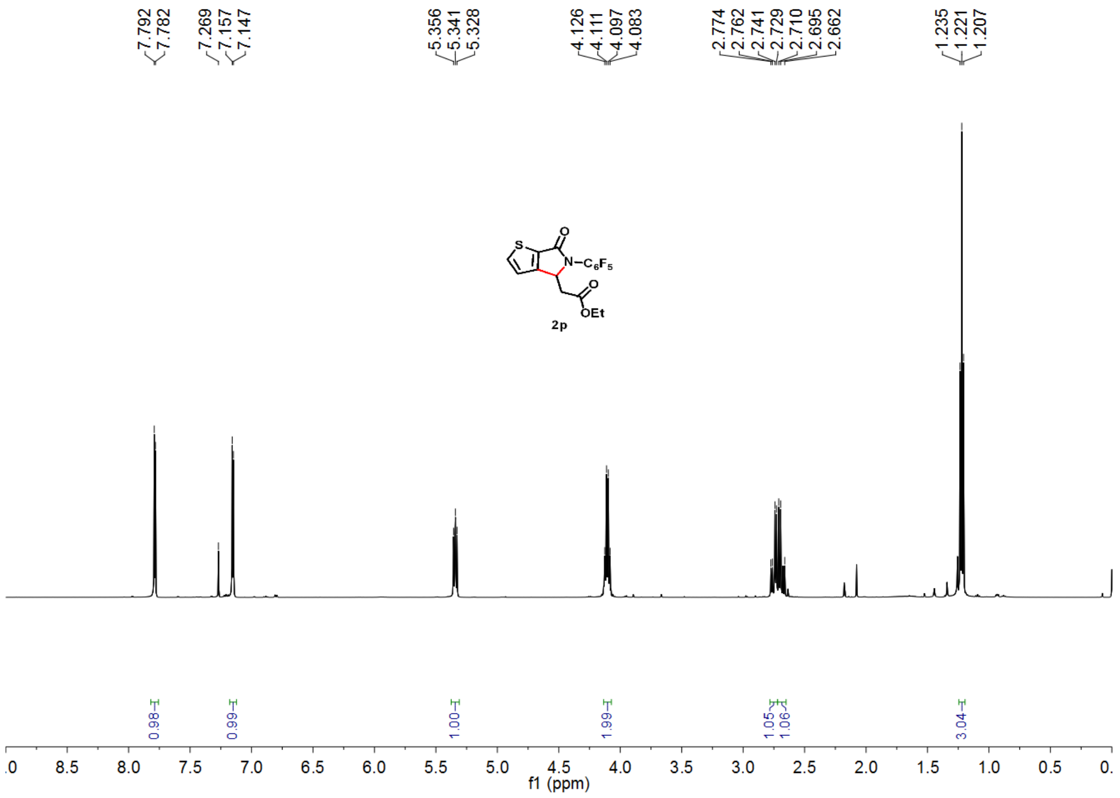
7.792
7.782
7.269
7.157
7.147
5.356
5.341
5.328
4.126
4.111
4.097
4.083
2.774
2.762
2.741
2.729
2.710
2.695
2.662
1.235
1.221
1.207
N−C6F5
OEt
2p
0.98
0.99
1.00
1.99
1.05
1.06
3.04
f1 (ppm)

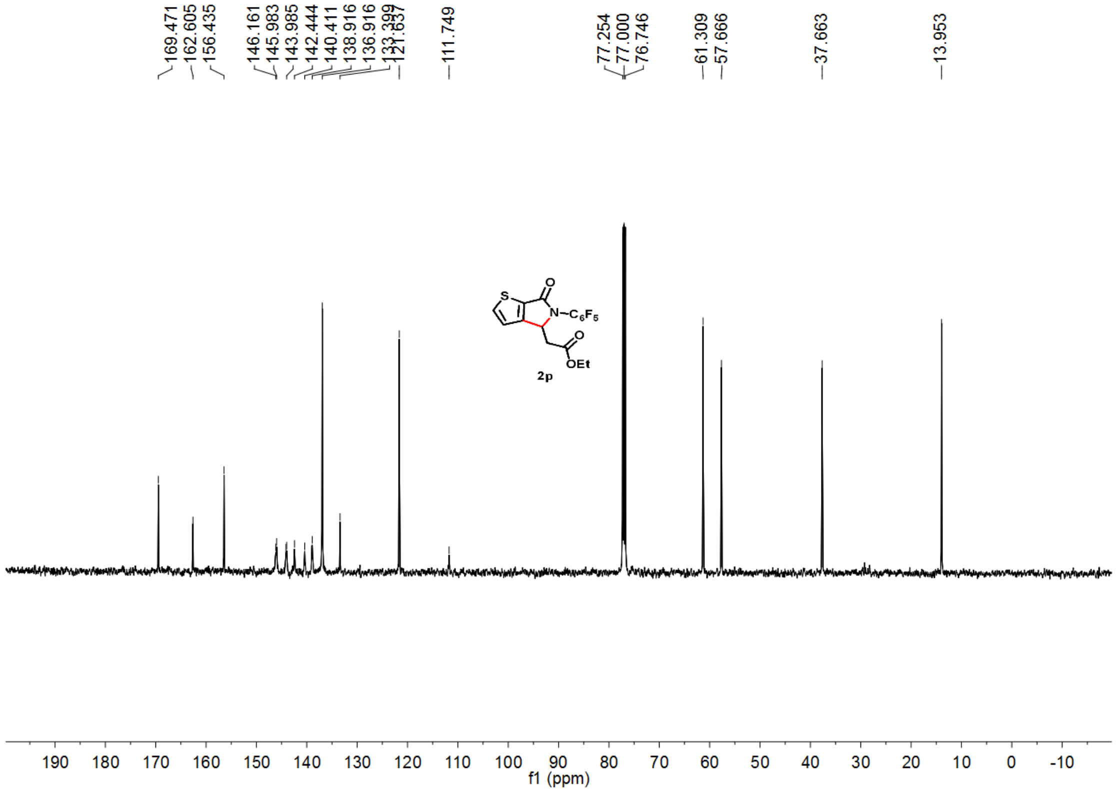
169.471
162.605
156.435
146.161
145.983
143.985
142.444
140.411
138.916
136.916
133.399
121.637
111.749
77.254
77.000
76.746
61.309
57.666
37.663
13.953
N−C6F5
OEt
2p
f1 (ppm)

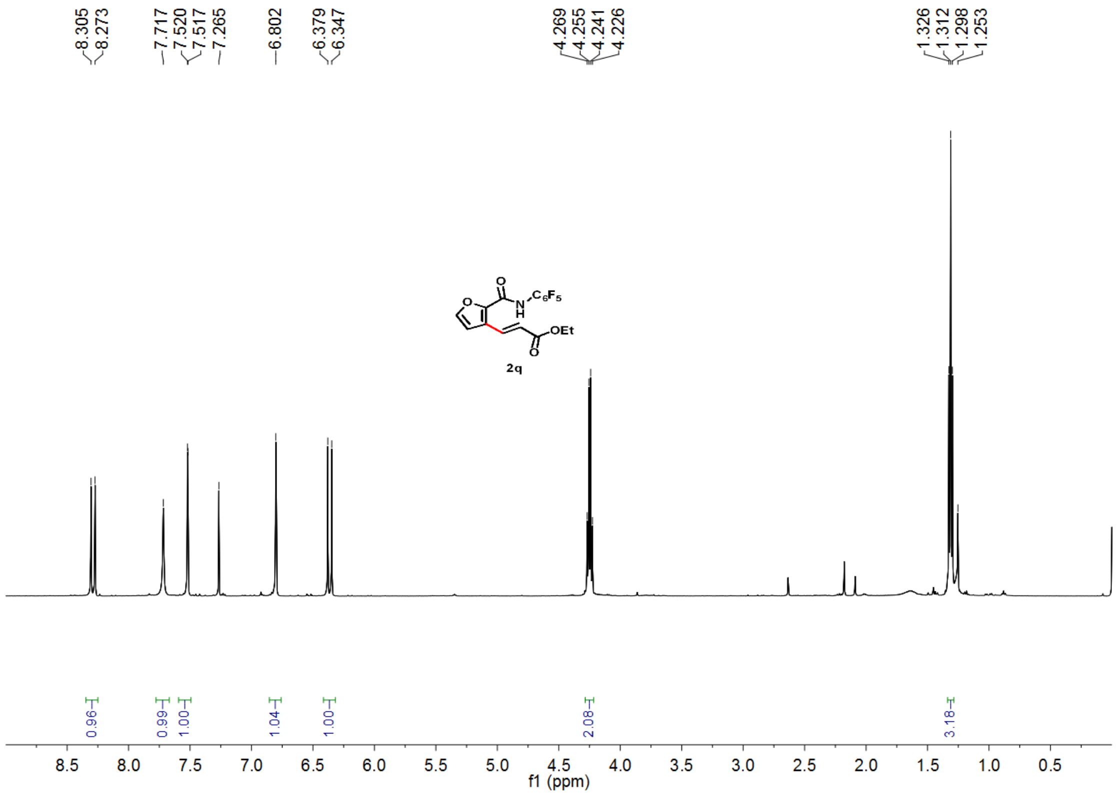
8.305
8.273
7.717
7.520
7.517
7.265
6.802
6.379
6.347
4.269
4.255
4.241
4.226
1.326
1.312
1.298
1.253
2q
0.96
0.99
1.00
1.04
1.00
2.08
3.18
f1 (ppm)

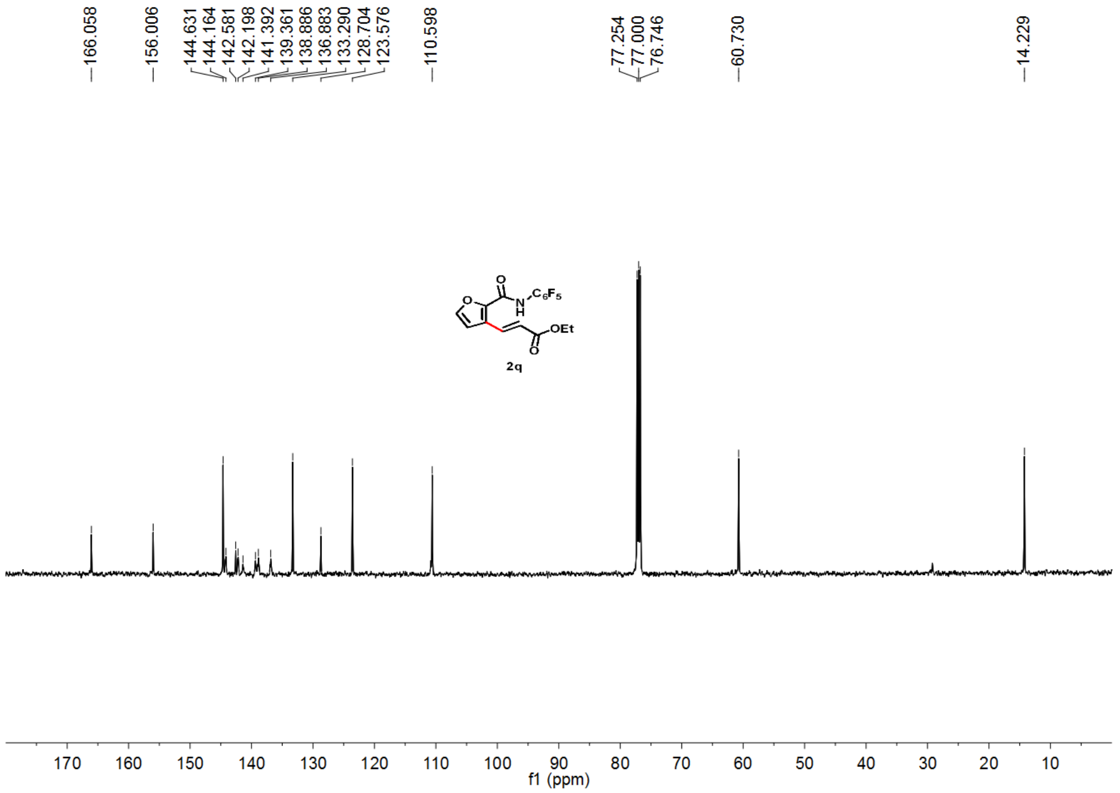
166.058
156.006
144.631
144.164
142.581
142.198
141.392
139.361
138.886
136.883
133.290
128.704
123.576
110.598
77.254
77.000
76.746
60.730
14.229
2q
f1 (ppm)

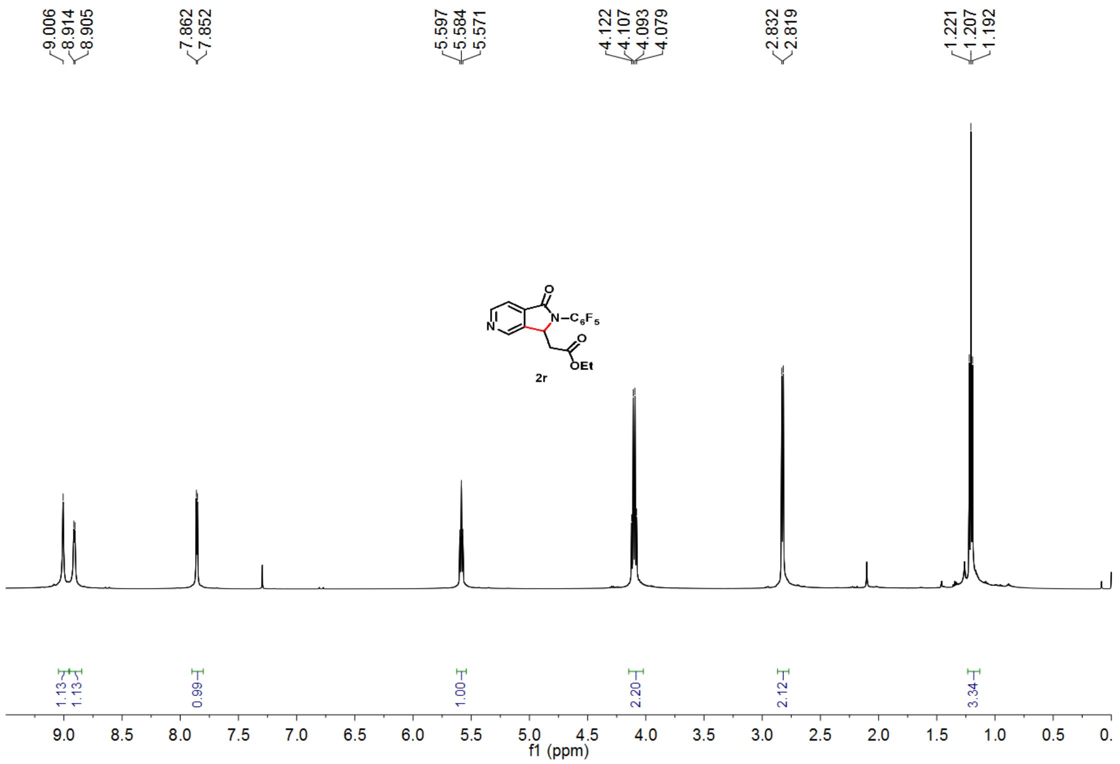

9.006
8.914
8.905
7.862
7.852
5.597
5.584
5.571
4.122
4.107
4.093
4.079
2.832
2.819
1.221
1.207
1.192
N
N−C6F5
O
OEt
2r
1.13
1.13
0.99
1.00
2.20
2.12
3.34
f1 (ppm)

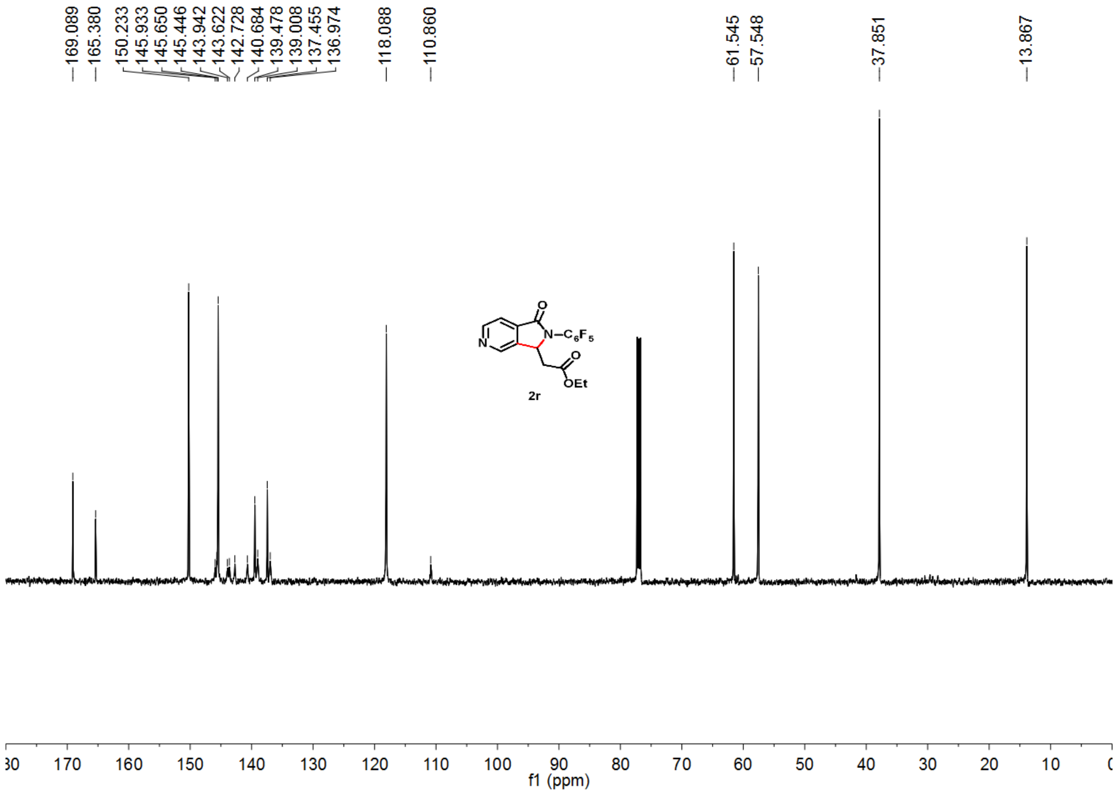

169.089
165.380
150.233
145.933
145.650
145.446
143.942
143.622
142.728
140.684
139.478
139.008
137.455
136.974
118.088
110.860
61.545
57.548
37.851
13.867
N
N−C6F5
O
OEt
2r
f1 (ppm)

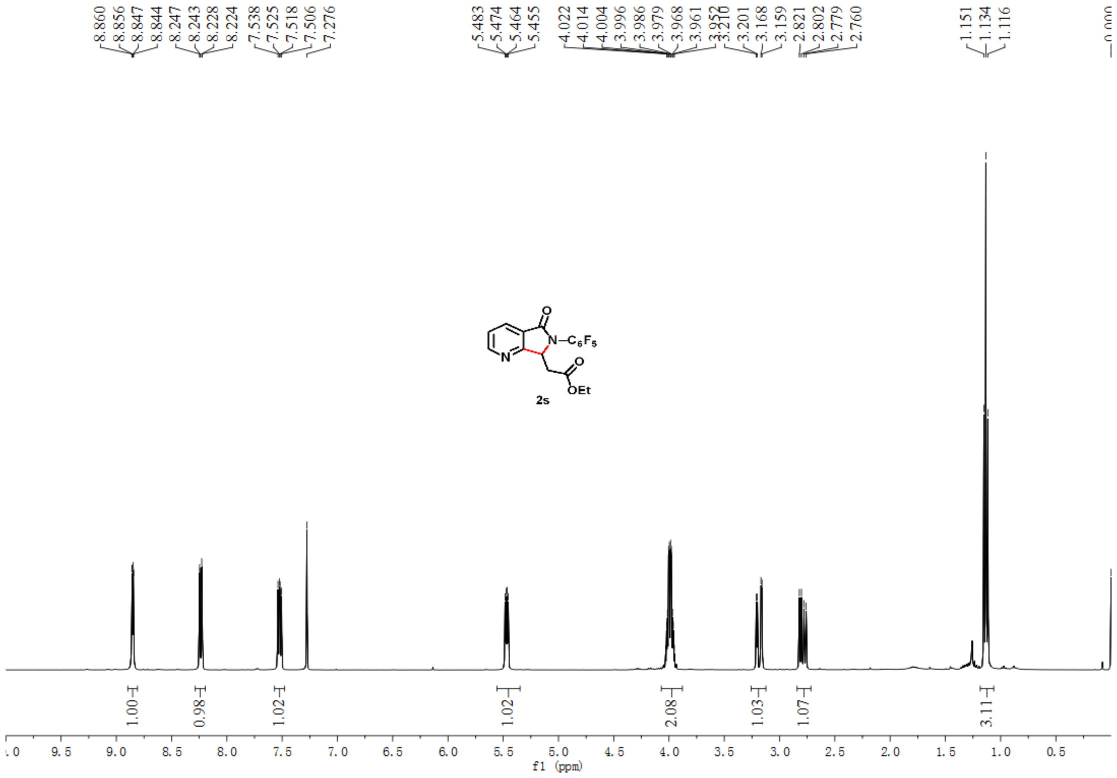

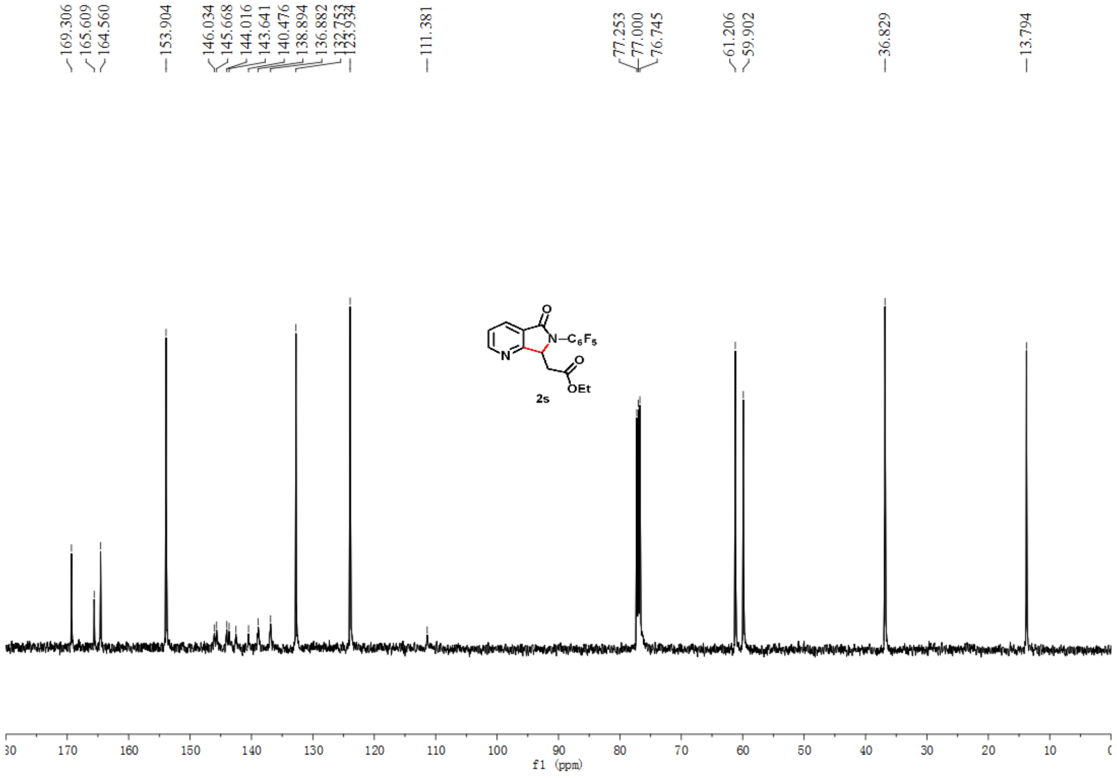

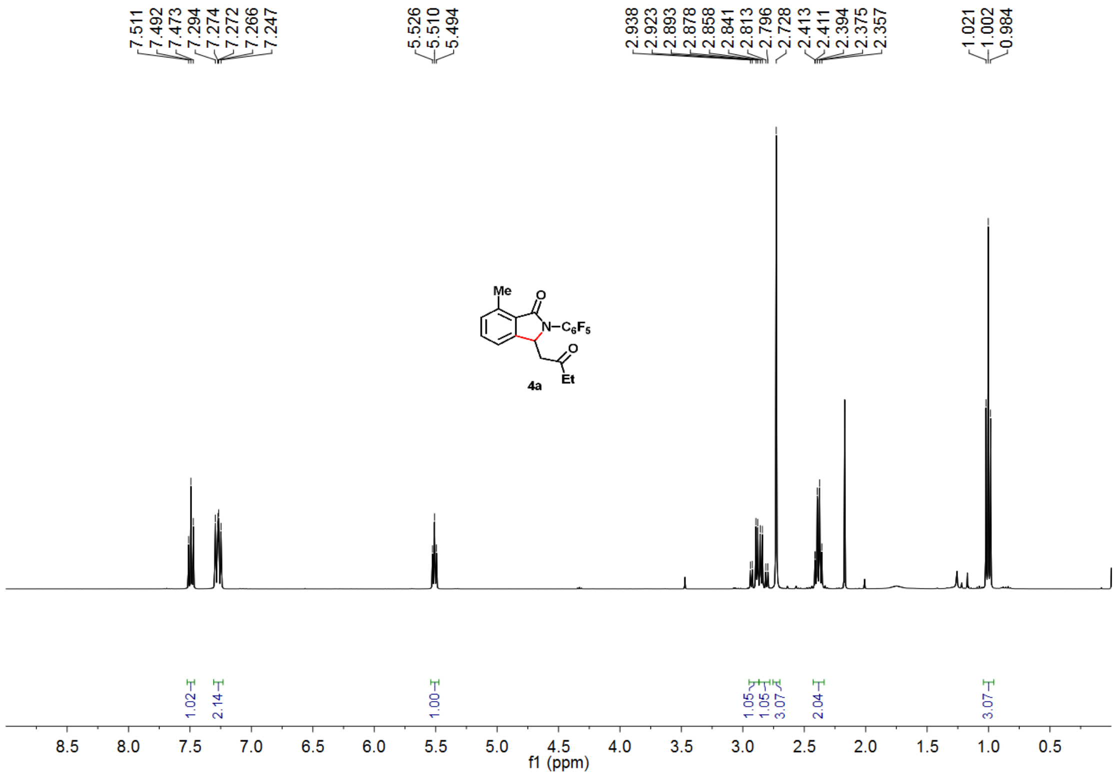
7.511
7.492
7.473
7.294
7.274
7.272
7.266
7.247
5.526
5.510
5.494
2.938
2.923
2.893
2.878
2.858
2.841
2.813
2.796
2.728
2.413
2.411
2.394
2.375
2.357
1.021
1.002
0.984
Me
O
N−C6F5
O
Et
4a
1.02
2.14
1.00
1.05
1.05
3.07
2.04
3.07
8.5
8.0
7.5
7.0
6.5
6.0
5.5
5.0
4.5
4.0
3.5
3.0
2.5
2.0
1.5
1.0
0.5
f1 (ppm)

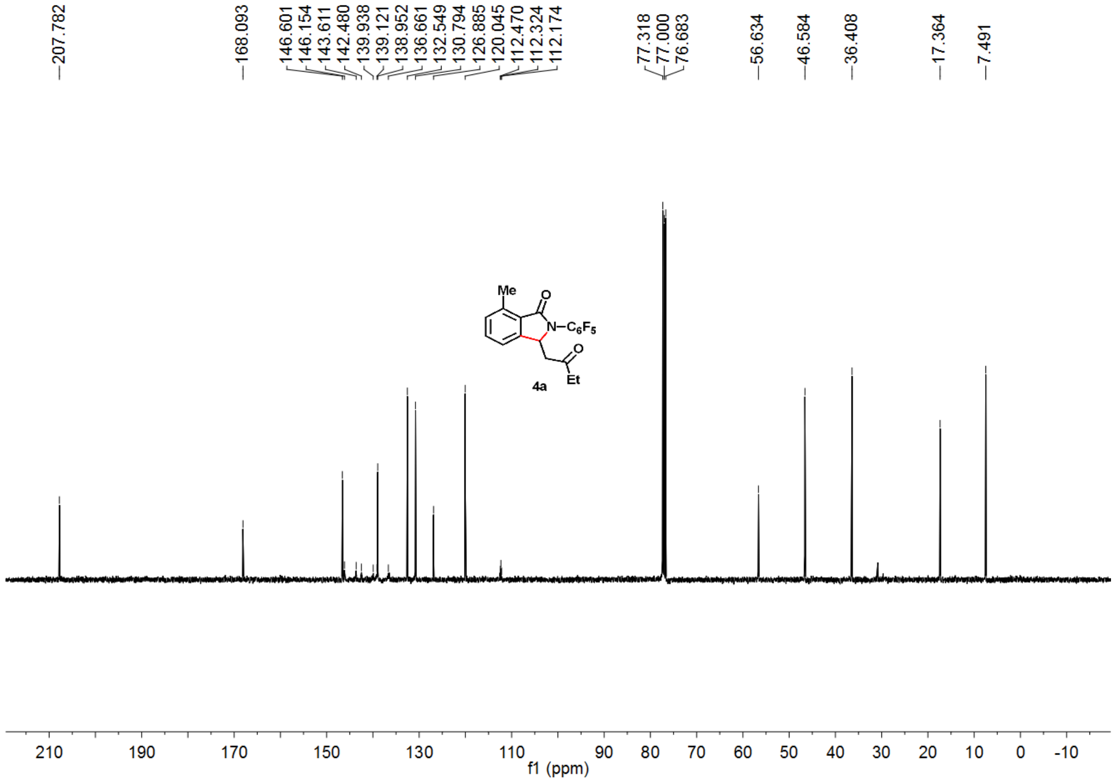
207.782
168.093
146.601
146.154
143.611
142.480
139.938
139.121
138.952
136.661
132.549
130.794
126.885
120.045
112.470
112.324
112.174
77.318
77.000
76.683
56.634
46.584
36.408
17.364
7.491
Me
O
N−C6F5
O
Et
4a
210
190
170
150
130
110
90
80
70
60
50
40
30
20
10
0
-10
f1 (ppm)

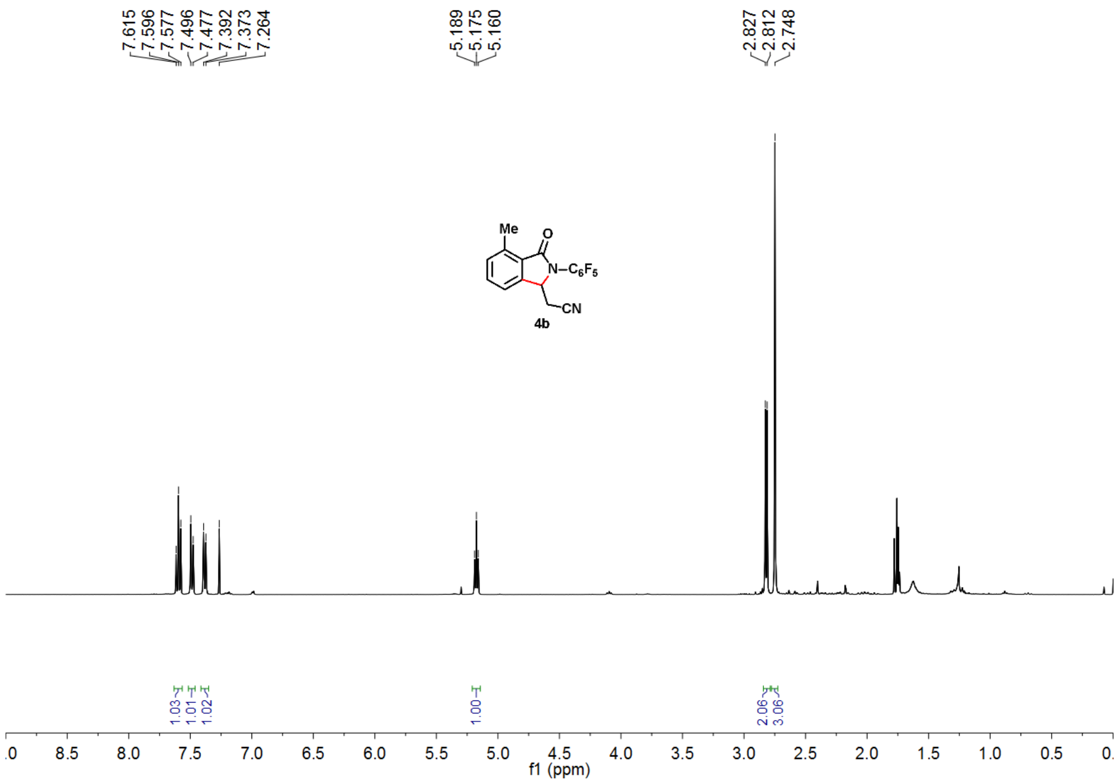
7.615
7.596
7.577
7.496
7.477
7.392
7.373
7.264
5.189
5.175
5.160
2.827
2.812
2.748
Me
O
N–C6F5
CN
4b
1.03
1.01
1.02
1.00
2.06
3.06
f1 (ppm)

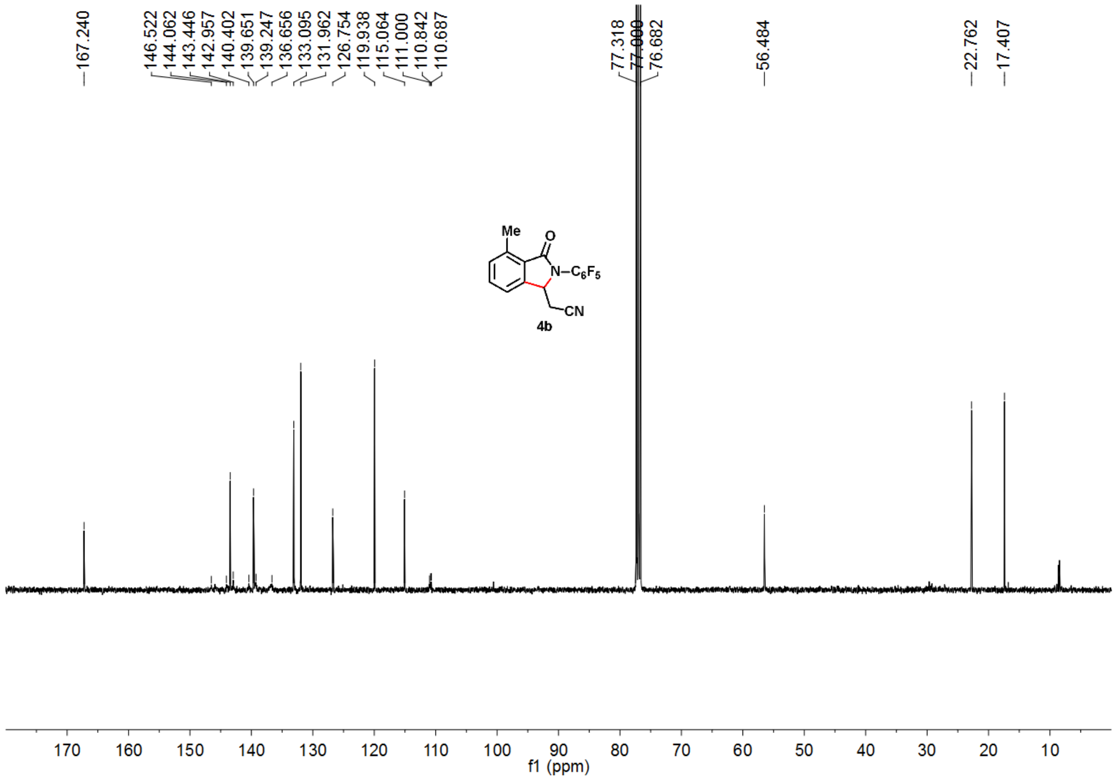
167.240
146.522
144.062
143.446
142.957
140.402
139.651
139.247
136.656
133.095
131.962
126.754
119.938
115.064
111.000
110.842
110.687
77.318
77.000
76.682
56.484
22.762
17.407
Me
O
N–C6F5
CN
4b
f1 (ppm)

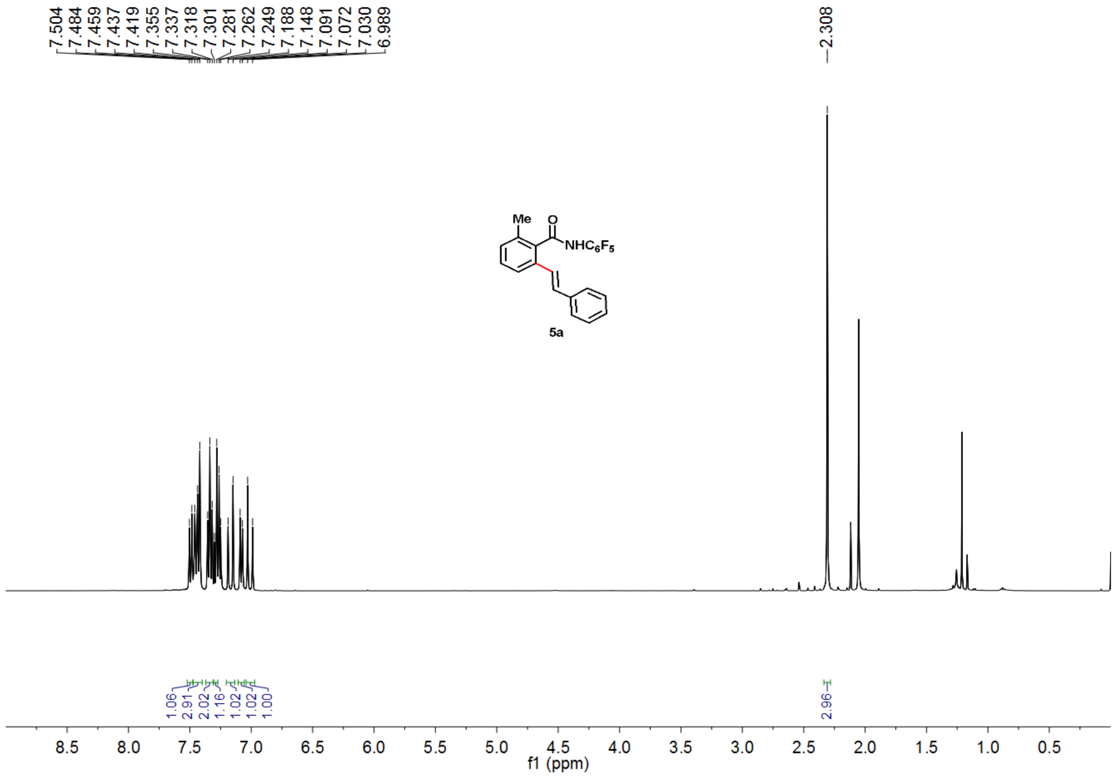
7.504
7.484
7.459
7.437
7.419
7.355
7.337
7.318
7.301
7.281
7.262
7.249
7.188
7.148
7.091
7.072
7.030
6.989
2.308
Me
O
NHC6F5
5a
1.06
2.91
2.02
1.16
1.02
1.02
1.00
2.96
f1 (ppm)

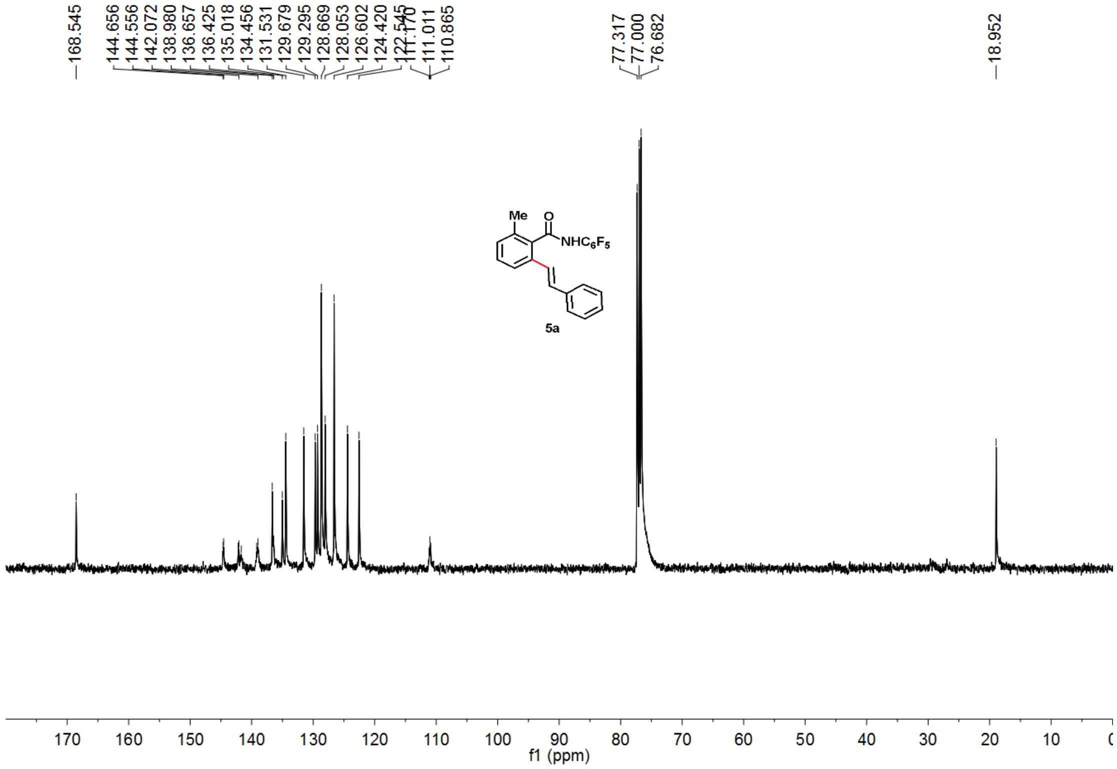
168.545
144.656
144.556
142.072
138.980
136.657
136.425
135.018
134.456
131.531
129.679
129.295
128.669
128.053
126.602
124.420
122.545
111.170
111.011
110.865
77.317
77.000
76.682
18.952
Me
O
NHC6F5
5a
f1 (ppm)

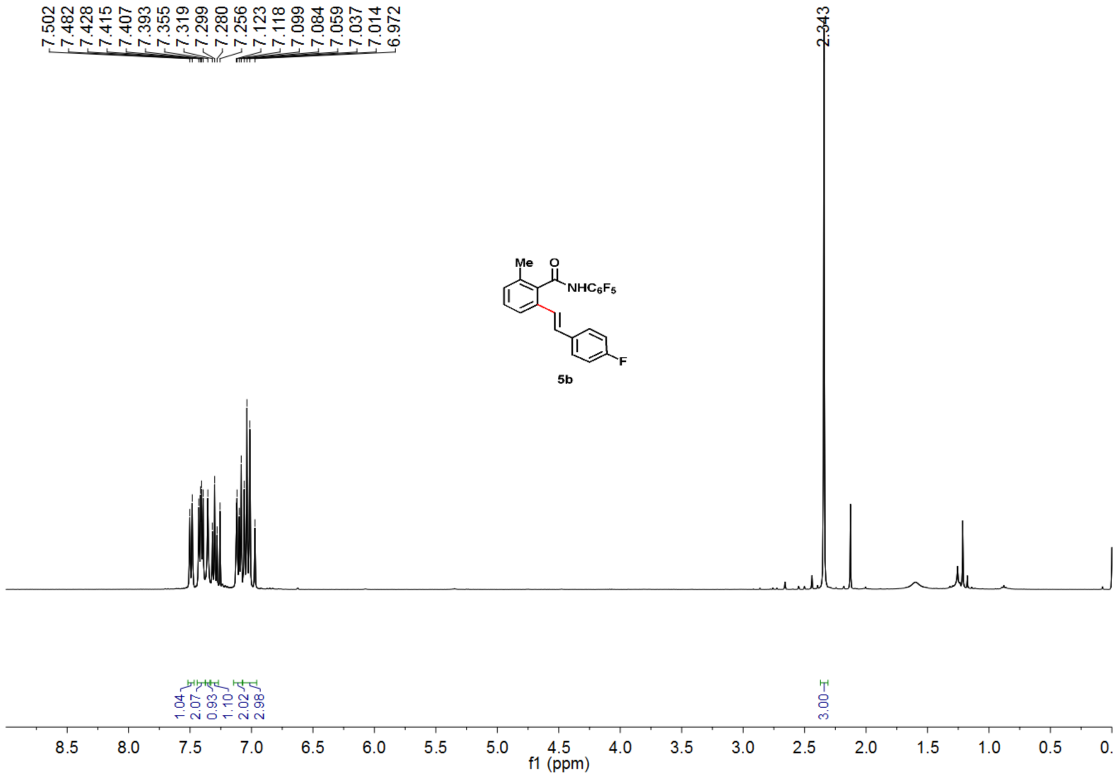
7.502
7.482
7.428
7.415
7.407
7.393
7.355
7.319
7.299
7.280
7.256
7.123
7.118
7.099
7.084
7.059
7.037
7.014
6.972
2.343
Me
O
NHC6F5
F
5b
1.04
2.07
0.93
1.10
2.02
2.98
3.00
f1 (ppm)

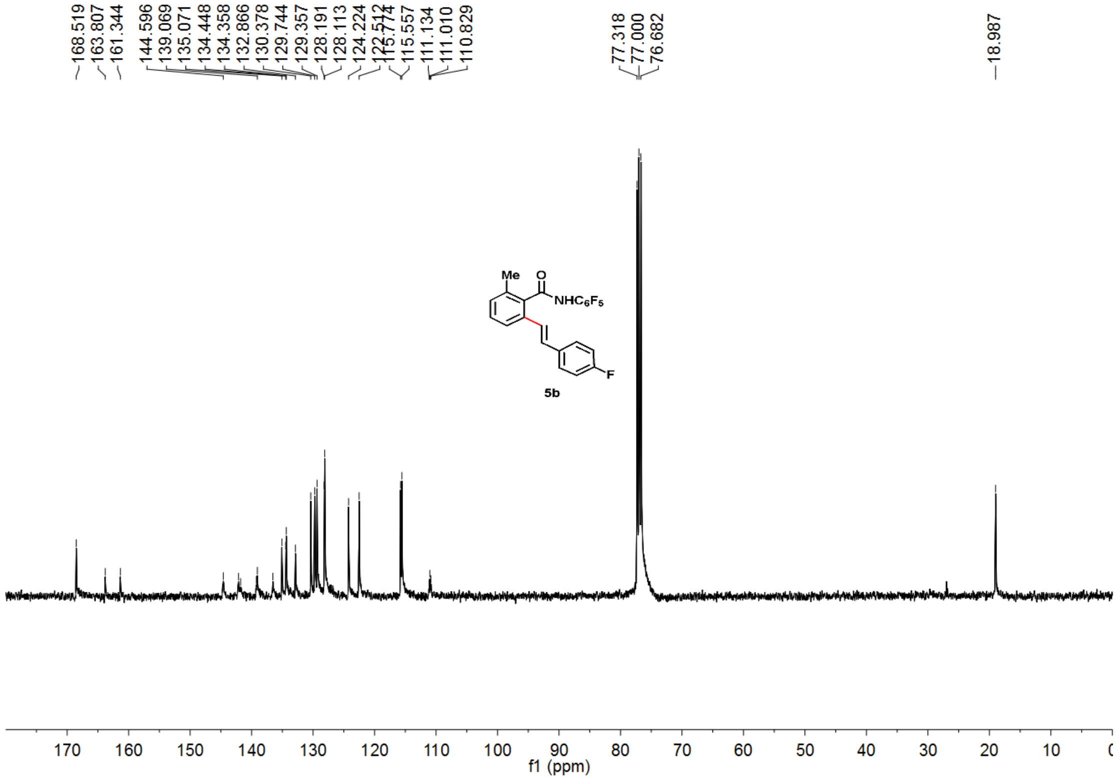
168.519
163.807
161.344
144.596
139.069
135.071
134.448
134.358
132.866
130.378
129.744
129.357
128.191
128.113
124.224
122.512
115.774
115.557
111.134
111.010
110.829
77.318
77.000
76.682
18.987
Me
O
NHC6F5
F
5b
f1 (ppm)

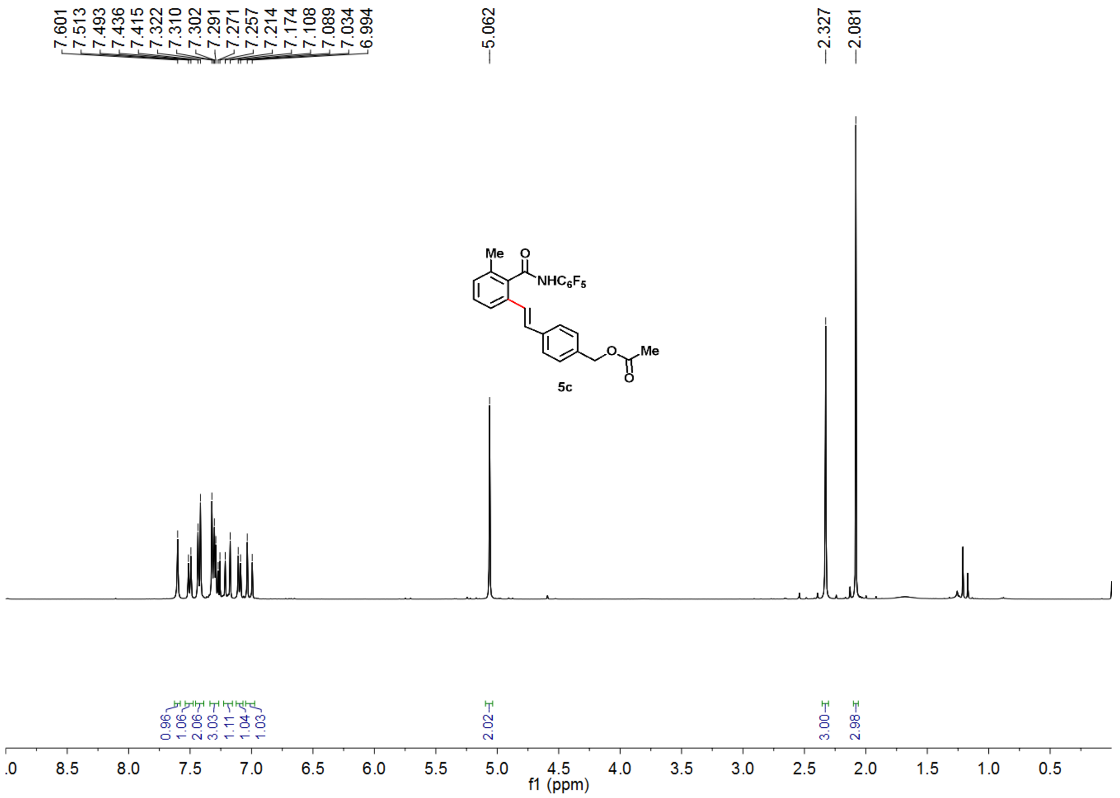

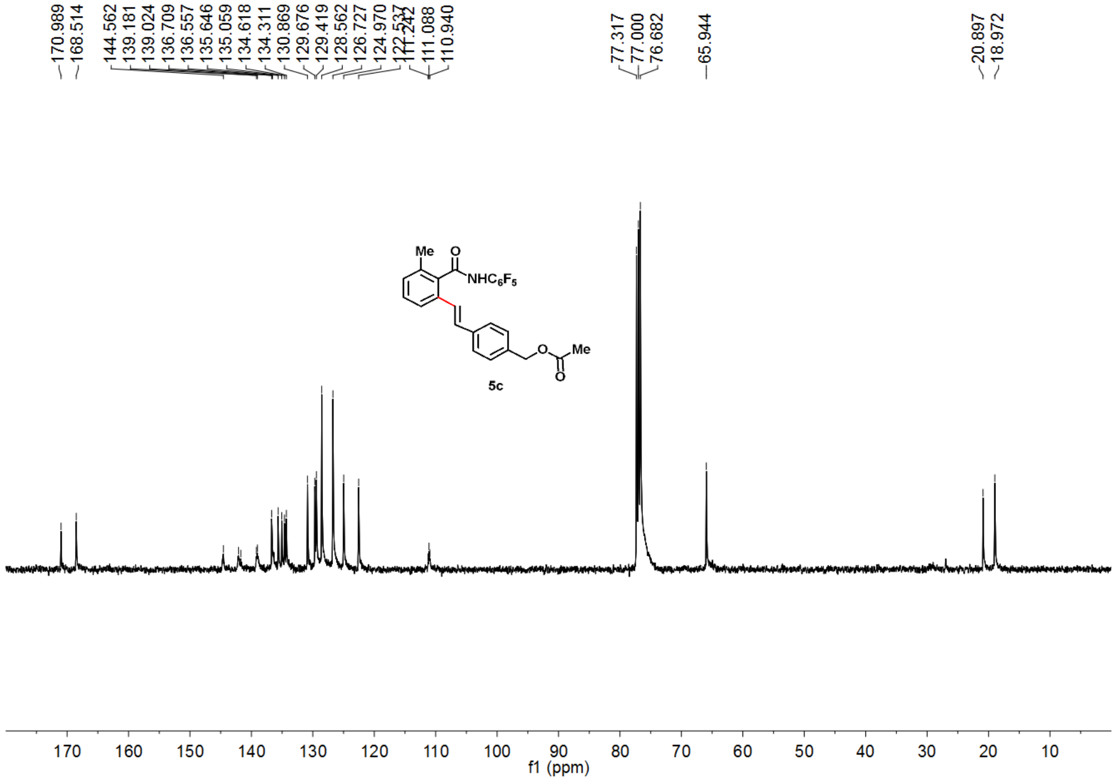

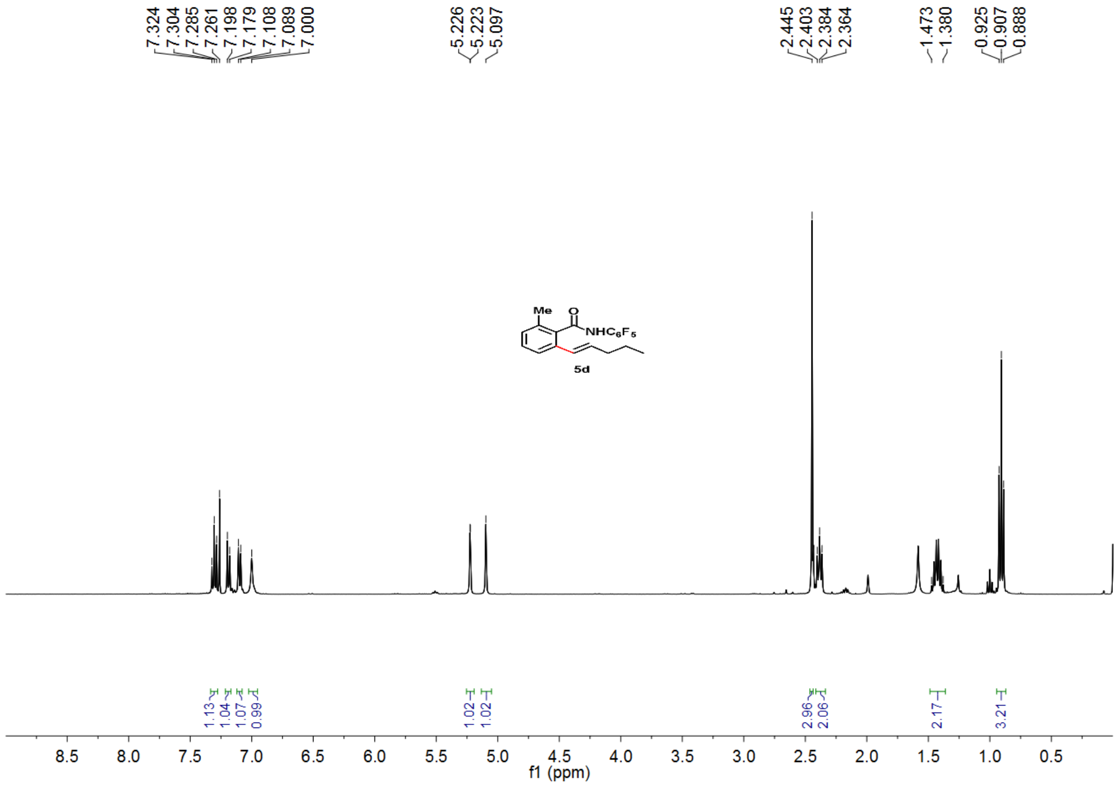
7.324
7.304
7.285
7.261
7.198
7.179
7.108
7.089
7.000
5.226
5.223
5.097
2.445
2.403
2.384
2.364
1.473
1.380
0.925
0.907
0.888
Me
O
NHC6F5
5d
1.13
1.04
1.07
0.99
1.02
1.02
2.96
2.06
2.17
3.21
8.5
8.0
7.5
7.0
6.5
6.0
5.5
5.0
4.5
4.0
3.5
3.0
2.5
2.0
1.5
1.0
0.5
f1 (ppm)

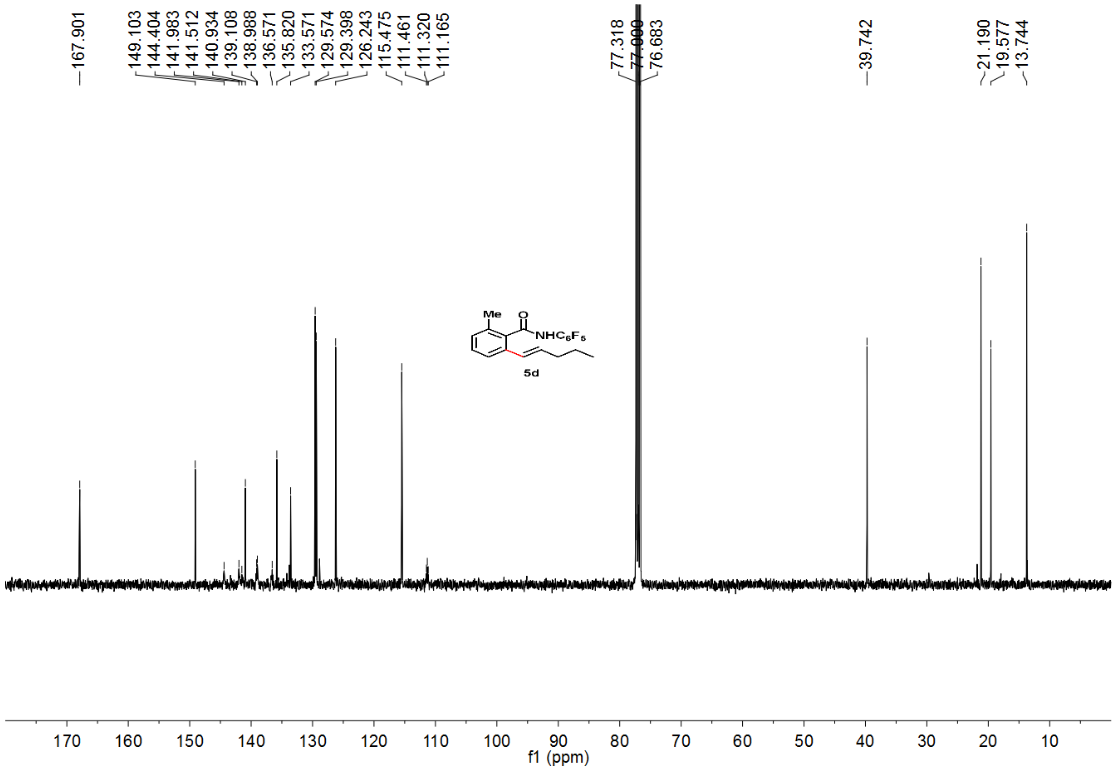
167.901
149.103
144.404
141.983
141.512
140.934
139.108
138.988
136.571
135.820
133.571
129.574
129.398
126.243
115.475
111.461
111.320
111.165
77.318
76.683
39.742
21.190
19.577
13.744
Me
O
NHC6F5
5d
170
160
150
140
130
120
110
100
90
80
70
60
50
40
30
20
10
f1 (ppm)

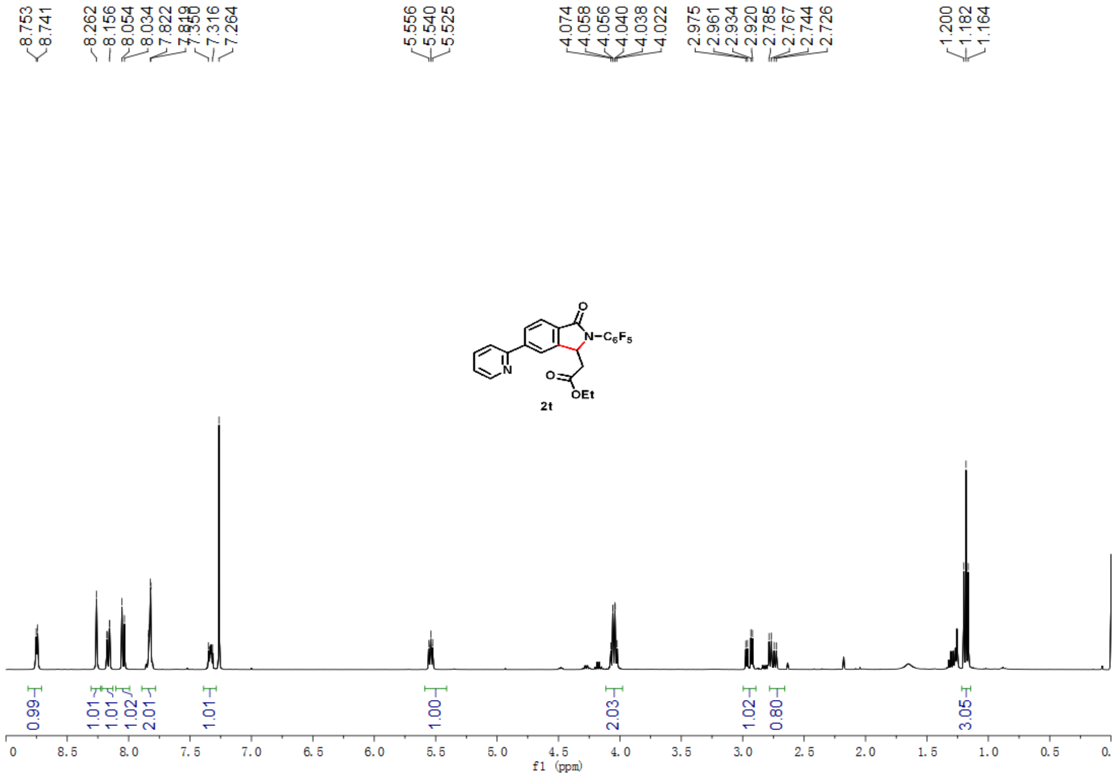
8.753
8.741
8.262
8.156
8.054
8.034
7.822
7.350
7.316
7.264
5.556
5.540
5.525
4.074
4.058
4.056
4.040
4.038
4.022
2.975
2.961
2.934
2.920
2.785
2.767
2.744
2.726
1.200
1.182
1.164
N−C6F5
OEt
2t
0.99
1.01
1.01
1.02
2.01
1.01
1.00
2.03
1.02
0.80
3.05
f1 (ppm)

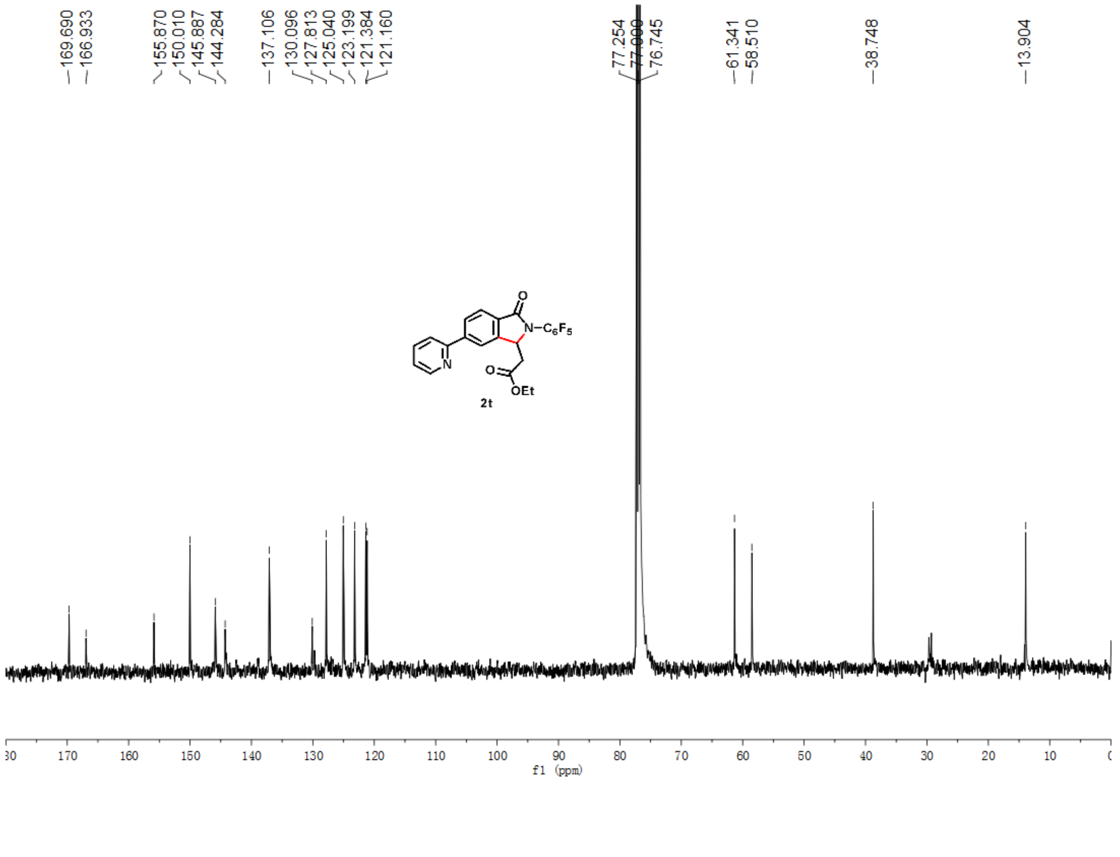
169.690
166.933
155.870
150.010
145.887
144.284
137.106
130.096
127.813
125.040
123.199
121.384
121.160
77.254
76.745
61.341
58.510
38.748
13.904
N−C6F5
OEt
2t
f1 (ppm)
